# Radiocarbon offsets and old world chronology as relevant to Mesopotamia, Egypt, Anatolia and Thera (Santorini)

**DOI:** 10.1038/s41598-020-69287-2

**Published:** 2020-08-17

**Authors:** Sturt W. Manning, Lukas Wacker, Ulf Büntgen, Christopher Bronk Ramsey, Michael W. Dee, Bernd Kromer, Brita Lorentzen, Willy Tegel

**Affiliations:** 1 Cornell Tree Ring Laboratory, Department of Classics, Cornell University, Ithaca, NY 14853 USA; 2 Laboratory for Ion Beam Physics, Swiss Federal Institute of Technology in Zurich, 8093 Zurich, Switzerland; 3 Department of Geography, University of Cambridge, Cambridge, CB2 3EN UK; 4 Swiss Federal Research Institute WSL, 8903 Birmensdorf, Switzerland; 5 Global Change Research Institute CAS, 603 00 Brno, Czech Republic; 6 Department of Geography, Faculty of Science, Masaryk University, 611 37 Brno, Czech Republic; 7 Research Laboratory for Archaeology, School of Archaeology, University of Oxford, Oxford, OX1 3TG UK; 8 Centre for Isotope Research, Faculty of Science and Engineering, University of Groningen, Nijenborgh 6, 9747 AG Groningen, The Netherlands; 9 Institute of Environmental Physics, University of Heidelberg, 69120 Heidelberg, Germany; 10 Chair of Forest Growth and Dendroecology, Institute of Forest Sciences, University of Freiburg, Freiburg, Germany; 11 Archaeological Service Kanton Thurgau (AATG), 8510 Frauenfeld, Switzerland

**Keywords:** Climate sciences, Environmental sciences, Environmental social sciences

## Abstract

The new IntCal20 radiocarbon record continues decades of successful practice by employing one calibration curve as an approximation for different regions across the hemisphere. Here we investigate three radiocarbon time-series of archaeological and historical importance from the Mediterranean-Anatolian region, which indicate, or may include, offsets from IntCal20 (~0–22 ^14^C years). While modest, these differences are critical for our precise understanding of historical and environmental events across the Mediterranean Basin and Near East. Offsets towards older radiocarbon ages in Mediterranean-Anatolian wood can be explained by a divergence between high-resolution radiocarbon dates from the recent generation of accelerator mass spectrometry (AMS) versus dates from previous technologies, such as low-level gas proportional counting (LLGPC) and liquid scintillation spectrometry (LSS). However, another reason is likely differing growing season lengths and timings, which would affect the seasonal cycle of atmospheric radiocarbon concentrations recorded in different geographic zones. Understanding and correcting these offsets is key to the well-defined calendar placement of a Middle Bronze Age tree-ring chronology. This in turn resolves long-standing debate over Mesopotamian chronology in the earlier second millennium BCE. Last but not least, accurate dating is needed for any further assessment of the societal and environmental impact of the Thera/Santorini volcanic eruption.

## Introduction

The 2020 International Northern Hemisphere (NH) Radiocarbon (^14^C) Calibration curve, IntCal20, forms the current basis to calendar ages for many scientific fields from 0 to 55 kyr ago^1,2^. IntCal20 continues the long-standing assumption that a single ^14^C calibration curve is applicable to the mid-latitudes of the NH^1–4^. However, there are indications of small, fluctuating, ^14^C offsets which, at high-resolution, may affect accurate ^14^C-based chronology in some mid-latitude regions^5–10^. Part of such differences may result from inter-laboratory offsets (see Supplementary Discussion 1), or derive from differences between recent AMS ^14^C measurements versus those from previous ^14^C dating technologies. Another part is inferred as a representation of the differing parts of the intra-annual atmospheric ^14^C cycle, recorded because of different plant growth seasons or contexts. An example of the latter is the difference between the growth period of tree rings in central and northern Europe and northern America that comprise the Holocene IntCal record (spring through summer), versus those of many plants in the Mediterranean and Near East (winter to early summer)^5–8,10^. The topic is noted, but is not addressed, in IntCal20^1,2^. Finally, there are latitude-based variations in ^14^C levels, but these are regarded as minimal within the mid-latitudes^3,4,11^. Here we show the presence of small, but varying, ^14^C offsets versus IntCal20—from one or a combination of the above potential sources—in the east Mediterranean-Anatolia region across the second millennium BCE. These need to be addressed to achieve accurate high-resolution ^14^C-based chronology (and revise and clarify indications from initial comparisons with earlier versions of IntCal^5–8,10^). While small, the impact of these ^14^C offsets can be substantial for Mediterranean and Near Eastern archaeology because of the intricate and densely integrated timeframes involved and the small margins of tolerance^2,7,8,10,12^. Moreover, where present, apparent seasonal ^14^C offsets fluctuate over time, and appear associated with changes in ^14^C production and thus likely with variations in solar activity and climate (and ocean systems), and potentially also, therefore, changes in percentage contributions of early and late wood to given tree-rings^5,8–10,13^. These circumstances complicate the elegant hypothesis of a single NH calibration curve, with any variation assumed as effectively comparable with (or incorporated within) error terms^2^. However, as we illustrate for Old Assyrian/Old Babylonian chronology, it opens the way for more accurate and precise dating through recognition of offsets and by tying sequences to specific appropriate ^14^C records.

Among explanations for offsets between ^14^C measurements, the least recognized is the role of the intra-annual cycle of atmospheric ^14^C levels, with an NH winter low and a summer high^5,6,8–10,14^. The Holocene part of the NH IntCal20 ^14^C calibration curve, constructed mainly from tree-rings from central and northern Europe and northern America, reflects photosynthesis in the spring through summer period^1^. In contrast, many plants in lower elevation contexts in the Mediterranean and Near East grow primarily in winter to spring^6,8,10^, or exhibit plasticity allowing climate and growth environment to modulate the boundaries of their growing season from year to year^15^. Hence, there is a potential for different aspects of the annual ^14^C cycle to be represented, especially as measurement of ^14^C increases in accuracy and precision^6–10^. Despite a few observations of regional differences^5,6,11,13,16^, the topic really only became visible and relevant a decade ago in a large-scale study addressing ancient Egypt^7^. This demonstrated that ^14^C-based dating could achieve accuracy and precision at the level of the approximate historically derived chronology of Egypt. However, the data indicated it was necessary to make allowance for an Egyptian offset in local ^14^C levels^6, 7^. This offset was associated with the different (near opposite) growing season for plant matter in pre-modern Egypt (winter–spring) versus the growing season for the tree-rings used to inform the NH IntCal calibration record (spring–summer). Other work has identified instances of small offsets for the Mediterranean-Near East region, but also indications that they fluctuate^5,8,10,13^.

Whereas Libby employed samples from Old World archaeology to help supply a ‘curve of knowns’ to initially validate ^14^C dating^17^, we now employ data from archaeo-historic cases with tight constraints to explore the issue of ^14^C offsets, including any Mediterranean-Near East ^14^C offset. Based on existing observations, ^14^C offsets are typically evident only over certain periods, and become visible in the context of longer high-resolution rigid or near-rigid time-series^5–8,10,13^. Here we report comparison and analysis of three high-resolution ^14^C time-series from archaeological material from the Mediterranean-Anatolia region against the IntCal20 dataset to identify and quantify ^14^C offsets and to discuss sources. Historical chronologies provide constraints; in turn, they are better dated.

## Results

### Anatolian Middle Bronze Age tree ring radiocarbon time series versus IntCal20

The first ^14^C time-series comprises samples from a Middle Bronze Age (MBA) juniper (*Juniperus* sp.) tree-ring chronology constructed from three archaeological sites in Anatolia (Acemhöyük, ACM, Karahöyük, KBK, and Kültepe, KUL), archaeologically associated with Old Assyrian/Old Babylonian history through texts naming rulers and officials from the earlier second millennium BCE^18,19^. This confluence of evidence enables potential resolution of the long-running debate over Mesopotamian chronology, where text and astronomical data have offered possibilities but not definitive solutions^20^. Previous work indicated a likely solution^18,21^. New data improving and extending the MBA ^14^C time series, and the availability of the revised IntCal20 ^14^C calibration dataset for comparison, provide the context to revisit in order to establish a high-resolution placement. We use the existing data^18^ and incorporate 25 new ETH measurements (Supplementary Table S1). Since the wood samples from each site crossdate to form a single secure annual tree-ring chronology^18^, the tree-ring sequenced series of ^14^C data (n = 76) over a 200-year period should offer close comparison with the NH ^14^C calibration curve. We compare and fit (‘wiggle-match’) the data using the known tree-ring spacing after removing four initial outliers using the OxCal software^22–24^ (see “Methods”).

**Figure 1:**
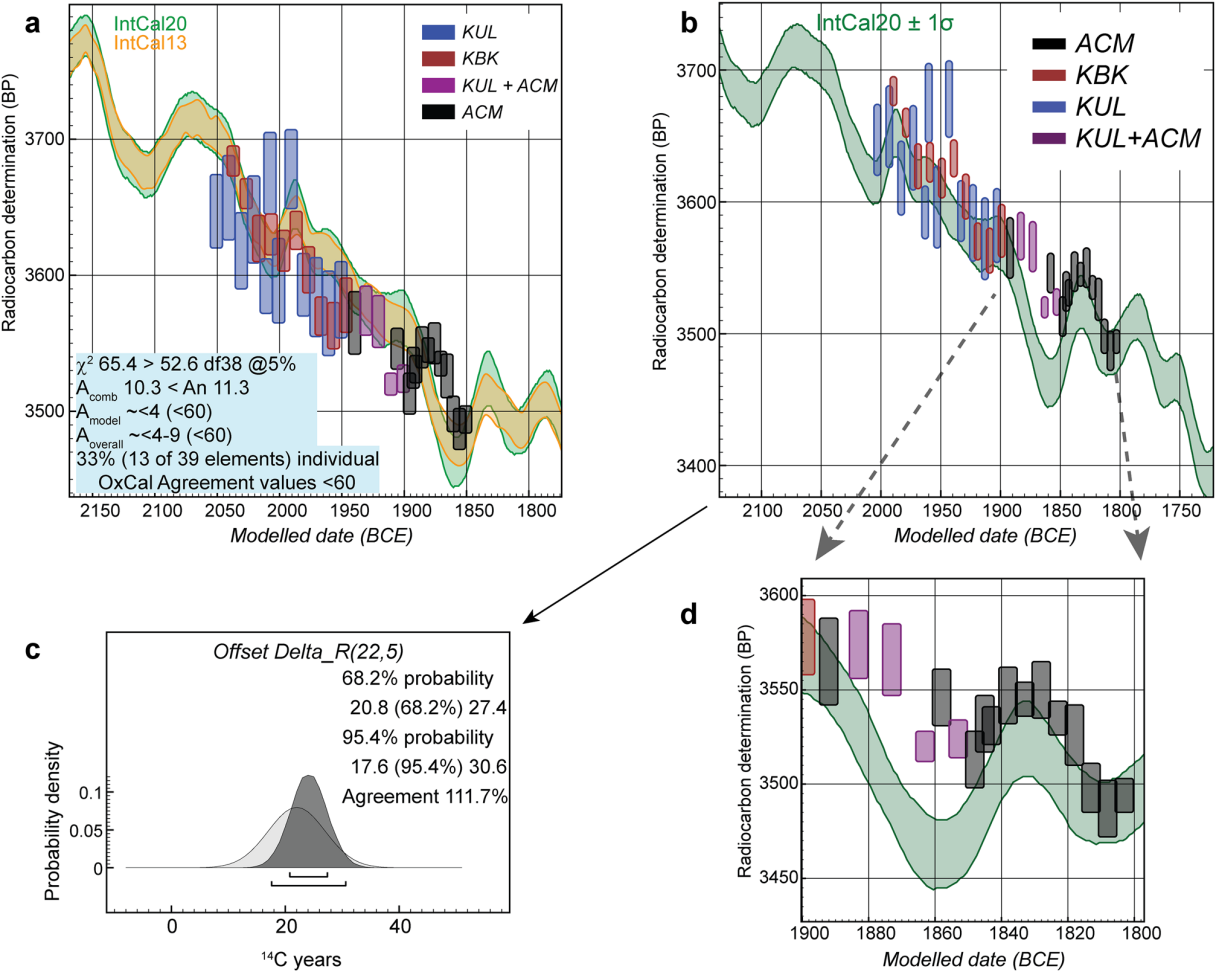
Fit of the MBA crossdated tree-ring ^14^C time series from Acemhöyük (ACM), Karahöyük (KBK) and Kültepe (KUL)^18^ against IntCal20^1^. (**a**) Wiggle-match with OxCal^22,23^ 4.4.1 of the MBA ^14^C time series against IntCal20 with no offset allowed for and curve resolution of 1 year (the previous IntCal13 calibration curve^27^ is shown for comparison). The OxCal A_model_ and A_overall_ values are poor and 33% of the data achieve unsatisfactory individual OxCal Agreement values (< 60). Visual inspection shows most data are placed too old, so they are either below the calibration curve or do not offer good correspondence—especially the set of Acemhöyük dates (black) which show structure, but do not correspond with the calibration curve at this calendar position. (**b**) Fit with an offset of 22 ± 5 ^14^C years. 72 data, 39 elements. (**c**) Modelled posterior density (dark histogram) versus the prior of 22 ± 5 ^14^C years illustrating good agreement (see Supplementary Fig. S1). (**d**) Close and specific fit of the ACM ^14^C data (black) around the wiggle in IntCal20 between 1,850 and 1,810 BCE. Data ~ 1,890 to 1,850 BCE, during a reversal in atmospheric ^14^C levels, indicate a likely (positive) regional or measurement ^14^C offset.

However, the fit is poor, failing an overall χ^2^ test and yielding poor OxCal agreement indices (Fig. 1a). An OxCal ΔR test^24^, to assess whether there is systematic difference between the MBA time series and the calibration curve using a neutral prior (0 ± 10 ^14^C years), indicates in many cases a bimodal finding (Supplementary Fig. S1). The data are offset on average either (and most likely) about 22 ± 5 ^14^C years, or, alternatively about − 32 ± 8 ^14^C years. Let us quantify what these differences mean in calendar terms for a specific point in the MBA tree ring series, Relative Year (RY) 701 (the latest dated element), in order to appreciate the scale of the problem. The mid-point of the 68.3% highest posterior density (hpd) range for RY701 with no ΔR is ~ 1,851 BCE, with ΔR 22 ± 5 ^14^C years it is ~ 1,803 BCE and with ΔR − 32 ± 8 ^14^C years it is ~ 1,883 BCE—a total range of ~ 81 calendar years. Such a large discrepancy is incompatible with high-resolution chronology. It is therefore important to resolve such ambiguity and imprecision. To investigate towards the likely solution, we tried wiggle-matches incorporating an offset effect of 22 ± 5 ^14^C years or − 32 ± 8 ^14^C years. Runs of the latter model yield poor OxCal agreement indices (A_model_ and A_overall_ below 30, well below the satisfactory threshold value of 60), the posterior density for the ΔR offers poor OxCal agreement with the prior (< 60), and there is a poor visual fit (Supplementary Fig. S2). We thus exclude this option as not viable. In contrast, the model incorporating an offset effect ΔR of 22 ± 5 ^14^C years offers a good visual fit with IntCal20 (A_model_ and A_overall_ around 60) (Fig. 1b) and the observed ΔR corresponds successfully with this prior estimate (Fig. 1c). In particular, although offset to slightly older ^14^C ages, we note how the MBA series as placed in Fig. 1b closely describes the wiggle ~ 1,850 to 1,810 BCE in the IntCal20 calibration curve (Fig. 1d, Supplementary Fig. S3). This provides a specific and secure chronological placement for the later part of the time series, versus a lack of clarity in this region with a smaller dataset and previous calibration curves^18,21^. Thus, by identifying, quantifying and then exploiting the relevant offset in this case we can obtain a unique high-resolution chronology.

The incompatibility (older ^14^C values) of the four KUL + ACM elements ~ 1,883 to 1,853 BCE with IntCal20 is conspicuous. To investigate, we measured new ETH data on known-age single-year oak tree-ring samples from Erstein, France, from part of this period (Supplementary Table S2)^25,26^. These data also do not replicate the strong dip and reversal in IntCal ~ 1,860 to 1,840 BCE (~ 3,809 to 3,789 Cal BP). Instead, they indicate values that are older than IntCal20 and more in the range of those from the MBA time series. Collectively, these new data suggest that IntCal itself needs some revision in this period (Supplementary Fig. S4). Hence, while some portion of the visible offset in this case might, as in cases of other reversals in the ^14^C record^8,10^, comprise a manifestation of a regional ^14^C offset, in this instance the actual existence of the strong reversal in the IntCal dataset is open to question. We re-run the wiggle-match of the MBA time series excluding this currently problematic interval to check that it is not being unduly influenced by this issue. We thus exclude the five offset data points for RY621, RY631, RY641, RY646 and RY651 (Fig. 1d). Over 10 runs with a neutral prior of 0 ± 10 ^14^C years, the remaining MBA time series nonetheless consistently finds the same approximate best fit range as in Fig. 1b (in 5 of 10 runs, or 1 year older, in 4 of 10 runs, or 2 years older, in 1 of 10 runs). Further, within 95.4% probability limits, the reduced time series now avoids the bi-modal probability issue noted above (Supplementary Fig. S1)—we may therefore view the previous ambiguity as caused by the problematic dip in the current IntCal dataset. With the edited time series, the OxCal ΔR offset observed is reduced a little—but nevertheless remains present. The average 68.3% hpd ΔR offset range is 17.0 ± 4.1 ^14^C years. If the series is then run with a ΔR of 17 ± 4 ^14^C years, it consistently finds a very similar but slightly better defined best fit placement compared to that shown in Fig. 1b. The mean placement of the elements is just 0–1 year later and the standard deviation on this mean is 1 year smaller (2 versus 3). The last dated RY701 element is placed 1,805–1,800 BCE (68.3% hpd) and 1,807–1,798 BCE (95.4% hpd), compared with 1,806–1,801 BCE (68.3% hpd) and 1,809–1,797 BCE (95.4% hpd) in the Fig. 1b fit using ΔR 22 ± 5 ^14^C years. We therefore regard the placement shown in Fig. 1b as robust within about 1 year, pending revision of this whole period of the IntCal dataset (we note that this portion of IntCal20 remains largely based on legacy data from IntCal13^27^, and before).

### Mesopotamian Old Assyrian/Old Babylonian chronology

**Figure 2:**
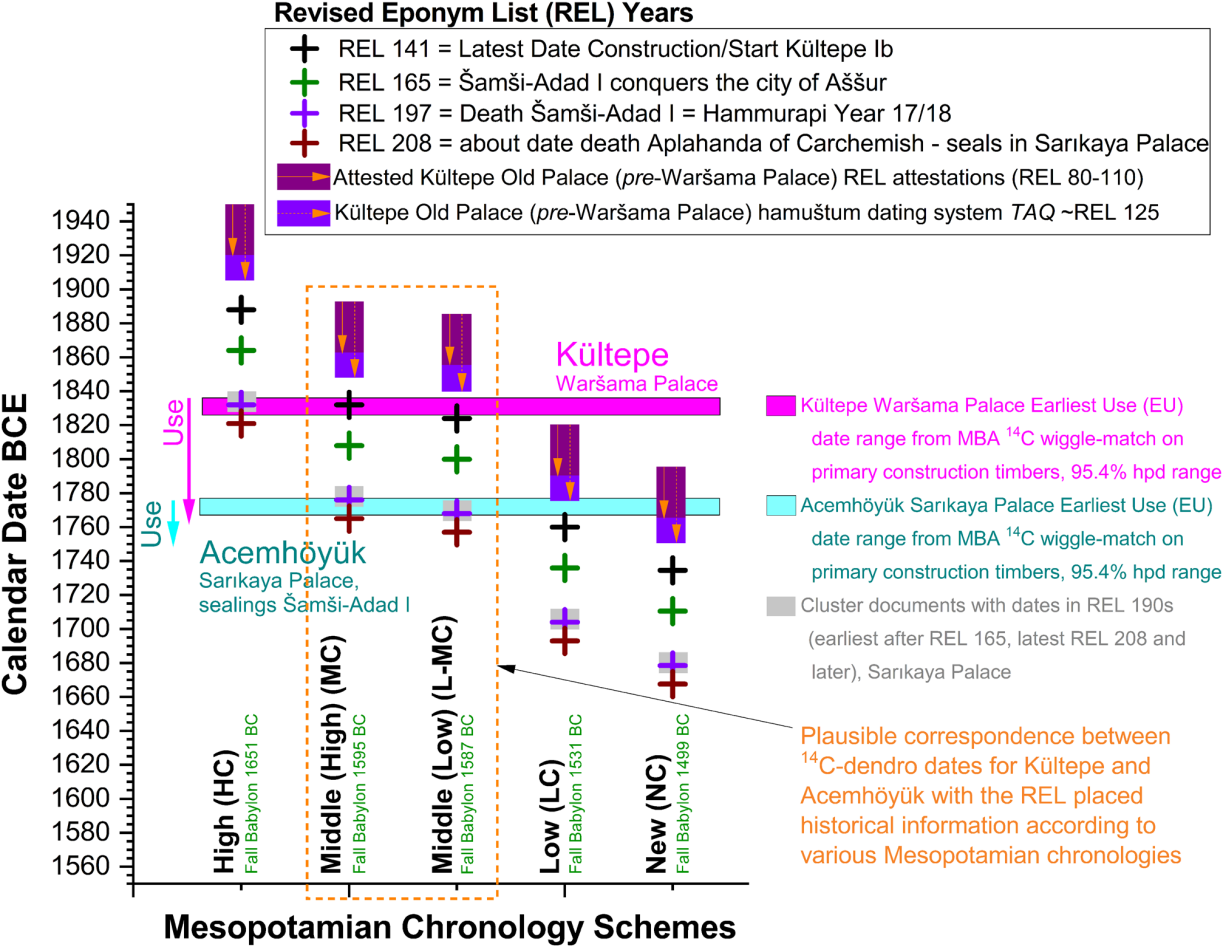
Comparisons of sequenced ^14^C datasets and their historical associations. (**a**) Earliest use dates for the Waršama Palace at Kültepe and the Sarıkaya Palace at Acemhöyük (arrows indicate approximate minimum use periods based on dendrochronologically dated repairs/additions^18^) from the dendro-^14^C wiggle-match (Fig. 1b). These are compared with historical associations expressed in terms of Revised Eponym List (REL) dates from text records, placed according to the five main rival Mesopotamian chronologies^18–21^.

The MBA wiggle-match in Fig. 1b places likely earliest use (RY673) of the Waršama Palace at Kültepe^18^ ~ 1,837 to 1,826 BCE (95.4% hpd) and the earliest use (RY732) of the Sarıkaya Palace at Acemhöyük^18^ ~ 1,778 to 1,767 BCE (95.4% hpd). (The alternative reduced dataset 95.4% hpd ranges are almost the same: 1,835–1,826 BCE and 1,776–1,767 BCE.) A rich set of historical associations linked with the Old Assyrian Revised Eponym List (REL) should fit as respectively before, around, and following these dates^18,19,21^ (Fig. 2). For example, the lower town Kültepe Ib period is regarded as commencing around the start of the Waršama Palace and multiple documents link the Assyrian ruler Šamši-Adad I with both Kültepe Ib and the Sarıkaya Palace. The only Mesopotamian chronological schemes^20^ potentially compatible with the wiggle-match are the High Middle or (especially) Low Middle Chronologies (which are only 8 calendar years apart)^18,19,21,28–31^. Recent studies assessing the textual and astronomical data have also offered strong support for this solution^28–31^.The tree ring sequenced ^14^C placement and necessary set of relationships contradict the other candidates (Fig. 2). Our findings here, with additional data and the new IntCal20 calibration curve, confirm the resolution of Old Assyrian/Old Babylonian chronology around the Middle Chronology range and end a long-running debate.

### Egyptian New Kingdom radiocarbon time series versus IntCal20

**Figure 3:**
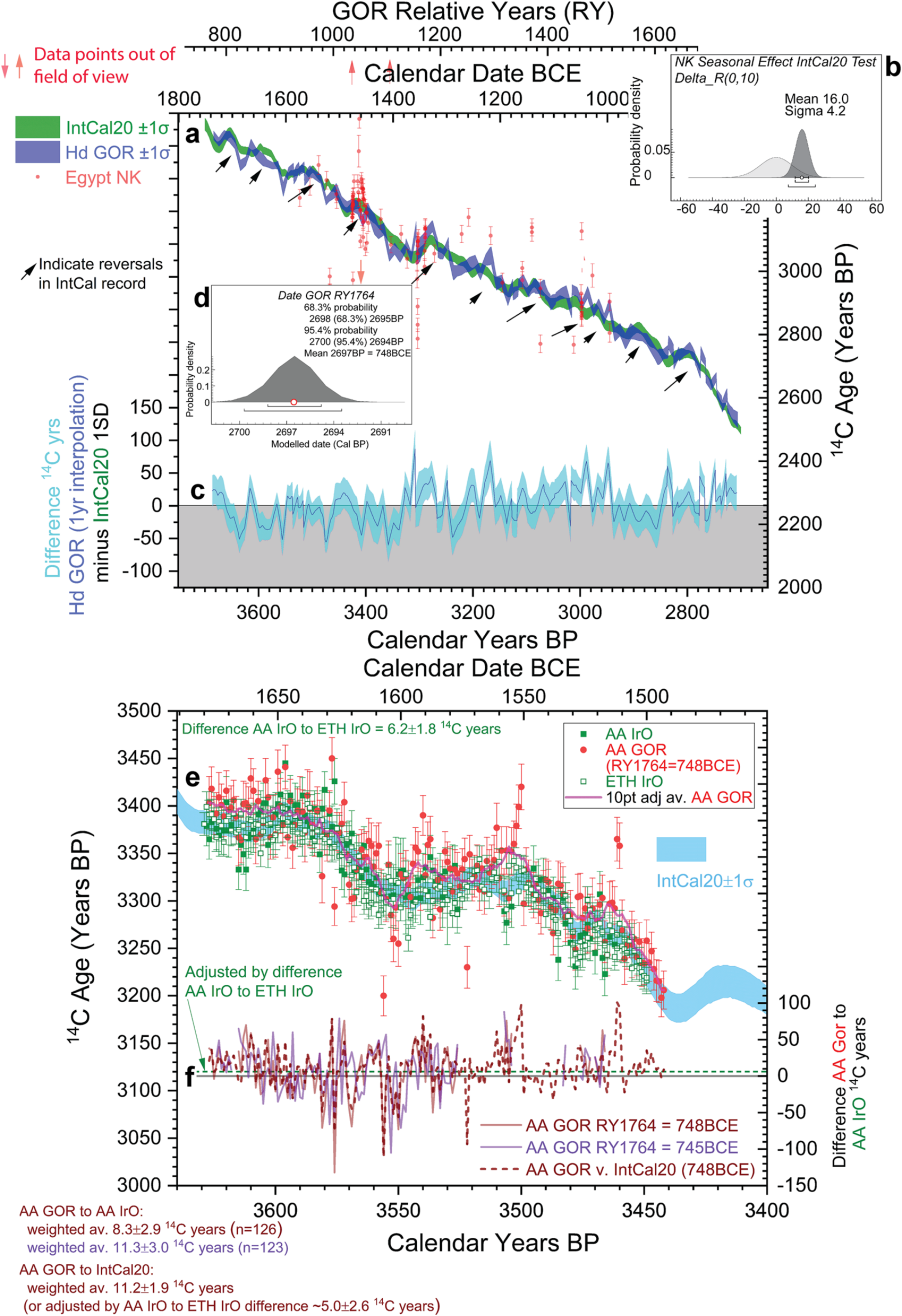
Comparisons of IntCal20^1^, Hd GOR^10^ and Egyptian NK^7^ datasets. (**a**) IntCal20 and Hd GOR records (± 1σ) and NK Egyptian time series. (**b**) Seasonal offset of the NK time series with IntCal20. (**c**) ^14^C offsets between Hd GOR and IntCal20 overall interpolated. (**d**) Posterior density placement of the GOR felling date RY1,764 versus IntCal20 using the Hd GOR data series minus outliers, placing the overall GOR chronology (RY737–1,764) ~ 3,724 to 2,697 Cal BP/1,775–748 BCE.** (e)** Comparisons of AA IrO, AA GOR (and 10 point adjacent average) and ETH IrO versus each other and IntCal20, **(f)** Differences AA GOR versus AA IrO and AA GOR versus IntCal20 according to placement of GOR chronology last ring (RY1,764).

The second ^14^C time series comprises the Egyptian New Kingdom (NK) dataset^7^. This indicated a seasonal ^14^C offset of ~ 19 ± 5 ^14^C years against IntCal04^7,32^. Re-run against IntCal20, the offset reduces slightly, but remains present at ~ 16 ± 4 ^14^C years (Fig. 3a,b, Supplementary Fig. S5). The revised Egyptian NK model with a neutral prior seasonal offset test of 0 ± 10 ^14^C years (Fig. 3b), or models running with a ΔR of 16 ± 5 ^14^C years, produce modelled ages for the NK rulers with IntCal20 that vary only very slightly, downwards, compared with the ages determined previously^7^. However, there are indications that the ^14^C offset likely fluctuates. We find that an alternative NK model^33^ which employs some revised reign lengths and the plausible longest reigns for the 18th Dynasty (ultra-high model)^34–36^, and so has a slightly different placement of the constituent groups of ^14^C data versus the calibration curve, offers a different (and much smaller) ΔR of ~ 6 ± 6 ^14^C years (Supplementary Fig. S5). This better fit, and recent review of the historical and astronomical evidence, may favour a longer/higher NK Egyptian historical chronology^36^.

### Gordion tree ring chronology versus IntCal20

The third long time series comprises ^14^C measurements on a tree-ring chronology from the Midas Mound Tumulus at Gordion (GOR) in central Anatolia^10,37–39^. There are two versions: a LLGPC Heidelberg (Hd) series^5,10,37^ and a AMS ^14^C Arizona (AA) series^38,39^. Wiggle-matched versus IntCal20 (see “Methods”, Supplementary Discussion 2) (Fig. 3a,c,d), the Hd GOR series (GOR RY737–1,764, ~ 1775 to 748 BCE/3,724–2,697 Cal BP) has a weighted average offset of 2.3 ± 2.1 ^14^C years (n = 117), with periods of fluctuating offsets in each direction. As observed in other cases, the positive offsets correspond generally with periods around reversals and plateaus in the ^14^C calibration curve^8,10,13^. It is evident, for certain periods, and in particular when there is a marked positive Hd GOR to IntCal20 offset (e.g. ~ 1,360 to 1,330 BCE), that the Egyptian NK time series corresponds better with the Hd GOR data than IntCal20 (Fig. 3a). An exception is around 1,470 BCE. Here the few and decadal Hd GOR data do not pick up the wiggle and apparent larger offset exhibited by the Egyptian samples.

The AA GOR series is much shorter in overall length (186 years), but comprises annual resolution data^38,39^. Wiggle-matched against IntCal20, they are placed (μ ± σ) 1,678 ± 1 BCE (GOR RY 834) to 1,493 ± 1 BCE (GOR RY 1,019) (Fig. 3e, Supplementary Fig. S6, extrapolated the 95.4% range for GOR RY1,764 is 751–746 BCE, μ ± σ = 748 ± 1 BCE). This is identical with the Hd GOR fit in Fig. 3a,d. The publication advocated chronological positioning from a χ^2^ fit^38^. We consider two approaches^40,41^ against both the IntCal20 modelled curve^1^ and a weighted average^42^ of recently published Irish Oak (IrO) and bristlecone pine (BCP) datasets^38,39^ (see Supplementary Discussion 2). These find the best (minimum) fit for the last ring and felling date, GOR RY1,764, 749–747 BCE (Supplementary Fig. S7), very similar to the OxCal results (Fig. 3e, Supplementary Fig. S7). Agreement on the approximate absolute calendar placement of the GOR time series suggests a robust fit (and we use the ~ 748 BCE fit).

**Figure 4:**
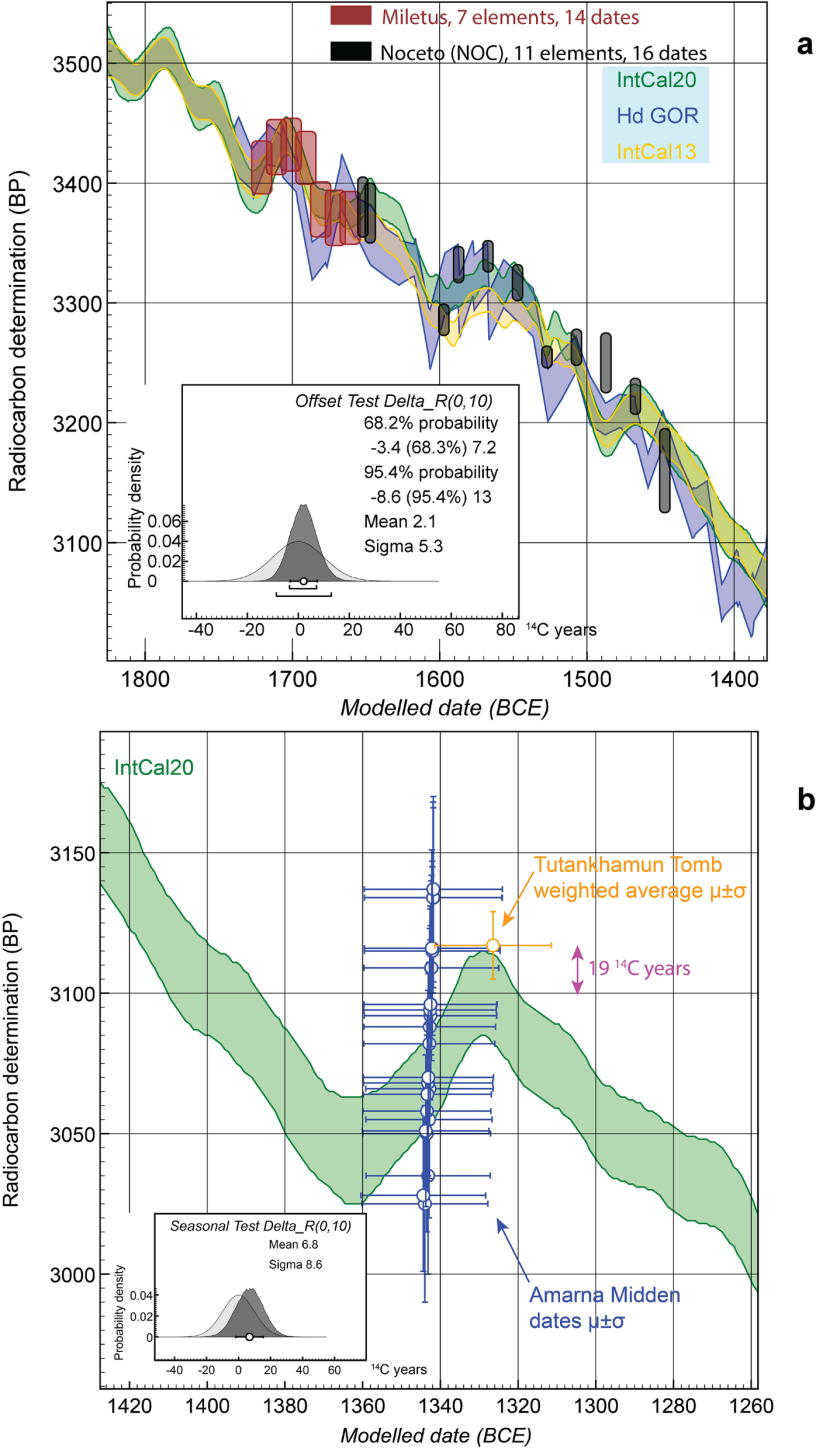
Instances of the differing ^14^C offsets between the Mediterranean-Near East and IntCal20 (± 1σ) at various periods. (**a**) The 1,700–1,500 BCE period, where IntCal20 is informed predominantly by many new AMS ^14^C dates^1,2^, shows little offset (contrary to the previous IntCal13^10,27^) and low elevation Mediterranean time series from Miletos, western Turkey, and Noceto (NOC), northern Italy, fit the calibration curve closely and show a negligible offset (the ~ 1,487 BCE NOC date may be an interesting exception, see text). Difference NOC versus IntCal20: 1.7 ± 6.1 ^14^C years; difference Miletos versus IntCal20: 1.2 ± 10.0 ^14^C years. Combined OxCal^24^ ΔR with neutral prior of 0 ± 10 ^14^C years gives μ ± σ of 2.1 ± 5.3 ^14^C years. (**b**) Small positive ^14^C offset during the Amarna period in Egypt contemporary with a reversal in the ^14^C calibration curve, especially at time of the death of Tutankhamun, when it reaches ~ 19 ^14^C years (but IntCal20 in this period is largely based on legacy ^14^C data—thus the offset observed may reduce once IntCal20 is updated with modern AMS ^14^C data for this interval).

However, there is a clear difference comparing the ^14^C ages from Hd GOR versus AA GOR versus IntCal20 (Fig. 3, Supplementary Fig. S8). In contrast to the Hd GOR time series, where the weighted average offset against IntCal20 is calculated as − 2.3 ± 2.1 ^14^C years (Supplementary Fig. S8), the AA GOR time series (over a much shorter period) and with considerable noise exhibits a much larger weighted average offset of 11.2 ± 1.9 ^14^C years (Fig. 3e,f). This tendency to an average positive offset is visible in Supplementary S6, where 69% of the AA GOR ^14^C data are older than the corresponding IntCal20 value. Latitude is suggested as an explanation^38,39^, but a previous ^14^C time series on Anatolian wood does not illustrate such systematically offset data^5^ (Supplementary Fig. S9). The Noceto (NOC) series from Italy also exhibits only a small average offset, as does the Miletos series from western Turkey, or data from Bcharre in Lebanon (Fig. 4a, Supplementary Fig. S9). Since measurements on the same IrO between AA and ETH indicate that AA is on average 6.2 ± 1.8 ^14^C years older^39^ (Fig. 3f), we might instead consider adjusting the AA GOR offset, perhaps by a similar amount (e.g. to ~ 5.0 ± 2.6 ^14^C years). This would then also be a typically small or negligible average offset (with variation, as evident from Hd GOR: Fig. 3c), and not far from the Hd GOR record (see below).

## Discussion

### Radiocarbon offsets and their causes

The three sets of comparisons indicate two key outcomes. First, across the second and early first millennia BCE, there is repeated evidence for the operation and effect of small offsets that impact the high-resolution dating of these Mediterranean-Near Eastern ^14^C datasets, even with the latest NH international ^14^C calibration curve (IntCal20). Second, such offsets are not constant, but appear to fluctuate over time. This suggests it would be misleading to apply a constant offset factor for individual dating cases that might, or might not, be relevant.

Evidently one key factor relevant to determining the nature and source of the offsets observed is the composition of the ^14^C calibration curve at particular periods. Much of the calibration curve record up until IntCal20 derives from laboratories using LLGPC or LSS^1,10,11,27,32^, and, except for the period ~ 1,700 to 1,500 BCE, most of the second to early first millennia BCE still does^1^. As noted, in several instances including this one, detailed new measurements of time intervals with AMS ^14^C have indicated slightly older ^14^C ages^1,2,10,38,39,43–48^. The MBA (87%) and Egyptian NK (100%) time series consist of AMS ^14^C dates. It is thus unclear how much of the scale of the observed ^14^C offsets may in fact be a difference between measurement techniques and technologies—versus an expected small but varying intra-annual seasonal ^14^C offset component^5–10^. For example, Mediterranean-Near Eastern ^14^C offsets within the period 1,600–1,900 CE observed comparing AMS ^14^C data with the previous LLGPC and LSS IntCal datasets^6,8^ remain, but are reduced, when compared with the new IntCal20 curve containing many new AMS ^14^C data for this period^1^. For example, the original Egyptian 18th–19th century CE average offset^6^ reduces from 19 ± 5 to 12 ± 5 ^14^C years (and the NK period offset may reduce with revisions to the historical intervals: see above), while the comparisons of the Oxford and AA Jordan juniper datasets^8^ similarly reduce from the reported average OxCal ΔR ^14^C year offsets of 19 ± 3 and 21 ± 5 to 12 ± 3 and 12 ± 5 ^14^C years. Egypt and the southern Levant represent almost maximally offset mid-latitude NH growth season timings versus central and northern Europe and North America^6,8,10^. This suggests the scale of a likely real average maximum seasonal offset factor, if the entire calibration curve comprised similar AMS ^14^C data, more of the order of ~ 12 ± 5 ^14^C years (~ 1 to 2‰). At about half the maximum intra-annual variation observed from atmospheric measurements^14^, this appears plausible. We accordingly revise previous estimates of typical seasonal ^14^C offsets^6,8,10^ downwards to this approximate range. In practice, the additional issue of inter-laboratory differences (see above), evident even among high-precision calibration laboratories, adds a further error component^1,10,27,32^ (Supplementary Discussion 1). Any average ^14^C offset in the Aegean–Anatolia region should be rather smaller, since the growing seasons are substantially less offset versus IntCal20 source trees^10^.

Two issues apply particularly to the 1,700–1,480 BCE interval (Supplementary Discussion 1). First, BCP tends to produce ^14^C ages older than contemporary IrO or IntCal20 by around 7–9 ^14^C years^39,49^. Second, AA ^14^C data overall for this period^38,39,43^ are older than the consensus (IntCal20) or in direct comparisons with ETH by around ~ 6 to 7 ^14^C years^39^. Thus the incorporation of several hundred AA BCP and IrO ages into IntCal20 1,700–1,480 BCE overly raises ^14^C ages in this section of the calibration curve. This AA-effect likely partly incorporates (or hides) any typical positive Mediterranean growing season offset, when relevant (Fig. 3c, Supplementary Figs. S6, S9)^10^. The Egyptian NK data support such a view. Ruling out two extreme outliers, it is noticeable that the 7 ^14^C elements of the Egyptian NK time series^7^ in the sixteenth century BCE are either around, or in fact below, IntCal20 (Supplementary Fig. S5).

For unknown reasons it is apparent that the Hd German Oak (GeO) data for the period ~ 1,660 to 1,540 BCE are too recent^1,43,48^. Despite good comparisons in other periods^10^ (Supplementary Fig. S9), there was a problem in this interval. The Hd GeO data 3,629–3,449 Cal BP (1,680–1,500 BCE) are − 15.6 ± 2.4 ^14^C years versus IntCal20, n = 57. But as noted, IntCal20 is a little old in this period. The Hd GeO series, when compared versus ETH IrO^39^ (weighted averages) for this period (common data available 3,625–3,431 Cal BP/1,676–1,482 BCE), are – 11.8 ± 2.8 ^14^C years, n = 49. In particular, Hd data on Knetzgau 40^10^ have been shown to be − 12.9 ± 3.1 ^14^C years more recent than measurements by three other laboratories on this tree^48^. Thus a previously observed offset between Hd GeO and Hd GOR in the earlier sixteenth century BCE^10^ is likely largely erased (Supplementary Discussion 1). Are the Hd GOR data similarly too recent? We argue no. As published, the Hd GOR data offer reasonable comparison with IntCal20, as would be anticipated given (1) the relevant growing seasons are not markedly offset (contrast Egypt or the southern Levant^8^), but with some periods of small offset when the difference was exaggerated^5,8,10^, and (2) the AA–IrO and especially BCP inflation of IntCal20 in this period likely already covers some to all of any typical Aegean–Anatolian offset. For example, were even the smaller of the offsets evident for the Hd GeO (just noted) also applied to Hd GOR, then there would be a large average offset, e.g. + 14.2 ± 2.8 ^14^C years versus IntCal20. But, as just discussed, revision and comparison of comparable datasets indicates maximum mid-latitude NH growing season offsets ~ 12 ± 5 ^14^C years, and the Gordion context should be substantially less offset. The same criticism of too large an average offset applies to the AA GOR data^38^. Since it is evident from a large set of parallel measurements of IrO by both AA and ETH (Fig. 3e) that the AA data are ~ 6.2 ± 2.8 ^14^C years older^39^, it seems likely these AA GOR data are on average too old also. If they were adjusted by around the ETH to AA IrO factor, as suggested above, then they too would offer a more plausible relationship with IntCal20.

### Radiocarbon offsets and Mediterranean chronology

The values for possible ^14^C offsets mentioned above are averages, and there will be variation around these (Fig. 3c,f)^5,8,10^. Such episodes could be important for high-resolution chronology. The historically well-dated Amarna period in Egypt offers a test case for a larger offset during the second millennium BCE, since it lies around the time of an apparent offset in Mediterranean ^14^C levels ~ 1,360 to 1,330 BCE from the Hd GOR dataset (Fig. 3a,c). A model combining the available ^14^C dates and the historical constraints^7, 33,50^ (Supplementary Table S3) indicates a maximum possible offset around the time of the burial of Tutankhamun of ~ 19 ^14^C years versus IntCal20 (Fig. 4b). However, since this part of IntCal20 comprises legacy data, we might anticipate this offset reducing a little in the future (compare our MBA case above).

**Figure 5:**
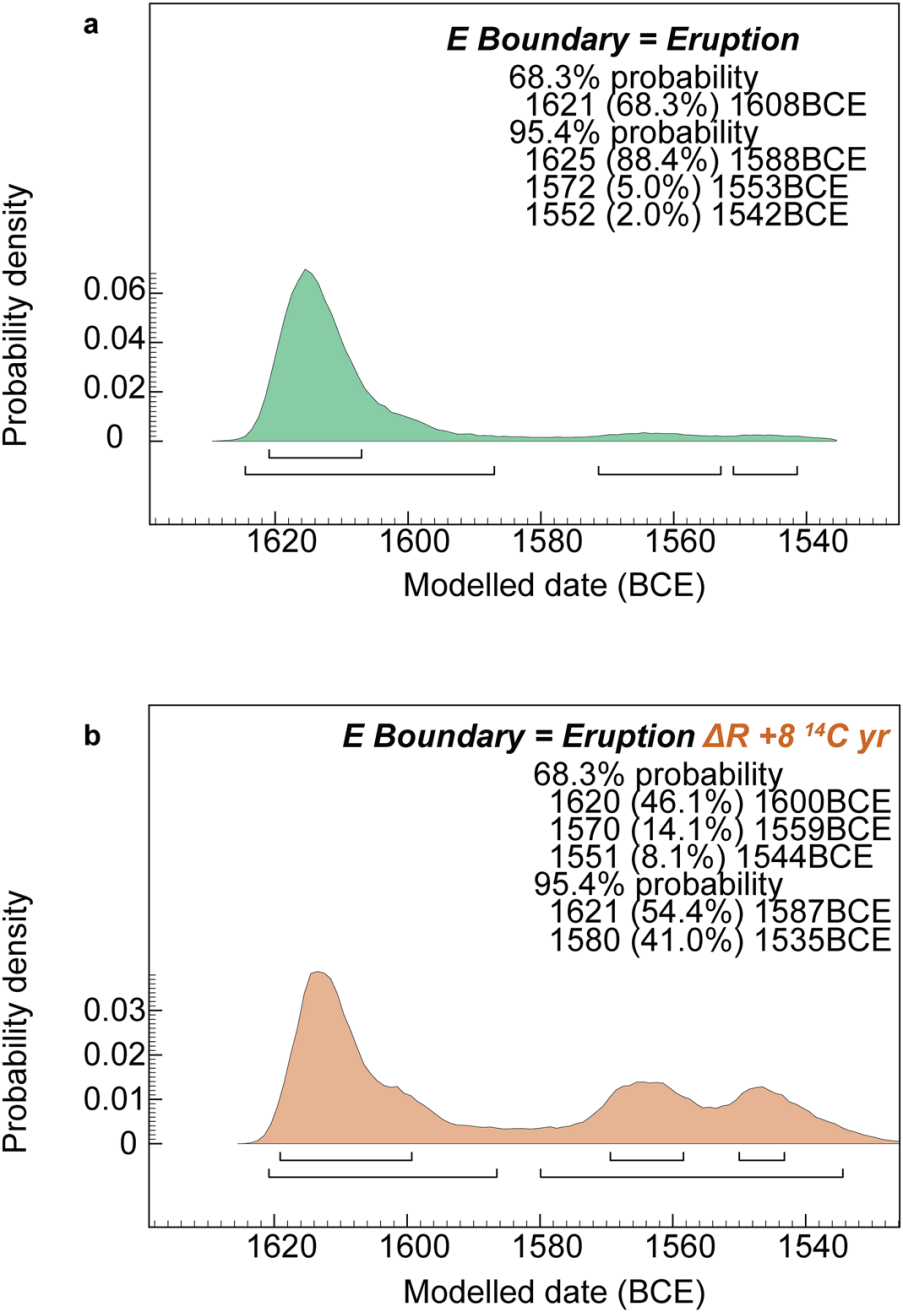
Calendar dating probabilities and ranges for the Santorini/Thera volcanic eruption following published dataset (with one subsequent addition: see Supplementary Discussion 3, Supplementary Table S3) and an appropriate method^52,53^. (**a**) With IntCal20^1^, resolution = 1 year, and assuming no substantive Mediterranean ^14^C offset at this time beyond that covered already by IntCal20 for this period (as indicated in Figs. 3a,c, 4a, Supplementary Fig. S9). (**b**) As (**a**), but applying a hypothetical positive Aegean-region ~ 8 ^14^C years offset (OxCal ΔR of + 8 ^14^Cyears) (Supplementary Discussion 1, Supplementary Fig. S6).

Even small changes in  ^14^C ages can make large calendar differences during reversals and plateaus in the calibration record. There is a narrow distinction between a late seventeenth and earlier-mid sixteenth century BCE date range with IntCal20. Yet this determines the much-debated date of the Thera/Santorini volcanic eruption^2,10,33,38,43,47,48,51–55^ (Supplementary Discussion 3). Analysis with IntCal20 using (1) weighted average ^14^C ages^2^, (2) a published dataset and alternative appropriate method^52,53^, or (3) the series of ^14^C dates on an olive branch found buried by the Santorini/Thera eruption^2,53,55^, all indicate a most likely late seventeenth century BCE date, but include varying probability in the earlier-mid sixteenth century BCE (Supplementary Figs. S10a,b, S11a,b, Fig. 5a). However, if the eruption was coeval with a small positive offset—for example of up to ~ 8 ^14^C years (1‰) (see above, Supplementary Discussions 1, 3, Supplementary Fig. S6)—this moves substantial or majority probability from the later 17th to the earlier-mid sixteenth centuries BCE in (1) and (3) (Supplementary Figs. S10c,d, S11c, Fig. 5b).

**Figure 6:**
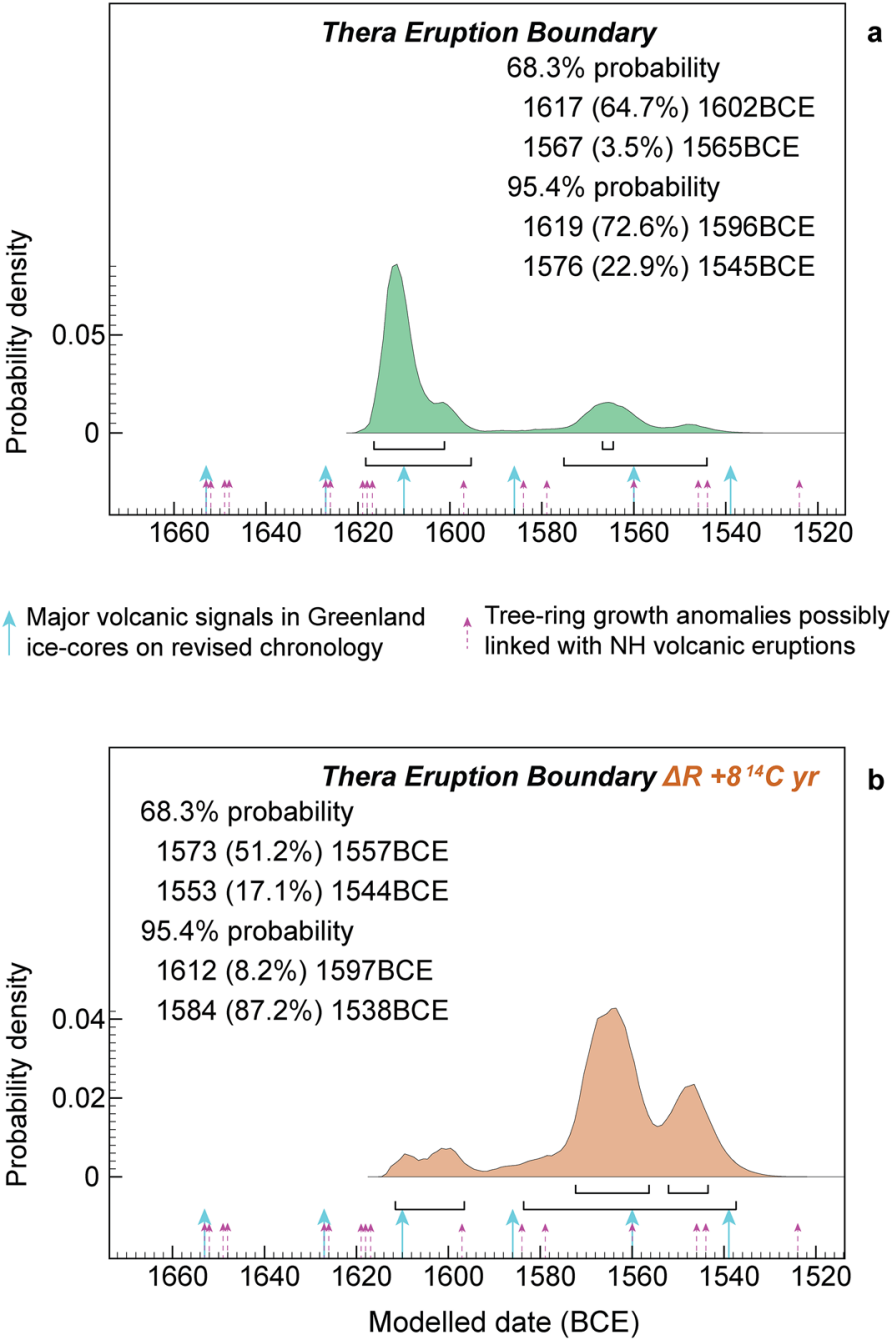
Modelled dating probabilities for the Thera eruption from the southern Aegean model (Supplementary Fig. S12). (**a**) Modelled Thera eruption boundary (age estimate) including ^14^C data from Thera—median A_model_ result from 11 runs (Supplementary Fig. S13). Arrows indicate major volcanic signals in recently re-dated Greenland ice-core records^67^, along with some published tree-ring growth anomalies suggested potentially to be associated with major volcanic eruptions^38,67–69^ (see Supplementary Discussion 3). (**b**) As (**a**) but applying a hypothetical additional positive Aegean-region ~ 8 ^14^C years offset (OxCal ΔR of + 8 ^14^C years) (Supplementary Discussion 1, Supplementary Fig. S6). Of the definite volcanic signals represented in the Greenland ice, either (higher option) 1,610 BCE, or (lower option) 1,560 BCE appear respectively plausible and most likely. OxCal^22–24^ models in Supplementary Table S3 and described in Supplementary Discussion 3, using IntCal20^1^, with resolution = 1 year.

In the Thera case, it was suggested recently that “to gain more precise insight into the timing using ^14^C, modelling of multiple ^14^C dates will likely be needed”^2^. We revise and up-date a Bayesian model^51^ (see Supplementary Discussion 3, Supplementary Table S3, Fig. S12) incorporating 147 ^14^C dates and archaeological information from Thera and the southern Aegean for the periods before, contemporary with, and after the Thera eruption. The modelled dating probability for the Thera eruption, using the median OxCal A_model_ result from 11 model runs (Supplementary Fig. S13) is shown in Fig. 6a. Across the 11 runs the total dating window at 95.4% hpd is 1,619–1,543 BCE and the most likely 68.3% hpd regions overall are ~ 1,617 to 1,601 BCE (average 62.8% hpd) and ~ 1,570 to 1,562 BCE (average 5.4% hpd) (Supplementary Fig. S13). Did any additional ^14^C offset apply beyond that already incorporated in IntCal20 (see above)? If, for example, even an 8 ^14^C year offset applied, then the dating probability in the Fig. 6a model largely switches to the earlier-mid sixteenth century BCE (Fig. 6b). Contrary to previous advertisements^43^, a date for the Thera eruption after ~ 1,543/1,538 BCE remains improbable (end 95.4% hpd, multiple runs Fig. 6 models), ruling out the conventional ‘low’ chronology range ~ 1,530 to 1,500 BCE^33,52^, but final placement depends on clarification of the reality (or not) of a small additional positive ^14^C offset. While, at first glance, this is perhaps suggested by the AA GOR data (Fig. 3, Supplementary Fig. S6), it is contradicted by other available data (see above), and is likely not supported even by the AA GOR data series once the evident inter-laboratory offset and excessive noise is removed (Supplementary Fig. S14, Supplementary Discussion 1). The better fit of a longer/higher Egyptian NK chronology versus IntCal20 noted above (Supplementary Fig. S5b) is potentially important. Such revision brings the time range of the Thera eruption (either Fig. 6a or b) much closer to the start of the New Kingdom. This could minimize a time difference previously viewed as problematic^52^, and might start to permit discussion of suggested possible associations between these episodes^56^.

Thera is a well-known case, but there are many other instances of high-resolution ^14^C chronologies key to Mediterranean and Near Eastern pre- and proto-history^7,8,18,21,33,57–63^. Our examples highlight the need to determine a high-resolution Mediterranean-Near Eastern ^14^C record in order to clarify the question of fluctuating small offsets as relevant to regional ^14^C levels over time. At present, a basic problem is that comparisons for many periods (where extensive new annual resolution AMS ^14^C data are not yet available) merge two separate issues: (1) differences between older LLGPC and LSS ^14^C calibration data versus newer AMS ^14^C data, as well as (2) an apparent modest seasonal ^14^C component. Any general approximation is an unsatisfactory solution since offsets appear to vary over time (likely associated with varying ^14^C production, climate and plant physiology processes^5,8–10,15^). Especially at times of reversals and plateaus in the ^14^C calibration curve, even modest variations may have great import for high-resolution chronology in the Mediterranean and Near East, and could affect a number of long-running debates. For those periods of IntCal20 still primarily based on LLGPC and LSS data, we have shown that such offsets affect accurate high-resolution chronology using AMS ^14^C dates. Resolution requires deconvolution of the now mixed IntCal record. Ideally, AMS ^14^C dates should be calibrated against an AMS ^14^C derived calibration record, and LLGPC and LSS dates against a LLGPC and LSS derived ^14^C calibration curve. Remaining offsets and variations would then have other causes, such as seasonal effects.

## Methods

### Radiocarbon dates and samples

^14^C dates employed combine sets of dates previously published with methods and full information^7,10,18,37–39,43,50,51,55^ (Supplementary Discussion 3) and sets of new dates run at the Eidgenössische Technische Hochschule (ETH) Zürich ^14^C laboratory (Supplementary Tables S1, S2). The dendrochronology of the *Juniperus* sp. time series from Acemhöyük, Karahöyük and Kültepe is published^18^. Timbers from the Waršama Palace at Kültepe include bark. Hence the felling date and likely primary construction 0–1 year later is placed RY670–672, and so suggest earliest building use likely ~ RY673^18^. Timbers from the Sarıkaya Palace at Acemhöyük include bark (felling date and likely primary construction 0–1 year later) at RY730–731, and so suggest earliest building use likely ~ RY732^18^. The ^14^C dates and dendrochronology of the Gordion time series employed is published^5,10,37–39,64^. The Erstein (ERST) ^14^C measurements are on oak (*Quercus* sp.) samples from a tree-ring chronology built from preserved timbers recovered as part of archaeological excavations undertaken before the development of the Parc d’Activités du Pays d’Erstein, Erstein, France (48.4269N, 7.6386E)^25,26^. Dendrochronological crossdating places the sample used, ERST 5964-GBS-218-37, at 2,010-1,764 BCE. The Egyptian NK data and the OxCal CQL2 code have been published^7^. The revision of this OxCal model, adjusted to incorporate subsequent studies on historical Egyptian chronology and the reign lengths of kings^34–36^ using the ultra-high version for the earlier NK^34^ is also published^33^. Details on the Miletos and Noceto tree-ring samples and ^14^C dates are published^10,51^. Where plotted in the figures, ^14^C dates (or weighted averages) are shown with 1σ errors (Y axis). In Figs. 1a–c, 4a and Supplementary Fig. S3 the X axis width of the plotted date, or weighted average age, indicates the 68.3% hpd wiggle-match range. In Fig. 4b and Supplementary Fig. S5 the ^14^C dates or weighted averages show the ^14^C value (age or mean) on the Y axis and the mean ± σ values of the modelled posterior density distributions on the X axis. Pretreatment and processing of samples and their AMS ^14^C dating at the ETH laboratory followed methods previously described for similar wood/charcoal samples^4,44,65,66^.

### Radiocarbon modelling

We employed OxCal^22–24^ using versions 4.1.7, 4.3.2 and 4.4.1 with the IntCal20 NH ^14^C calibration curve^1^ (curve resolution set at 1 year). Where ^14^C dates comprised the same (cross-dated) tree-rings or mid-points, and so represent estimates of (approximately) the same ^14^C date/calendar age relationship, we combined these into weighted averages^42^ using the R_Combine function in OxCal. Where sets of tree-rings comprise the sample we regard the date as the mid-point (e.g. for Relative Years, RY, 1–5 this would be RY3). Where a sample comprised an even number of tree-rings, e.g. RY1–10, then the mid-point is treated as RY5.5 (after RY5 and before RY6). Where applicable, individual outliers were identified and down-weighted using the OxCal SSimple Outlier model^23^. The SSimple Outlier model was also used to assess weighted averages against the model. The tree-ring time series were analyzed (‘wiggle-matched’) using the D_Sequence function of OxCal^22^.

The MBA time series comprises 76 ^14^C dates. After combining dates with the same mid-points the time series contains 40 elements. However, three of the weighted averages fail a χ^2^ test for representing the same age (mid-points RY651, 659 and 691)^42^. In each case the OxCal SSimple Outlier model identifies one date as the clear outlier and so we removed three dates: ETH-78942.1.1 (outlier probability ~ 53%), OxA-29963 (outlier probability ~ 65%) and ETH-78947.1.1 (outlier probability ~ 84%) (see Supplementary Table S3). One other date (OxA-30907) had a large offset between the δ^13^C value measured by the AMS versus the stable isotope MS (suggesting fractionation at the level of 1.1%). Sometimes this indicates an issue with a sample and an unexplained age offset, making this sample and date suspect. We thus excluded it on this ground—the date was also an outlier at ~ 20% probability. The remaining time series contains 72 dates and 39 elements. The OxCal runfile is in Supplementary Table S3. The dataset does not provide a good visual fit with the calibration curve (Fig. 1a)—many data are placed below or away from the calibration curve—failing an overall χ^2^ test (T = 65.4 > 52.6 df38 at 5%) and delivering poor OxCal Agreement indices (A_comb_ = 10.3 < A_n_ = 11.3%, A_model_ and A_overall_ ≤ 10, well below the satisfactory value of 60). It appears likely there is a systematic offset between the data measured and the calibration curve. To investigate we used the Delta_R (ΔR) function in OxCal^24^. This allows investigation of whether a data set exhibits a systematic shift relative to the calibration curve. We employed a neutral prior ΔR value of 0 ± 10 ^14^C years. For a number of model runs Convergence values are poor (< 95). The reason is that the ΔR model in these cases produces a bi-modal result. The possible offsets are on average (usually more likely) ~ 22 ± 5 ^14^C years or the very different − 32 ± 8 ^14^C years. Only in some runs did the model converge successfully (all elements with Convergence, C, values ≥ 95) and in these cases usually a single ΔR range of ~ 22 ± 4 ^14^C years was found and occasionally the alternative -32 ± 8 ^14^C years range (substantially increasing the kIterations value, and so run time, usually resolved the low C values, but retained the ambiguity). The ΔR posterior densities from ten example runs (six bi-modal, three with about a 22 ^14^C years offset, and one with a − 32 ^14^C years offset) are illustrated in Supplementary Fig. S1. It is evident there is an offset. We tried models with a ΔR of 22 ± 5 ^14^C years, which appears the likely solution based on the model runs for Supplementary Fig. S1. We also tried runs with the alternative ΔR − 32 ± 8 ^14^C years. The ΔR of 22 ± 5 ^14^C years yields a satisfactory visual solution (Fig. 1b). Without consideration of any further outliers, the OxCal diagnostic values, A_model_ and A_overall_ are typically ≥ 60 (~ 60 and ~ 65 respectively). At this point there is then one major outlier date, OxA-30908, with an outlier probability of ~ 64% (no other outlier probability is above ~ 25/26%, and in all only 7 values are ≥ 10% from multiple runs). If we exclude OxA-30908 and re-run the model, the placement is identical and the A_model_ and A_overall_ values exceed the satisfactory threshold value of 60 at ~ 76 and ~ 80. Thus we use the fit and placement shown in Fig. 1b. The ΔR posterior density offers good agreement with the prior of 22 ± 5 ^14^C years (Fig. 1c). In particular, the set of ACM values offer a good and specific fit around the wiggle in the calibration curve ~ 1,850 to 1,810 BCE (contrary the notably poor fit in Fig. 1a with the earlier placement). In contrast, model runs with the alternative (earlier) fit with a ΔR of − 32 ± 8 ^14^C years achieve unsatisfactory OxCal A_model_ and A_overall_ values, all < 30, well below the satisfactory threshold value of 60. The ΔR posterior density also offers poor OxCal agreement values (< 60) with the prior. The visual fit is poor with most data not matching the calibration curve, and instead placed below the curve (Supplementary Fig. S2). Thus we exclude this fit range as viable. (We note that the older alternative option, about 81 calendar years earlier than the fit shown in Fig. 1b, is in fact likely too early to correspond with the High Mesopotamian Chronology^20^, which is only ~ 56 years earlier than the Middle Chronology. Even at the limits of 68.3% hpd and 95.4% hpd, the difference is at least 72 and 62 calendar years respectively, leaving any correspondence as unlikely. Moreover, regardless, the older solution is clearly unlikely on the basis of the ^14^C wiggle-match data just discussed. This instead offers a good correspondence only with the High Middle Chronology or Low Middle Chronology, see text and Fig. 2.)

The Egyptian NK models are used as published^7,33,50^. The wiggle-match calendar placement of the Hd GOR time series^22^ uses the placement with satisfactory OxCal agreement indices after removing the 13 or 14 largest outliers (SSimple outlier model^24^ applied to individual dates, dates in weighted averages, and the weighted averages^42^). The minimum almost satisfactory case removes 13 individual outliers and achieves Amodel ~ 58 and Aoverall ~ 61, while removing 14 individual outliers achieves Amodel ~ 72 and Aoverall ~ 74 (dates removed are indicated in the OxCal runfile in Supplementary Table S3). This places the last year of the chronology RY1,764, with bark (felling date) ~ 748BCE (Fig. 3d). This fit is 2 years later than the OxCal best fit using all data (against IntCal20 or IntCal04^10^) but with poor OxCal agreement indices. The OxCal wiggle-match of the AA GOR dataset uses IntCal20 with no outlier model following the publication^38^ (Supplementary Fig. S6). The χ^2^ least squares and χ^2^ fitting of the AA GOR data uses published methodologies^40,41^ (see Supplementary Discussion 2, Supplementary Fig. S7).

Comparisons of ^14^C datasets were made using the quoted data, or via 1-year linear interpolations of the multi-year Hd GOR and GeO datasets (e.g. Fig. 3a,c, Supplementary Fig. S6). Weighted average^42^ comparisons are cited for the relevant pairs of data, 10-year block mid-points were rounded by 0.5 years.

The Miletos and Noceto wiggle-match data were used as published^10,51^. For details on the Thera/Santorini case and the data analysis, see Supplementary Discussion 3 and Supplementary Table S3. Since it has been suggested in the past that ^14^C dates on samples from Thera could have been affected by volcanic CO_2_ (despite no positive evidence as regards any archaeological sample)^33,51,53,55^, we also consider models excluding all ^14^C data from Thera (Supplementary Fig. S15). These offer similar but slightly less constrained results.

The OxCal CQL2 runfiles, with annotations indicating outliers not used and some other details, are provided in Supplementary Table S3. It should be noted that each run of such Bayesian models is different and small variations occur. In well-constrained data sets where there is a single best fit location or Sequence solution, these tend to be small and in the range of, e.g., 0–2 years. It is important to observe that—except where noted (6 cases in Supplementary Fig. S1)—we only employed data where the model run achieved satisfactory Convergence, C, values ≥ 95. We report typical examples from multiple model runs.

### Historical and archaeological associations

The archaeological associations between the contexts of the MBA tree ring time series and the sites of Kültepe and Acemhöyük are as previously outlined^18,19,21^. The construction of the Egyptian NK model and the historical priors included are as published^7,33^. The Amarna model is explained in Supplementary Table S3. The Aegean model, revising a previous model^51^, is explained in Supplementary Discussion 3 and in Supplementary Table S3.

## Supplementary information

Supplementary information.

## Data Availability

All data generated or analyzed during this study are included in this published article (and its Supplementary Information files), or are previously published. The newly published raw ^14^C determinations are in Supplementary Tables S1 and S2. All other ^14^C dates have previously been published and are available from the relevant publications^7,10,18,37–40,43,50,51,55^ (and see Supplementary Discussion 3 and Table S3). The IntCal20 dataset^1^ is available from https://intcal.org/.
