# Supplementary material for: Radiocarbon offsets and old world chronology as relevant to Mesopotamia, Egypt, Anatolia and Thera (Santorini)
Source: Sci Rep. 2020 Aug 17;10:13785. doi: 10.1038/s41598-020-69287-2 (PMC7431540; doi:10.1038/s41598-020-69287-2)
Supplement: Supplementary file 1 — Supplementary information. [file 41598_2020_69287_MOESM1_ESM.docx]

**Radiocarbon offsets and old world chronology as relevant to Mesopotamia, Egypt, Anatolia and Thera (Santorini)**

Sturt W. Manning^1*^, Lukas Wacker^2^, Ulf Büntgen^3,4,5,6^, Christopher Bronk Ramsey^7^, Michael W. Dee^8^, Bernd Kromer^9^, Brita Lorentzen^1^, Willy Tegel^10,11^

^1^Cornell Tree Ring Laboratory, Department of Classics, Cornell University, Ithaca 14853, NY, USA

^2^Laboratory for Ion Beam Physics, Swiss Federal Institute of Technology in Zurich, CH-8093 Zürich, Switzerland

^3^Department of Geography, University of Cambridge, Cambridge CB2 3EN, UK

^4^Swiss Federal Research Institute WSL, CH-8903 Birmensdorf, Switzerland

^5^Global Change Research Institute CAS, 603 00 Brno, Czech Republic

^6^Department of Geography, Masaryk University, 611 37 Brno, Czech Republic

^7^Research Laboratory for Archaeology, School of Archaeology, University of Oxford, Oxford OX1 3TG, UK

^8^Centre for Isotope Research, Faculty of Science and Engineering, University of Groningen, Nijenborgh 6, NL-9747 AG Groningen, Netherlands

^9^Institute of Environmental Physics, University of Heidelberg, D-69120 Heidelberg, Germany

^10^Chair of Forest Growth and Dendroecology, Institute of Forest Sciences, University of Freiburg, Freiburg, Germany

^11^Archaeological Service Kanton Thurgau (AATG), 8510 Frauenfeld, Switzerland

**Supplementary Information**

Supplementary Discussions 2

1. Inter-laboratory ^14^C Offsets 2
2. Wiggle-match placement of the Gordion tree-ring series 9
3. History and context of the Thera/Santorini dating debate 13

*Introduction* 13

*The eruption* 14

*Relative dating* 16

*Absolute dating* 17

*^14^C dating the Thera eruption 2020* 18

*Discussion* 24

Supplementary Figs. S1 – S17 29

Supplementary Tables S1 – S4 54

Supplementary References [S1-S198] 119

**Supplementary Discussion**

***1. Inter-laboratory offsets***

It is important to begin by highlighting and reminding readers that even the best ^14^C laboratories exhibit variations in measurements against other laboratories. Some variation is to be expected in any such imperfect measurement estimation process. The key issue for high-precision ^14^C dating and calibration is evidence for systematic offsets—versus random noise—such that one laboratory systematically tends to produce ^14^C age values that are older or more recent than another laboratory, or older or more recent than those consensus values which comprise the ^14^C calibration curve. The issue of inter-laboratory offsets, and the need to investigate/control for these, has been around for decades [refs. 2, 27, 32, 39, 44–49, S1–S3], and comparisons were given in some of the publications accompanying iterations of the IntCal calibration curve [refs. 27, 32, S4]. Examination of the various intercomparisons illustrates how such variations affect ^14^C dates on dendrochronologically dated wood [ref. S5]—primarily illustrating an underestimation of real errors. A corollary, since it is almost impossible to avoid some inter-laboratory variations, is that if one wishes, for example, to investigate the issue of a growing season offset, then the data ideally should be measured from the two loci being compared at the same laboratory to rule out inter-laboratory variations as a complicating element [refs. 5, 10, 38, 39, 43].

The identification and consideration of inter-laboratory offsets is particularly important when data are included in the IntCal dataset, or used to suggest calibrated calendar ages. If a laboratory tends to produce ^14^C values that are, for example, older than the consensus (e.g. IntCal), then this will seem to produce more recent calendar ages than likely correct, and the reverse. The most valuable measure is direct comparison of ages measured on the same sample material by different laboratories. This is the model of the various Glasgow-organized inter-comparisons [refs. S1, S3, S5, S6].

Data produced at the Arizona radiocarbon laboratory (AA) identified a need to revise the IntCal record in the period ~1660–1540 BCE based on measurements of known-age North American bristlecone pine (*Pinus longaeva* D.K. Bailey) (BCP) and Irish Oak (IrO) [ref. 43]. The finding led to further work and analysis both by Arizona and other laboratories [refs. 10, 38, 39, 48, 49]. This confirms that IntCal needed revision in this period (which is now incorporated in IntCal20 [ref. 1]). However, at the same time, comparisons of datasets produced across this interval alert us to apparent differences—inter-laboratory offsets.

AA and the Eidgenössische Technische Hochschule Zürich ^14^C laboratory (ETH) measured samples of the same IrO [ref. 39]. This found found an average offset, with AA older, of 6.2±1.8 ^14^C years [ref. 38] (combining two comparisons on IrO yielding difference of AA being older than ETH by 5.8±2.3 ^14^C years, n=127, and 6.8±2.9 ^14^C years, n=87). A comparison of the same AA measurements on IrO versus IntCal20 finds AA older by 5.8±2.7 ^14^C years (weighted average, following [ref. 42]). Thus we may conclude that the AA data (measured at this time and for this interval, at least) are a little older than the ‘crowd-sourced’ IntCal20 values and specifically ETH values. ETH is a major contributor of new AMS ^14^C data to IntCal20. The IntCal20 comparison with AA is not independent of AA, since the hundreds of AA data on IrO and BCP are included in IntCal20 in a period of interest for this study, 1700–1480 BCE.

Let us consider the BCP. The original AA data on BCP and IrO shows that the weighted average of the AA BCP versus AA IrO was older by ~6±3 ^14^C years (note: correcting a typo in ref. [10]). Ref. [48] likewise notes a 5±3 ^14^C years offset (BCP older) between their GeO and the ref. [43] BCP between 1625–1510 BCE. A subsequent study comparing additional AA IrO data found the offset was slightly larger: 8.8±2.7 ^14^C years [ref. 39]. This is very similar to a comparison of the AA BCP versus IntCal20 where the weighted offset is 8.9±1.6 ^14^C years. We may further compare data on BCP from another laboratory from the earlier 1^st^ millennium BCE. A study comparing annual BCP and German Oak (GeO) samples measured at the University of California Irvine AMS facility (UCIAMS) [ref. 50], depending on a possible year 0 error in the known ages of the BCP, gives weighted average offsets of BCP to IntCal20 of 7.4±3.5 or 7.2±3.6 ^14^C years. In contrast, the data for the UCIAMS GeO for (i) Early Wood gives a weighted average offset versus IntCal20 of 0.5±1.1 ^14^C years and for (ii) whole years -2.4±2.0 ^14^C years. Hence, from both the AA and UCIAMS datasets, BCP appears around 7 to 9 ^14^C years older from both within-laboratory comparisons and from comparison with the IntCal20 modelled consensus. And: the IntCal20 consensus includes both the AA BCP measurements and AA IrO—hence, if these were excluded, the BCP offset versus IntCal would be slightly larger in the 1700–1480 BCE (3649–3429 Cal BP) period. The weighted average difference between ETH IrO and the AA BCP supports this assessment of a slightly larger real offset. The difference across 186 pairs of measurements is reported at 13.6±1.8 ^14^C years [ref. 39].

The net conclusions are that (i) AA ^14^C data at least for the period 1700–1480 BCE are slightly offset overall to older ^14^C values, and (ii) BCP data are offset to older ^14^C values, and hence AA BCP data are older for both (combined) reasons. This is important because there are indications from other evidence that Mediterranean ^14^C values can exhibit offsets because of differences in growing seasons (considering cases of positive offsets [ref. 10]) of perhaps around a similar net order of magnitude—even excluding the additional issue of AMS ^14^C versus previous technologies ([ref. 10], and see main text). Thus the inclusion of the AA datasets in IntCal20 for the period 1700–1480 BCE has the effect of partly ‘Mediterranean-ising’, IntCal20 for this specific interval for periods when there might be a positive Mediterranean ^14^C offset since it already includes a portion of the likely range of any possible Mediterranean offset (see further below). But the corollary is that in times when there is no Mediterranean offset, or in fact a negative offset (Fig. 3, [ref. 10]), then IntCal20 in this period is to some extent too old because of these AA data, and likely yields slightly too recent calendar age estimates. This is a reverse circumstance compared with IntCal13 where—now it is realized that this was too low in the parts of the 17^th^ and 16^th^ centuries BCE—dates were slightly too old once calibrated. We have thus exchanged one problem for another.

The origin and nature of the BCP offset is unclear. An offset of ~7–9 ^14^C is observed in both the AA and UCIAMS studies discussed above. These represent similar observations at two different time periods. Much more limited previous studies have also indicated older ages for bristlecone pine for the specific period investigated [ref. 16]. However, in some other comparisons no such offset is evident. For example, measuring 11-year blocks of BCP a few years earlier covering 795-355 BCE, the UCIAMS laboratory data (excluding 685 BCE where the two dates for this sample fail a χ^2^ test – T = 6.7 > 3.8) return a weighted average difference of -5.2±3.1 ^14^C years versus IntCal20 [ref. S7]. Measurements at ETH on BCP for the periods around the two Miyake events 770-780 CE and 990-1000 CE found BCP values very close to the average from all their Northern Hemisphere values (all samples prepared and measured at ETH) [ref. 4]. Thus it is unclear whether BCP is often, or only sometimes, offset to older ages (and where there is no same-laboratory comparison, much depends on the source of comparison data in IntCal20).

The growing season for BCP, while short, largely matches other central-northern European trees or North American trees used for IntCal. BCP grows from mid-late June through late July to early August—though photosynthesis continues into the autumn [refs. S8, S9]. Where an offset to older ^14^C ages for BCP is observed, as in the 17^th^-16^th^ centuries BCE, latitude has been proposed as the cause [refs. 38, 39], but, as just noted, the findings for the two Miyake event periods do not support this, since BCP produced values very close to the Northern Hemisphere average [ref. 4]. Moreover, latitude is largely a constant for the BCP (since the BCP growth zone is well beyond the range of dynamic changes associated with shifts in the position of the ITCZ), thus the existence of some periods where there is a positive offset, but other periods when there does not seem to be such an offset, does not appear easily or consistently explained by mere latitude.

In the past, the extreme altitude of the growth environment of BCP samples has been suggested or considered as a relevant factor [refs. 1, S10, S11], but there have not been further investigations of this topic as regards ^14^C in recent years. Differences in δ^13^C are noted between lower and higher elevation BCP [ref. S12], reflecting different growth constraints between these contexts [ref. S13]. The very resinous-nature of BCP (at the more extreme end of conifer samples – long noted as requiring more aggressive pretreatments to holo-cellulose or α-cellulose – compared to more standard situations in order to remove extraneous material [refs. S14–S16]) is potentially another issue. The relevant AA samples in the above cases (including the BCP) have all been stated as being processed to “bright white holo-celullose” [ref. 39]. The ETH samples received a similar but more extended pretreatment [refs. 66, 67]. The UCIAMS samples including BCP were prepared with ABA only [ref. 50]. No problem is evident. The apparent scale and direction of offset is similar for Anatolian juniper and the BCP in the AA measurements [refs. 38, 39]—leading to the latitude suggestion as a common explanation —but, apart from the points noted above, this then ignores (i) the obvious differences in typical growing seasons between the Anatolian juniper and the bristlecone pine [ref. 10] which, logically, should reflect some part of the known seasonal cycle in atmospheric ^14^C levels [ref. 14] and hence differ, and (ii) the indications that the GOR offset as measured by both AA and Hd varies over time (Fig. 3). A latitudinal offset should be relatively consistent, whereas a growing season offset may vary substantially over both the shorter or longer term. This not only reflects varying climate but also plant physiology—a number of Mediterranean species exhibit plasticity and thus allow climate and growth environment to modulate the boundaries of their growing season from year to year [refs. 10, 15]. In support of this assessment, a long CE period Anatolian ^14^C time series indicates no systematic offset and good agreement with IntCal (Supplemental Fig. S9, [refs. 5, 10]). This series is particularly relevant because they are higher elevation, and thus likely do not exhibit the typical lower elevation Mediterranean growing season offset [ref. 10]. But, if latitude was the determinant, then they should clearly illustrate such an effect as a higher elevation dataset. They do not. The only period of minor offset within this series correlates with a major solar/climate episode [ref. 5]. Comparison of the Hd measurements on contemporary GeO and GOR samples found a varying offset, sometimes positive, sometimes around zero, and sometimes negative [ref. 10] (Figs. 3a, c and Supplemental Fig. S9) – this is inconsistent with a latitude-based explanation which should offer a consistent offset. Instead, it appears to reflect the temporally varying impact of growing season differences [ref. 10]. Other Mediterranean ^14^C time series, although shorter, also fail to indicate a consistent offset compatible with the latitude hypothesis (Figs. 4, Supplementary Fig. S9, [ref. 13])—if they indicate offsets either they appear to vary [refs. 5, 8, 10, 13], or be of a larger scale where likely differences in measurement technologies dominate (as we suspect for the MBA Anatolian juniper case in Fig. 1). Therefore, it seems unlikely that latitude explains the average older ^14^C ages in the AA Gordion series. This leaves the difference to be explained by other factors.

Another possible source of an offset that could be applicable to the BCP data, but which over time could change (oscillate), and so explain a variable (lower frequency) offset (see above), is via the effects (through entrained air masses) of changes in upwelling from the Pacific along the west coast of North America, where much higher ΔR ages are observed for surface waters when compared to those around e.g. Ireland ([refs. S17–S19]; see <http://calib.org/marine/>) and as unlikely relevant to inland continental loci (e.g. Germany, central Anatolia). Suggestions that coastal upwelling or other sources of depleted ^14^CO_2_ could contribute to modest depletions in ^14^C recorded in tree rings via entrained air parcels have been proposed previously to explain other apparent local-regional offsets in ^14^C data from dendrochronologically dated tree-rings (e.g. [refs. S20, S21]). As a starting point, changes in storm tracks over the Pacific, and, especially the state of the Pacific Decadal Oscillation (PDO), influence atmospheric circulation and climate over the southwestern parts of the USA [ref. S22]. Investigations of relatively recent periods via foraminifera suggest that there is a correlation between upwelling and changes in ^14^C ages and the state of the North Pacific Gyre Oscillation (NPGO) [refs. S23, S24]—further, the state of the NPGO appears to correlate with, and potentially explain, at least one tree-ring archive of hydroclimate from Southern California [ref. S23]. Over the longer-term, changes in the NPGO range from decadal-scale, explained through connections with wider Pacific decadal-scale dynamics [ref. S25] through to likely associations with low frequency oscillations in the wider North Pacific Gyre system, including a millennial scale oscillation that has been associated with (via assumed common external forcing) the Bond Ice*-*rafted debris (IRD) cycles [ref. S26] in the North Atlantic [ref. S27], and perhaps major centennial-scale episodes like the ‘Dark Ages Cold Period’ or Late Antique Little Ice Age [ref. S28]. One route to track a possible ocean-system association might be via BCP stable isotope records. Changes here might be explained by variations in wider Pacific-region climate regimes and thus changes in typical storm trajectories. Although one study reported an apparent major shift in BCP δ^18^O and suggested such associations [ref. S29], further investigation by another group did not reproduce the marked changes in δ^18^O [ref. S30], and the authors of ref. [S29] subsequently withdrew their dataset [ref. S31]. A stable carbon isotope record from BCP for the last 1000 years indicates associations with El Niño-southern oscillation (ENSO) events, and some possible low frequency patterning might be observed also in the δ^13^C data and precipitation reconstructions presented [ref. S32]. Records of δ^13^C and δ^18^O from BCP for 1710-2010 CE exhibit associations both with ENSO and PDO, but also highlight the complications of differing responses among BCP according to elevation and specific growth context [ref. S13]. The subject merits further investigation.

A final topic of relevance to our discussions around inter-laboratory ^14^C offsets concerns the offset for Anatolian juniper samples versus IntCal20 and how this may affect calibration in the period 1700–1500 BCE. Comparison of the AA GOR time series against IntCal20 shows a weighted average offset [ref. 42] of 11.2±1.9 ^14^C years (AA GOR older). For the period 1600-1540 BCE this offset is slightly larger again at 16.2±3.2 ^14^C years. However, we may also observe that AA data in general appears older than the IntCal20 consensus and, e.g. ETH data. AA IrO data are, weighted average, 5.8±2.7 ^14^C years older than IntCal20 (above). Moreover, since IntCal20 includes the AA IrO and AA BCP datasets, this laboratory offset is in fact being minimized. The comparison of AA IrO versus measurements on the very same wood (87 pairs) at Zurich (ETH), unsurprisingly indicates a slightly larger weighted average difference of

-6.8±2.9 ^14^C years (AA older) [ref. 39], and, combining with an inter-tree comparison yielding

-5.8±2.3 ^14^C years (AA older) (another 127 pairs), this becomes an average difference, ETH to AA, of -6.2±1.8 ^14^C years (AA older) [ref. 39]. Therefore, the direct AA GOR to IntCal20 comparison noted above needs to be modified by this general AA offset to older ^14^C ages in this period. For example, if we remove the overall ETH to AA IrO offset, we might estimate an overall offset of ~5±2.6 ^14^C years. The offset in the period 1600–1540 BCE would become ~10±3.7 ^14^C years. We can in addition quantify the intra-laboratory AA data difference between AA GOR and AA IrO for the periods where both datasets have information. This gives a suitable indication of the difference between a northern European dataset versus GOR.

Comparison of the same laboratory AA measurements on IrO versus the AA GOR dataset is reported as yielding a weighted mean difference of -8.4±3.4 ^14^C years [ref. 39]. This is stated as being from 93 pairs of comparisons. 93 AA GOR data are listed for 3624 to 3532 Cal BP in the supporting online material to ref. [39], but there are only 91 total AA IrO data published over this same period – there are no AA IrO values for 3617 and 3616 Cal BP in either refs. [38, 39]. We determine a similar difference with AA GOR of 8.5±3.5 ^14^C years older across the 91 pairs with AA GOR placed as in refs. [38, 39]. If the AA GOR data set is moved 3 years older with GOR RY 1764 at 748 BCE (see main text, see Supplementary Discussion 2), then this becomes 5.5±3.5 ^14^C years. But there are rather more than 91 pairs of data to compare between AA GOR and AA IrO. In all there are 186 published AA GOR data and AA IrO data have been published for 123 of these years (3624 to 3462 Cal BP) using the refs. [38, 39] placement of AA GOR and for 126 years (3627 to 3462 Cal BP) using the 3 year earlier placement favoured in our discussion below (AA IrO data in refs. [38, 39]). At the refs. [38, 39] AA GOR placement, the overall n=123 difference is 11.3±3.0 ^14^C years; if the 3-year earlier placement as favoured below is used, the overall n=126 difference is 8.3±2.9 ^14^C years. One additional issue is whether to include all the AA GOR data in such comparisons, since there are a few extreme variations within the dataset that appear implausible (and for example ref. [38] itself excluded 5 values when considering their χ^2^ analysis). A plot of the paired differences for both fit positions shows the tendency for AA GOR to be older, as above, but with extreme values in both directions forming a relatively symmetrical distribution (Supplementary Fig. S17). We discuss the best fit of the Gordion tree-ring chronology below (Supplementary Discussion 2) and explain why we favour the placement with GOR RY 1764 at ~748 BCE. This then suggests an average 8.3±2.9 or (date set used in ref. 39) 5.5±3.5 ^14^C years GOR offset versus a northern European (IrO) record. But, within this average there is marked variation (Fig. 3f). In the period between 1600-1540 BCE the difference between the AA GOR data and the available AA IrO (n=32 pairs) is also a little larger at 11.5±6.0 ^14^C years (repeating the indication of a small increase in this period seen also against IntCal20, see above).

There is also a second set of comparative data: Hd GeO versus Hd GOR [ref. 10]. On average across the whole period of comparison, the net difference is small. At the Fig. 3a, c placement the difference between Hd GOR and Hd GeO for 114 Hd GOR data (or weighted averages) versus a 1 year interpolation of Hd GeO is -0.33±2.1 ^14^C years and for the period encompassing mid-points 1705–1486 BCE the difference then for 25 Hd GOR data versus the 1-year Hd GeO interpolation is -3.1±4.3 ^14^C years. However, at the same time, within the comparison period, there is substantial variation as shown in Fig. 3c (and see [ref. 10] which compared two-sided 1-year or 0.5 year interpolations). It is notable that the Hd GOR data are older than Hd GeO especially ~1600–1550 BCE (data with mid-points ~1595–1555 BCE) [ref. 10]. Across the 8 pairs of comparisons available between the Hd GOR data and a 1-year interpolation of the Hd GeO data the difference here is Hd GOR older by 16.4±7.6 ^14^C years. But this is the very period when the Hd GeO are conspicuously, and for some unknown reason, too recent [refs. 1, 43, 49]. As noted in the main text, the Hd GeO data 3629–3449 Cal BP (1680–1500 BCE) are -15.6±2.4 ^14^C years versus IntCal20, n=57. Or, when compared versus ETH IrO [ref. 39] (weighted averages) for this period (common data available 3625–3431 Cal BP/1676–1482 BCE), the Hd GeO are -11.8±2.8 ^14^C years, n=49. And, in particular, it has been shown that the Hd data on the Knetzgau 40 tree [ref. 10] are -12.9±3.1 ^14^C years more recent than measurements by three other laboratories on this same tree [ref. 49]. The Hd GeO data in this interval are clearly too recent. However, at the same time, as we argue in the main text, it seems likely that the Hd GOR data in this interval are very likely correctly reflecting ^14^C levels and hence are very similar on average with IntCal20 (with IntCal20 in this period likely already including any Mediterranean offset, see above). The Hd GOR dataset overall is +2.3/2.6±2.1 ^14^C years versus IntCal20 (n=117)—note the variation depends on whether the Hd GOR data with 0.5 calendar year mid-points when wiggle-matched are rounded up or down versus IntCal whole year data (in the main text and Supplementary Fig. S8 we employ the smaller rounded up version). In the period between 1600–1550 BCE, the difference Hd GOR v. IntCal20 is -1.1±6.6 ^14^C years (n=8), so again very similar. The larger difference at this period of Hd GOR versus the Hd GeO thus likely reflects the problem that these Hd GeO data are substantially too recent for an unknown reason. The availability of all the new IntCal20 comparative data and the reassessment of the Hd GeO data in the ~1660–1540 BCE period thus leads to a re-assessment revising previous observations made concerning the Hd GOR v. Hd GeO offset in ref [10]. The apparent Hd GeO to Hd GOR offsets noted there in the earlier 16^th^ century BCE, or above based on 8 data pairs for sample mid-points between ~1595 to 1555 BCE, should therefore likely be greatly reduced based on the re-assessment of these Hd GeO values. The Hd GOR data, meanwhile, suggest an overall close average correspondence with IntCal20, but with some periods of variation in both directions (Fig. 3c). For example, ~1377–1317 BCE (3326–3266 Cal BP) there is an average offset of Hd GOR to IntCal20 of +15.7±6.7 ^14^C years (n=10), and again ~1127–997 BCE (3076–2946 Cal BP) there is an average off of Hd GOR to IntCal20 of +20.7±5.5 ^14^C years (n=19). Such positive offsets seem associated with major reversals and plateaus in the calibration curve as noted before [refs. 8, 10]. At these times there may be a relevant calibration offset that affects the correct estimation of calendar ages in the eastern Mediterranean region.

A key question for chronology in the 1700–1500 BCE interval is therefore whether the reversal and plateau ~1600–1540 BCE is a period when such a Mediterranean offset seems to apply? As discussed in the main text, and above in this section, there is the additional factor that across this interval the inclusion of hundreds of AA data on IrO and especially BCP have already overly raised the IntCal20 curve, since the AA data generally are a little too old (e.g. AA IrO versus IntCal20 or especially parallel ETH IrO) and the BCP data are themselves a little too old and then as measured by AA they are substantially older again. This has thus created a likely maximum (versus real central-northern European) height wiggle (MHW) in the earlier to mid-16^th^ century BCE. Therefore, even if there was likely a small Mediterranean offset at this time, it is perhaps already incorporated in (or hidden by) the slightly inflated IntCal20 values across this period. The Hd GOR data which more or less correspond with IntCal20 over this period suggest this. Further, with the re-assessment of the Hd GeO data in this period (above), there is now likely no real evidence for any substantial difference between the Hd GeO and Hd GOR data in the earlier 16^th^ century BCE, contrary previous assessments [ref. 10] using what are now appreciated to be too recent Hd GeO data in this interval. The Groningen (GrM) data series from Noceto (NOC), Italy, further support the assessment that Mediterranean trees have ^14^C levels at this time similar to the values in IntCal20 (which, as noted above, may be slightly inflated). As wiggle-matched against IntCal20 (Fig. 4a), the NOC data are on average 1.7±6.1 ^14^C years older than IntCal20 (or, if the largest difference is excluded, -1.1±6.2 ^14^C years)—i.e., they are very close. This situation with IntCal20 is very different than with the previous IntCal13 [ref. 27]. Whereas formerly the GrM NOC data were offset (older) versus IntCal13 in the earlier 16^th^ century BCE [ref. 10], they are now very close to IntCal20. This suggests that IntCal20—with the effect of the hundreds of older AA IrO and especially BCP data—is approximately accounting for any Mediterranean offset in the 16^th^ century BCE and is thus suitably representing the ^14^C record for the Mediterranean over this period.

The potential complication comprises the AA GOR dataset. The intra-laboratory comparison (with AA GOR placed with GOR RY1764 at 748 BCE) indicates an average difference between AA GOR versus AA IrO of 8.3±2.9 ^14^C years. The adjusted comparison versus IntCal discussed above suggested an offset of ~5±2.6 ^14^C years. These values, and especially the latter, are not large, but they nonetheless suggest some positive offset. In addition, it is observed that the AA GOR values for the period 1600–1540 BCE are a little more positively offset, and, even adjusted by the ETH–AA factor, are offset from IntCal20 by around 10.0±3.7 ^14^C years (n=61). This increased offset corresponds to the reversal and plateau in the calibration curve, consistent with other observations (above, see refs. [8, 10]), and the various smoothed versions of the AA GOR data shown in Fig. 3e, Supplementary Figs. 6, 8, illustrate this pattern. The major concern, however, is that the AA GOR dataset includes several major outliers and considerable general noise. As discussed in Supplementary Discussion 2 (below), a χ^2^ reduced comparison of the AA GOR series with either IntCal20, or the weighted average of the ETH + AA IrO and AA BCP, indicates a relatively poor fit (with χ^2^ reduced values of ~1.53 or ~1.59 respectively), suggesting the presence of one or more of outliers or insufficient measurement errors or a laboratory offset. The unusual and dramatic spike AA GOR RY959–961, ~1553–1551 BCE or 3502–3500 Cal BP, if real, and not a case similar to the several other major outliers (older and more recent) in the dataset, requires an explanation, for example. If only data for differences between AA GOR and IntCal20 within 1σ of the set mean (n=186) were considered over the period 1600–1540 BCE (n=44), and the same AA–ETH adjustment then applied, the apparent offset is much smaller at 2.8±3.7 ^14^C years and would be regarded as negligible. As another example of the effect of the noise/outliers, if we consider the χ^2^ fit of the AA GOR data series versus IntCal20 and exclude those AA GOR data (n=16) where at the minimum (all data) best fit the difference of the AA GOR date versus IntCal20 is >2 times the measurement error (>2SD) of the AA GOR date, then the revised best fit has a minimum value at 749 BCE (the χ^2^ reduced value is ~0.76 – contrast the all data value versus IntCal20 of ~1.53) and the weighted average difference of the (remaining n=170) AA GOR data v. IntCal20 is 9.9±2.0 ^14^C years (the AA GOR data excluded are RY851, 884, 885, 905, 909, 911, 918, 921, 924, 939, 951, 959, 960, 961, 1000, 1001). This placement of the AA GOR dates against IntCal20 and with the 16 excluded dates (>2SD difference to IntCal20) indicated is shown in Supplementary Fig. S14. The excessive ‘noise’ removed is apparent. If the ETH IrO to AA IrO difference (-6.2±1.8 ^14^C years) or the AA IrO to IntCal20 difference (-5.8±2.7 ^14^C years) (see above) were subtracted, then this would indicate a more likely real offset between AA GOR and IntCal20 of just 3.7±2.7 ^14^C years or 4.1±3.4 ^14^C years respectively. This is small to negligible. The apparent increased offset 1600-1540 BCE is also much reduced to an additional and small to negligible 1.5±4.0 ^14^C years. Such a ‘cleaned’ AA GOR dataset might, after allowing for the apparent AA old-age offset versus either IntCal20 (the combination of all calibration laboratory data for this interval) or ETH, in fact indicate that the GOR ^14^C data are close to IntCal20 and with any typical Mediterranean growing season offset already included in this period in IntCal20 because of the AA inflation factor included in IntCal20 for the interval 1700–1500 BCE (see above). Thus there would be no reason to assume any substantive additional ^14^C offset beyond IntCal20 in this specific interval. (Note: this point is specific to the 1700-1500 BCE interval with the AA IrO and AA BCP datasets under discussion inflating the age range of IntCal20—elsewhere we may expect some potential, variable, small Mediterranean growing-season-related ^14^C offsets at some periods [refs. 8, 10].) Hence we suggest in the main text that with the evident inter-laboratory offset removed (whether based on AA IrO to IntCal20 or AA IrO to ETH IrO), and with excessive noise removed from the AA GOR data series, there is little evidence to indicate that east Mediterranean ^14^C ages in the 1700–1500 BCE interval are substantively different from IntCal20 (which, as noted above, are already inflated through the inclusion of hundreds of AA ^14^C data on IrO and especially BCP). This is an important and key issue for the resolution of chronology across the period from the late 17^th^ to mid-16^th^ centuries BCE. As we illustrate in a consideration of the dating of the Thera eruption, the shape of the calibration curve means that an offset at this time of even about 8 ^14^C years can be critical to resolution between a likely late 17^th^ century BCE date versus an earlier-mid 16^th^ century BCE date (Fig. 6, Supplementary Figs. S11, S15). Hence, the fact we can likely rule this out, favours the result shown in Fig. 6a. Nonetheless, looking forward, it is clear that robust resolution of whether or not there is a Mediterranean offset in the earlier-mid 16^th^ century BCE, and, if so, its quantification, is essential to resolve high-resolution chronology around this period.

One interesting common feature of both the Hd GOR and AA Gor datasets may be noted. Both datasets indicate a sharp dip in regional ^14^C shortly before 1600 BCE (Fig. 3a, e, Supplementary Fig. S9), beyond the level represented in IntCal20. This could be an interesting regional ‘marker’.

***2. Wiggle-match placement of the Gordion tree-ring series***

There are two ^14^C time series based on Gordion (GOR) tree rings: (i) the Hd GOR series [refs. 5, 10, 37], and the AA GOR series [refs. 38, 39]. The overall Gordion tree-ring chronology runs from Relative Years (RY) 737 to 1764. To best enable comparison with the discussion in ref. [38], we refer in the following discussion to the placement of the GOR chronology in terms of the felling date, represented by RY1764 which preserves bark. For reference, the analysis in ref. [10] against IntCal04 [ref. 32] placed GOR RY776.5 at 3686±1 Cal BP which in turn places GOR RY1764 at 750±1 BCE. We first consider wiggle-match placements of these two series against IntCal20 [ref. 1].

*Hd GOR*. An OxCal wiggle-match [ref. 22] with all data (data for the same tree-ring mid-points combined as weighted averages [ref. 42]) against IntCal20 using the OxCal D_Sequence function and the SSimple Outlier model [ref. 24] places the chronology, μ±σ, ~1777–750 BCE (±1). The 95.4% range for GOR RY1764 is 752–747 BCE. The OxCal A_model_ and A_overall_ values are predictably poor given noise within such a large dataset with no cleaning of outliers. To achieve almost, or greater than acceptable, OxCal A_model_ and A_overall_ values (>60), it is necessary to remove the 13 or 14 largest outliers within the dataset (outliers indicated in Supplementary Table S3). The fit placement moves 2 years more recent with RY1764 placed, μ±σ, ~748 BCE/2697 Cal BP (±1) (Fig. 3d). The 95.4% range for GOR RY1764 is now 751–745 BCE. We regard this as the best placement versus IntCal20.

*AA GOR*. Another series of 186 annual (single tree-ring) ^14^C measurements has been published on samples from the same Gordion chronology, with the ^14^C ages measured at Arizona, AA [refs. 38, 39]. Note: we use the AA GOR data listed in ref. 38—the data listed in ref. 39 are missing AA110981 and AA110980. If this time series is wiggle-matched against IntCal20 with OxCal it is placed (μ±σ) 1678±1 BCE (GOR RY 834) to 1493±1 BCE (GOR RY 1019) (Supplementary Fig. S6). The extrapolated 95.4% range for GOR RY1764 is 751-746 BCE, the μ±σ = 748±1 BCE. Thus this AA GOR fit with OxCal and IntCal20 is identical to the Hd GOR fit (with 13 or 14 major outliers removed) as shown and analyzed in Fig. 3a, c (and this correspondence of date placements acts as additional reason to use the Hd GOR fit minus main outliers, as above). It is striking that these wiggle-match results are effectively identical (despite the AA to Hd offset, see Supplementary Discussion 1, and the noise evident in the AA GOR dataset, Fig. 3e, f, Supplementary Figs. S6, S9, S14)—illustrating that ^14^C wiggle-matches of long time series should yield accurate results and should be robust and stable within narrow margins. We note that this OxCal wiggle-match does not use the SSimple Outlier model, and instead replicates the analysis in ref. [38] (but with the correct RY intervals – see below). If the SSimple Outlier model is used, OxCal struggles with the very noisy AA GOR dataset. It finds a much wider range of possible fits (which vary by a few years over repeated runs) although the mean values from the various runs are similar within a few years of variation and are around the same region of ~750 BCE.

It must be noted that the gaps at the start and end of the GOR chronology used in ref. [38] are not correct. The ^14^C data are from GOR RY834–1019 of the 1028-year Gordion tree-ring chronology. Ref. [38] (caption to Fig.1) stated that “A gap of 103 years was added to the start of the single year sequence and 744 years were added at the end to represent the entire chronology”. These numbers are incorrect. There are inclusively 97 years from RY834 to RY737, and 745 years from RY1019 to RY1764.

The publication of the AA GOR data preferred a best fit from a χ^2^ least squares approach [ref. 38]. We thus consider a similar approach to check the fit determined in this case from OxCal.

We use two methods: (i) a well-known published least squares ^14^C wiggle-match method [ref. 40] without allowing for the measurement errors on either the observed or expected data, and (ii) a χ^2^ approach weighting the differences according to the respective measurement errors [ref. 41] – note the sign is incorrect (it should be minus, not plus) in the top line in the formula given in ref. [41] p.577. Thus we employ:

$$\chi{(x)}^{2}=\sum_{i=1}^{n} \left( R_{obs}-R_{exp} \right)^{2}/({{\sigma R}_{obs}}^{2}\text{ + }{{\sigma R}_{exp}}^{2}\text{)}$$

Where R_obs_ is the AA GOR ^14^C value (or observed data) and R_exp_ is the expected ^14^C value (either IntCal20 or the weighted average of the ETH IrO + AA IrO + AA BCP), and σR_obs_ and σR_exp_ are the respective measurement errors on each of R_obs_ and R_exp_. The χ^2^ value should become minimal for the correct fit year: *x*. The latter approach should yield smaller fit ranges.

First let us consider the least squares (no errors) approach. Ref. [38] noted that 5 of the AA GOR data were excluded, writing: “Data points outside 3σ were removed from the Gordion juniper sequence (*n* = 5).” However, which 5 data points were removed is not specified, nor what the 3σ refers to. If we consider the individual AA GOR data against a weighted adjacent average curve through the dataset, then with a 5-point (sample/date) window there are just 4 dates >3σ from the mean (in order of scale of outlier: RY905, 885, 939, 884), with a 10-point window then there are 5 dates >3σ from the mean (in order of scale of outlier: RY905, 939, 885, 884, 1000), and with a 20-point window it is 7 dates >3σ from the mean (in order of scale of outlier: RY905, 939, 884, 1000, 885, 961, 1001). We thus consider an all-data (ALLD) fit and a fit with 181 dates, excluding the 5 dates RY905, 939, 885, 884 and 1000 (EXCL5). We consider a fit against (i) IntCal20, and (ii) a weighted average of the IrO and BCP data sets in refs. [38, 39] (as used in [ref. 38]). We calculate the best fit (minimum point), the 95.4% fit region, and the 68.3% fit region in each case following the ref. [40] method: see Supplementary Fig. S7A.

Against IntCal20 we find the best (minimum) fit expressed (as in [ref. 38]) in terms of the last ring of the Gordion chronology, GOR RY1764, using either the whole 186 date set (ALLD), or, as in the original publication, 181 dates minus the 5 largest outliers (EXCL5), at 749 BCE. The 95.4% fit region is 760–743 BCE (ALLD) and 760–743 (EXCL5); the 68.3% regions are 754–746 BCE (ALLD) and 753–746 BCE (EXCL5). If the weighted average of the IrO and BCP dataset is used, as in the original publication [ref. 38], then the best fit is 747 BCE. The 95.4% ranges are 759–741 BCE (ALLD) and 759–744 BCE (EXCL5) (note: of course small variations can occur depending on exactly which 5 data are identified, by what method, as the largest outliers—this point was not clarified in ref. [38]). These results are, of course, very similar to those from OxCal (above).

Second, let us consider the weighted χ^2^ approach, see Supplementary Fig S7B. Against IntCal20 there is no satisfactory fit at the 95% level but the minimum is placed 748 BCE. Against the ETH IrO + AA IrO + AA BCP weighted average (hereafter ETH+AA wAV) the minimum is placed at 747 BCE. Using this approach we can also considering the reduced χ^2^, thus χ^2^/df (where df = degrees of freedom), to assess goodness of fit. A fit with a χ^2^ reduced value of ~1 indicates a good fit of data, whereas values (progressively) >1 indicate a poor fit, and suggest either issues in the data and/or that the error variance has been underestimated. In our current cases we can immediately observe that in fact the fit of the AA GOR data against either comparison is relatively poor. The χ^2^ reduced value for the fit against IntCal20 is ~1.53 and against the ETH+AA wAV it is ~1.59. Looking at the AA GOR data we may observe some substantial apparent noise versus IntCal20 (Fig. 3e, f, Supplementary Fig. S6). We might therefore consider excluding some of the largest outliers. Starting with IntCal20 as the comparison, if we use the EXCL5 set above, then the minimum remains 748 BCE, there is a 95% fit range of 749–747 BCE and the χ^2^ reduced value is ~1.17. Alternatively, since the largest differences in the χ^2^ analysis are not always the same as those identified above, if we remove the 5 largest differences in the all-data χ^2^ analysis (RY905, 939, 961, 1000, 1001) the minimum again remains at 748 BCE, the 95% range becomes 752–746 BCE and the χ^2^ reduced value is ~1.14. If we remove all 9 cases where the difference of AA GOR minus IntCal20 is > 3 times the AA GOR measurement’s σ (RY884, 885, 905, 921, 939, 960, 961, 1000, 1001), then the minimum shifts to 749 BCE, the 95% fit range is 759–742 BCE and the χ^2^ reduced now dips under 1 at ~0.92. As well as removing some outliers, we might also wonder whether the typical quoted AA error is a little too small, or if there is an inter-lab offset, when nearly 5% of data (versus an expected 0.3% of data) are greater than 3SD away from expected value. We will return to this issue, but let us look first at the comparison versus the ETH+AA wAV dataset. The all-data comparison places the minimum at 747 BCE but offers no 95% level fit, the EXCL5 selection does the same. If the 7 instances where the difference of AA GOR minus ETH+AA wAV is > 3 times the AA GOR measurement’s σ are excluded (RY884, 885, 905, 939, 960, 961, 1000), then the minimum shifts to 748 BCE, the 95% fit range is 749 or 747 BCE, and the χ^2^ reduced value is ~1.16. The minimum fit points and ranges are all very consistent, as expected, with the OxCal analysis. However, again, the χ^2^ values would suggest either (or both) the AA errors are a little too small or there is some offset. If we were, arbitrarily, to add an additional 1‰ (±8 ^14^C years) error to all the AA GOR data (thus revised errors = √(AAσ^2^ + 8^2^)), then with IntCal20 the best fit minimum with all data is 749 BCE but still with no 95% level fit range. The 3 largest differences need to be removed (RY905, 939, 1000) to get a 95% fit range (minimum 748 BCE, range 750–747 BCE). The χ^2^ reduced value is ~1.16. By the time the 5 largest differences are removed (adding RY961, 1001) then the fit is at 748 BCE, the 95% range is 758–744 BCE, and the χ^2^ reduced value is ~1.04. In all there are 8 data where the difference is greater than 3 times the (enlarged) σ (RY884, 885, 905, 921, 939, 961, 1000, 1001). Excluding these data the best fit minimum is 749 BCE, the 95% range is 761–741 BCE and the χ^2^ reduced value is 0.88. Against the ETH+AA wAV the minimum best fit with all data is 747 BCE but the 4 largest differences need to be removed (RY885, 905, 939, 1001) before there is a 95% level fit range, of just 747 BCE. The χ^2^ reduced value is ~1.15. In all there are 6 data where the difference of AA GOR minus ETH+AA wAV is > 3 times the AA GOR measurement’s σ (RY885, 905, 921, 939, 1000, 1001). If these data are removed then the best fit minimum is 746 BCE and the 95% range is 749–745 BCE and the χ^2^ reduced value is ~1.05.

The conclusion is that the range of analyses (OxCal and χ^2^) suggest best fit points between 749 BCE and (in one case) 746 BCE with most 749 BCE to 747 BCE. The frequency of mentions as the best fit in the discussion in this section is 748 BCE (6), 747 BCE (4), 749 BCE (3), 746 BCE (1), and 750 BCE (1). On this basis ~748±1 BCE appears a good best consensus estimate for the placing of GOR RY1764, and we employ ~748 BCE for the analyses in this paper.

It is also evident from this analysis that some of the AA GOR data are likely major outliers. We therefore include consideration of a ‘cleaned’ AA dataset with the data with differences versus IntCal20 greater than 2 times the AA GOR measurement error (>2SD) excluded (n=16), when discussing and assessing the apparent inter-laboratory differences present and the likely real relationship of the GOR ^14^C values versus IntCal20 (see Supplementary Discussion 1 above). See also Supplementary Fig. S14.

*Overall GOR placement*. Expressed in terms of GOR RY1764, the set of these ‘best’ fits for the two ^14^C time series, both Hd GOR and AA GOR, with or without outliers and against different referents are all very similar. They are however, ~1–5 years older than the preferred, and supposedly absolute, best fit recently published [ref. 38]. As noted above, we use ~748 BCE in this paper (+3 years versus ref. [38]). It was argued that this claimed 745 BCE fit was supported by a superposed epoch analysis (SEA) [ref. 38]. This SEA analysis was stated to come from analysis of a “1,979-year Bronze–Iron Age juniper chronology” of which the GOR chronology is a sub-set. However, what exactly this chronology comprises is unclear, since the reference cited “(17)” in ref. [38] is to a paper published in 1996 that discusses a then-supposed 1503-year chronology (this is our [ref. S33])—no tree-ring data were provided there. Regardless, the key problem is that the pre-Gordion portions of this supposed chronology, or other proposed chronologies (but with data also not provided) (e.g. [ref. S34]) are not securely crossdated (in dendrochronological terms) with the Gordion chronology (see discussion in [ref. 18]). Thus there is no robust “1,979-year Bronze-Iron Age juniper chronology” currently published, and the SEA analysis is therefore not valid and provides no support for the claimed 745 BCE date. We instead prefer the fits indicated by the analyses of both the Hd GOR and AA GOR time series (excluding outliers) 2–4 years earlier, and we use a date of 748±1 BCE for RY 1764 as our current best estimate. Assuming the wood was used within 0–1 year of felling, GOR RY1764, with bark, likely dates construction of the Midas Mound Tumulus at Gordion within 1 year, revising previous assessments [refs. 5, 10, 18, 37, 38].

While only ~3 years older than the supposed absolute date published [ref. 38], the proposed re-dating above nonetheless suggests a need to revise the specific absolute annual dates and thence external associations proposed in ref. [38]. This may well invalidate the specific-year associations suggested in ref. [38] with regards to major environmental events, such as volcanic eruptions. This highlights the need to focus very closely on details and on accurate and precise dates and associations once timescales approach annual resolution in order to avoid a 21^st^-century high-resolution version of the ‘suck-in and smear’ problem [ref. S35].

***3. History and context of the Thera/Santorini dating debate and revised ^14^C dating models***

*Introduction*

One of the five largest volcanic eruptions of the past 10,000 years by volume [ref. S36], the enormous eruption of the Thera (or Santorini) volcano in the second millennium BCE has a rich modern scholarly profile since its 19^th^ century recognition [refs. S37–S45]. The striking volcanic remains, and extensive prehistoric settlement found buried under the pumice at Akrotiri on Thera with its sophisticated multi-storied buildings and the many now famous wall-paintings, have all combined to capture widespread attention [refs. S41, S44–S49]. At various times over the years the eruption has been claimed to be the source of the Atlantis story [refs. S41, S50], or events in Exodus [refs. S51, S52]. More specific has been the question of its relationship with another big event: the horizon of Late Minoan (LM) IB destructions on Crete that mark the end of the New Palace period on the island [refs. S38, S53]. Was the Thera eruption to blame for the desolation of Crete, whether directly [refs. S54, S55] or indirectly [refs. S38, S56–S58]? It thus seems obvious that dating this event is inherently of significance.

The Thera eruption used to have a settled approximate date: about 1500 BCE [refs. S54, S59, S60]. Science-based dating methods then intervened. A date in the 17^th^ century BCE was suggested from possible proxy representations in tree-rings and ice-cores [refs. S61–S64]. ^14^C either seemed to support such radical revision, or, indicated this as a likely date range, but simultaneously offered a less likely alternative in the mid-16^th^ century BCE, while pretty definitively ruling out the conventional date range of ~1500 BCE [refs. 2, 52, 53, 54, 56, S40–S42, S62, S65–S68]. This was unless one chose to dismiss the ^14^C evidence and maintain the conventional assessment, regardless [ref. S69]. A ‘wiggle’ in the ^14^C calibration curve created a sort of quantum uncertainty. The result: an unsteady date for a big bang [ref. S70] prompting great scholarly activity, clamor and incompatible positions for more than 40 years [refs. 33, S40, S42, S71, S72]. Would-be solutions [refs. 56, S73] invariably hit sharp criticism [refs. 55, 68, S74], and new datasets critically revised past assessments [ref. 43]. Now, in 2020, a new international ^14^C calibration dataset, IntCal20 [ref. 1] is available, notably incorporating a staggeringly massive investment of effort and funding to better define the era of the Minoan eruption of Thera [refs. 1, 2]. It is therefore appropriate to take stock. We review the context and history, and then move to the date.

*The eruption*

Predictably, a beautiful shattered volcanic island set in the south Aegean offering sea, sunshine, stunning views, noted sunsets, and a buried unique archaeological site [refs. S41, S44, S47–S49] has been a focus of much attention, from scholars, as well as many (indeed too many) tourists [ref. S75]. Thera is one of the better-studied major volcanoes [refs. S36, S37, S39, S41, S48, S76–S81]. The Minoan eruption blanketed a large area of the southern Aegean, parts of Anatolia, and some of the east Mediterranean in tephra (volcanic ash) [refs. S82–S88], forming one of the numerous marker tephra layers that delineate the tephra-stratigraphy of the region [refs. S85, S88]. Minoan eruption Thera tephra fall covered archaeological sites, perhaps at a depth to precipitate structural damage [ref. S89] in some areas to the east especially (e.g. Kos, Rhodes [refs. S90–S93]), and appears to have had substantive effects associated with abandonment of some areas and town re-planning at Trianda on Rhodes [ref. S93]. Recognizable layers, to traces, are also found to the south on Crete in the landscape [refs. S94, S95] and at archaeological sites [refs. S96–S98]. Even modest (>1cm) tephra falls and especially associated chemicals can cause immediate to longer-term devastation to agriculture and livestock [refs. S89, S99–S102]. Tsunami caused by the eruption may have affected coastal regions in their path [refs. S103–S111]—the northern coasts of Crete being likely candidates, although recognition can be challenging [ref. S112]—with just a few plausible cases recognized in recent years [refs. S109–S111]. However, the environmental records from both northwest Crete and western Anatolia provide only modest indications of the eruption [refs. S113, S114], although some longer-term abandonment of cultivated areas producing cereals and olives is evident in the Cretan case [ref. S113]. Overall, the general assessment has been that direct damage on Crete and in the wider Aegean was in fact less than overwhelming, or the population very resilient, or both [refs. S38, S56, S58], and the eruption did not seem to correlate with any major horizon of cultural, economic or political change [refs. S38, S53, S56–S58, S115]—with parts of the east Aegean the possible exceptions [refs. S90–S93]. Indeed, while always recognized as a large-magnitude event, Thera, until recently, seemed to punch under its apparent weight. It was held self-evidently to be a large volcanic event, but not enormous [ref. S116, S117], and, critically, its total sulfur release—a major mechanism and measure for climate impact [ref. S118]—was thought modest for such a big event [refs. S116, S119, S120]. As a result, and in the absence of clear evidence to the contrary, its global or long-term impact was also considered to be limited [refs. S56, S58, S115, S116, S119, S121].

This former assessment has been substantially revised in recent years. The eruption’s true scale is now estimated as the largest volcanic eruption of the Holocene in terms of dense rock equivalent emitted [ref. S36], and it is also recognized (after studies of the 1991 Pinatubo eruption in particular) that the key volatile releases that determine climate impact were under-estimated or over-looked in past work [refs. S122, S123]. A likely pre-eruption fluid phase will have contained and released much greater quantities of sulfur and other volatiles. Depending on variables, including especially the amount injected into the stratosphere, climate impacts in the northern hemisphere (surface cooling) of one to a few years may be anticipated, with substantial depletion of (pre-industrial) ozone in the stratosphere, taking up to a decade to recover [ref. S123]. It was argued in the past that the previous (much smaller) assessments of the eruption’s emissions meant that it was likely too small to register clearly in Greenland ice records (refs. S116, S119]. This viewpoint now needs to be reassessed—a signal is in fact likely based on the work of ref. [S123].

Overall, the Thera eruption, likely in the late spring or summer months [refs. S87, S115, S124], probably should have had a major shorter-term environmental impact regionally where tephra falls were >1cm (potentially devastating crops ready for, or just harvested, maximizing the human challenge in its aftermath). There should also be a recognizable northern hemisphere climate signal over the following one or more years. Recent work suggests a potentially coincident change in climate regime in the Nile Delta [ref. S125], perhaps similar, via analogy, to the suppression of the Nile floods observed in historic periods after volcanic eruptions linked in turn to social-political impacts in Egypt [ref. S126]. While speculative, such changes might potentially offer a partial cause for a weakening/decline of the (until then regionally dominant) Hyksos world-system that was based in the Nile Delta and (whether after a few years to a few decades) part of the explanation for why the Theban dynasty was able eventually then to defeat them, and push them out of Egypt under Ahmose, the first ruler of the new 18^th^ Dynasty. Alternatively, the dramatic account of a great storm and flooding associated with the accession of the Egyptian king Ahmose has been proposed as an immediate reflection of the Thera eruption by some [ref. 57], but is questioned by others [ref. S127]. What is without any doubt is that the eruption obliterated what had been, until then, in the form of Thera and its harbours (for discussion of the reconstruction of the pre-eruption island and its central caldera and island, see ref. [S128]), a major international port area [ref. S49] and network node for the wider Aegean [ref. S129]. Akrotiri on Thera was likely one of the larger settlements in the Aegean at the start of the Late Bronze Age [refs. S49, S129, S130], and a number of other sites are also known from the island group [ref. S131]. The population clearly had warnings, via earthquakes and then initial precursory tephra falls, before returning to start repairs and recovery [refs. S132, S133]. The evidence suggests that this (large) human population perished in addition [ref. S132]. These twin losses must have severely impacted Aegean economic and social networks, leading to major post-eruption changes [refs. S38, S56–S58, S129].

In all cases that we might consider around the impact of the Thera eruption, moving from possible effect to causal link involves a temporal component. Which archaeological contexts were contemporary and affected? If the eruption is to be associated with the LM IB destructions, then what is the period of time in-between? Brief enough to suggest a plausible process; too long to be cogent? Identification of potential associations for the eruption among various possible proxy indications of volcanic activity or climate effects likewise requires a date.

*Relative dating*

The question of the date involves two parts: relative and absolute. The relative date is when did the eruption occur in terms of the archaeological sequences known from sites in the Aegean and east Mediterranean. The absolute date is when in calendar time. The relative date is more or less agreed [refs. 52–54, S38, S40–S47, S53, S56–S60, S66, S70–S72, S90–93, S96–98, S109, S110, S115, S129, S130, S133, S134, S135]. The last phase of settlement preserved at Akrotiri on Thera before the town was buried by the eruption is called Late Cycladic (LC) I. This is defined by styles of material culture (especially ceramics). Imported and imitated material place LC I as contemporary with phases known as LM IA on Crete and Late Helladic (LH) I in southern Greece. The subsequent cultural period in Crete (stratified over LM IA) is LM IB and LH IIA on the mainland. There is nothing definitely post-LM IA on Thera (among many, many finds under the pumice). Thus the eruption is confidently placed as late in the LM IA period, and perhaps even at its end (and is then perhaps responsible for some dislocations noted at the end of LM IA [refs. S38, S56, S58]). At sites blanketed by tephra from the eruption, its occurrence is at the close of LM IA, and before LM IB [refs. S90–S93, S96–S98].

The slightly awkward point, however, is that there is a lack of a recognized clear cataclysm in the Aegean at the close of LM IA to match the enormous volcanic eruption. Indeed, it is not until the close of the subsequent LM IB period that there is a major long-recognized horizon of destruction and change [refs. S38, S53, S56–S58]. For a while scholarship wondered if, somehow, the eruption and the end of LM IB could be brought together [refs. S54, S55, S136]. But with further archaeological investigation it became very clear that there is clear separation. Many years of investigations of the LM IB period in fact show that it was a relatively long period [ref. S137], and hence there is a fairly long interval of time between the eruption and the LM IB destructions. Thus a problem: what effects did a very big bang around the close of the LM IA period have? Does one try to find more evidence of previously over-looked destructions and change at the end of LM IA? Or, instead, argue for slow-burning effects whereby the eruption’s substantial—just hard to identify—impacts caused problems that, seemingly many decades to about a century later (but exactly how long?) led to the LM IB destructions and a clear break in the archaeological history of Crete? The absolute timescale and quantifying the intervening period of time is crucial. The shorter the time interval from the Thera eruption to the close of LM IB the more plausible some model where it is the indirect trigger of accelerated decline, breakdown, and transformation [refs. S38, S56–S58, S129]. The longer the gap this logic loses explanatory potency. Further, examination of comparative cases of volcanic disaster suggests that not all changes and adaptations will necessarily be negative, especially over the longer term [ref. S138].

The Aegean’s cultural phases were approximately placed, in relative terms, against the historical chronology of Egypt [refs. S40, S42, S53, S54, S59, S60, S66, S69, S70, S134, S135, S139, S140]. Some mature style LM IB and LH IIA ceramics were found in Egypt in contexts associated with the reign of Tuthmosis III, or in some cases maybe the earlier 18^th^ Dynasty. The 18^th^ Dynasty began in the mid-16^th^ century BCE (e.g. ‘high’ date ~1565 BCE, ‘low’ date ~1540 BCE, from [ref. S141]) and the almost 54-year reign of Tuthmosis III, dated as starting ~1504 BCE, ~1490 BCE or ~1479 BCE according to various Egyptian chronological assessments [refs. 7, 34–36, S141–S143], occupies the first half or so of the 15^th^ century BCE. Thus, give or take the time from manufacture through trade to deposition, at a minimum some part of LM IB and LH IIA appeared approximately coeval with somewhere in the earlier 15^th^ century BCE. But when did the overall LM IB period begin? No secure LM IA object or its Aegean contemporary has yet been found in Egypt. We have to guess. A few much earlier Middle Minoan objects, found in 19^th^-18^th^ century BCE contexts in Egypt, are the next known point heading backwards [refs. S60, S134, S135, S140]. This all leaves a rather loose chronology where prior belief and interpretation weigh more heavily than actual evidence. The conventional date for the Thera eruption, about 1500 BCE, came from associating the end of LM IA and the start of LM IB with the accession of Tuthmosis III (e.g. [refs. S40, S54, S59, S60, S139, S144–S146]). Why? Originally it was about the only fact, of any sort, in play. This worked, sort of, and notwithstanding problems [ref. S140], until scientific dating methods arrived, and, perhaps inevitably, they indicated alternative dates.

*Absolute Dating*

The big debate in East Mediterranean archaeology since the mid-1970s (when ^14^C first started to point to an earlier date [ref. S146]): when, exactly, did the Thera eruption occur? Rival experts supported positions a century apart, a gap sufficiently large as to render positions mutually exclusive. There was also a humanities versus science culture clash [refs. S147, S148], adding frisson. This was despite discussions highlighting that accommodations were potentially possible, and that the burden of tradition was less secure than often appreciated [refs. 52, 53, S40, S66, S140, S148–S150]. Clash or major compromise: such a nexus makes it self-evident that scholars will want to pile in to the debate. Almost by self-definition, the controversy meant that its resolution must be a major aim. Resolving the conundrum would create certainty, and (assumed positivist logic) certainty would lead to an entirely new prehistory of the region. Over the ensuing five decades the relevance of various data and interpretations have come and gone. This winnowing is how academic research moves ahead. A number of tantalizing hints have ended up remaining, so far, just that. For example, frost damage or low growth events in tree rings may link with major volcanic eruptions where trees are susceptible to post-eruption cooling [refs. 66–68, S61, S63], but so far we struggle to know which volcano, and definitely. Similarly, changes in ice-core [refs. S62, S151] or speleothem (stalagmite) [ref. S152] or tree-ring chemistry [ref. S153] may indicate a major volcanic eruption, but, again, even if a volcanic cause is clear or likely, which specific volcano is often unknown unless secure, non-ambiguous volcanic ash/glass particles are recovered—in the case of an ice-core—and this is often a challenge [ref. S154]). As the debate began, so it remains today: ^14^C dating of plant material alive until about the time of the eruption is the key directly relevant scientific evidence on which the debate hinges.

From the mid-1970s though 2017, ^14^C dates pointed to earlier dates, either the ‘high’ chronology date range in the mid-later 17^th^ century BCE or a ‘compromise early’ date range in the mid-16^th^ century BCE [refs. 33, 52–54, 56, S40–S42, S62, S65–S68, S71]. Converting ^14^C age measurements into calendar age estimates requires use of the ^14^C calibration curve [refs. 1, S155]. The measurements on samples relevant to the Thera eruption, when compared with the various iterations of the northern hemisphere calibration curve, IntCal, published from 1986 through 2013, gave about the same answer. Most probability in the 17^th^ century BCE; some probability in the mid-16^th^ century BC. And more or less no probability after about 1530 BCE. While unacknowledged, this undoubtedly led to a little chronological ‘creep’. Scholarship supporting the conventional position found themselves adjusting the ~1500 BCE date, and instead advocating for dates around 1530 BCE or 1525 BCE, for no very good reason other than it was at the very margins of the ^14^C possibilities [refs. S156, S157]. Otherwise, those who wished to support the conventional date range had to try to reject or question the ^14^C evidence [refs. S69, S72, S134, S147, S156–S159]. Most common was to speculate about contamination from volcanic CO_2_, despite an absence of positive evidence for any of the samples dated from the Akrotiri excavations [ref. 33], and indeed indications that dates from sites well away from Thera nonetheless produced about the same ^14^C age [refs. 52, S68]. There seemed to be an intractable conflict. A side had to be chosen. The fundamental issue was whether ^14^C had, somehow, to conform to a pre-existing archaeological-cultural synthesis (or be rejected), or whether, instead, the ^14^C evidence, from both the Levant, Egypt and the Aegean instead indicated a need to re-think some of the long held assumptions and to write a different history [refs. 33, 52–54, S40, S66, S160, S161].

A dramatic change came with work reported in 2018. Rather than criticize the ^14^C dates from Thera, measurements of annual resolution known-age BCP wood samples indicated that the ^14^C calibration curve needed revision in the period from about 1660-1540 BCE [ref. 43]. Slightly older radiocarbon ages in general were identified across this interval, and, in particular, the increased resolution, combined with the somewhat older offset AA dates overall and BCP dates in particular (see Supplementary Discussion 1 above), enhanced (maximized) the 16^th^ century BCE wiggle and dating ambiguity, creating what we might term a maximal height wiggle (MHW) in this area of the calibration curve. The effect of these changes and the MHW was stated to be an increased probability for a 16^th^ century BCE date for the Thera eruption [ref. 43]—ref [43] regarded their new evidence as only ruling out dates after 1510 BCE (leaving the rest of the 16^th^ century BCE in play). This announcement was a surprise, and a potential game-changer. Several different groups rushed to test, replicate, and refine this finding. As of spring 2020, around 800 new high-precision AMS ^14^C dates have been run on known-age (and mostly single-year) tree-ring samples in the period 1700-1480 BCE in a major research push covering the period bracketing the possible dates of the Thera eruption [ref. 2]. The interval now has by far the most calibration information of any two century period in prehistory. All these new data are encapsulated in the new IntCal20 ^14^C calibration curve [ref. 1]. This slightly modifies the AA claims in ref. [43]. IntCal gives a new consensus curve (from all the data available) and a consensus scaling of the MHW in the earlier-mid 16^th^ century BC.

In light of the extraordinary body of new evidence, we return to the question of the absolute date.

*^14^C dating the Thera eruption 2020*

There are two principle ways to date the Thera eruption with ^14^C. The first route is to assess the dates available from plant material alive until shortly before the eruption on Thera itself. This comprises (i) samples on short-lived plant remains from the volcanic destruction level (VDL) at Akrotiri on Thera used in previous studies [refs. 33, 52–54, S67, S68], and (ii) an olive tree branch found buried in the volcanic pumice and argued to have been killed by the eruption—thus dating the outermost wood should give the approximate date of the eruption [refs. 54, 56]. A paper considering aspects of two of these options in light of the IntCal20 dataset has already appeared [ref. 2]. The second route is to add in additional samples from archaeological contexts in the Aegean from contexts securely before, contemporary with, and after the volcanic eruption and to use Bayesian chronological modelling techniques to constrain possible age ranges to help resolve overlaps and ambiguities [ref. 52]. We describe these approaches, data and modelling below. The modelling uses OxCal [refs. 22–24] and the IntCal20 calibration curve [ref. 1].

1. *Weighted average from short-lived plant remains from the Akrotiri VDL.*

This approach is considered by ref. [2] which employed an average ^14^C age of 3350±10 BP as representative of the Akrotiri VDL. Given the more multi-modal nature of IntCal20, thanks to the input of numerous dates on 1-year resolution samples, this leads to several possible calibrated calendar age possibilities. Ref. [2] found a most likely single range 1635–1612 BCE, but also several other probability regions in both the 17^th^ and 16^th^ centuries BCE. The clear majority of the probability lies before 1600 BCE (82.7% of the most likely 95.4% hpd = the 78.9% of overall probability that forms part of the 95.4% hpd range from 1602 BCE and earlier). We consider two other weighted average values: Supplementary Fig. S10. The first is the weighted average from the set of 25 dates on short-lived plant remains from the Akrotiri VDL from the Oxford (OxA), Copenhagen (K) and Vienna (VERA) laboratories [refs. 33, 52, 54] with IntCal20 (Supplementary Fig. S10a) (the three highly varying Hd data in ref. [52] are excluded). The second is the 13 date-date sub-set (from the OxA and VERA laboratories) run in the AD2000s (Supplementary Fig. S10b). The results are largely similar. We then further consider the effect of a hypothetical +8 ^14^C year offset (Supplementary Fig. 10c, d). We do this as it is unclear whether all or any Aegean-region growing-season offset is already adequately incorporated in IntCal20 because of the added-age from the AA IrO and especially BCP ^14^C data forming part of IntCal20 (see Supplementary Discussion 1), and because the AA GOR dataset perhaps indicates an additional ~5 to 8 ^14^C year offset in the 1600–1540 BCE interval (see Supplementary Discussion 1). We suspect this is very much a ‘maximum-case’ in this instance. However, it usefully highlights the critical issue of clarifying and resolving, robustly, the absence/presence of a regional ^14^C offset 1700–1500 BCE. There is a very fine (small) distinction between the later 17^th^ century BCE and the earlier-mid 16^th^ century BCE with the IntCal20 record. Resolution of the Thera date is thus very susceptible to even small changes.

(ii) *A Tau_Boundary model*

This is the approach proposed as most appropriate in ref. [53] and used also in refs. [33, 54]. The set of Akrotiri VDL ^14^C dates on short-lived samples are considered as a Phase in OxCal with a distribution ramped exponentially towards the end of the Phase—the volcanic destruction. A Boundary at the end of the Phase thus offers the estimate of the eruption date. This is achieved by pairing a Tau_Boundary with a Boundary in OxCal [ref. 23]. This approach has the advantage that it does not assume all the evidence is exactly contemporary and any older evidence (for whatever reason) is not overly influential. This approach should give about the latest plausible date range for the eruption. We consider the set of 24 dates on short-lived material from the Akrotiri VDL used by ref. [53] and we add also the date published subsequently on insect chitin from a storage jar from the VDL (charred bean weevils, *B. Rufipes* from pithos 1, one of several large storage jars found in room 5 on the south wall of the ground floor of the West House at Akrotiri [ref. S124]): see Fig. 5a. The OxCal runfile is in Supplementary Table S3. We consider also the possible effects of a small 8 ^14^C years offset (see above): see Fig. 5b. The calendar age range in Fig. 5a at 68.3% hpd is 1621–1608 BCE, and 1625–1588 BCE (88.4%), 1572–1553 (5.0%) and 1552–1542 BCE (2.0%) at 95.4% hpd. This approach more clearly identifies a most likely date range for the Thera eruption in the late 17^th^ century BCE to the start of the 16^th^ century BCE. As shown in Fig. 5b, a small ^14^C offset—if present—does not fundamentally change the dating range, but gives (progressively) more probability in the earlier-mid 16^th^ century BCE.

(iii) *Thera olive branch*

There are four published ^14^C data for, in order, the innermost to the outermost dated segments of an olive branch assumed killed by the eruption [refs. 2, 33, 54, 56]. Ref. [2] considered these data. Supplementary Fig. S11a shows the calendar fit of an OxCal [ref. 23] Sequence of these ^14^C dates with the order from inner (oldest) to outer (most recent) segments using OxCal and IntCal20 [ref. 1]. For the data and OxCal code, see Supplementary Table S3. Originally, it was argued that growth increments could be recognized in this sample [ref. 56]. Subsequent critical discussion holds that growth increments cannot be reliably identified in most olive wood [ref. 55]. Thus there is merely an ordered sequence (as considered in [ref. 54]), and the last date is a *terminus post quem* (TPQ) for the eruption. A further complication was raised recently. It was shown that wood extracted from different locations around the outer circumference of olive cross-sections can yield different ^14^C ages, some contemporary with the correct age, but some older by as much as several decades [ref. S162]. However, the relationship does not appear random. Instead, contemporary ages are associated with the lobes and active growth regions of the stem [see ref. [10], supplementary material]. Examination of the illustrations of the Santorini olive branch section and the portions sampled for the ^14^C dates indicates that this represents an ordered sequence of dates out to a growth lobe [ref. 56]. Thus there is a reasonable expectation that the date of the outer portion of this growth lobe indicates the approximate time the branch ceased growing. It is clear that the ordered sequence of four ^14^C dates can only fit on the IntCal20 curve in the area indicated in Supplemental Fig. S10a (see also [ref. 2]). The last dated segment most likely dates 1625-1597 BCE (70.2% hpd) (Supplemental Fig. S11b). However, 25.2% hpd falls in the earlier-mid 16^th^ century BC. Thus again, while a date in the last decades of the 17^th^ century BCE is most likely, about a quarter of the total probability indicates the earlier-mid 16^th^ century BCE. But not later. Even an arbitrary addition of 8 ^14^C years (1‰) makes little difference in this case (Supplemental Fig. S11c).

(iv) *Aegean Bayesian model from samples and ^14^C dates from before, contemporary with, and after the Thera eruption*

This approach is a revision and up-date of the approach employed in ref. [52]. A Bayesian model is constructed comprising, in order, ^14^C dates on samples known to date before the Thera eruption period (before mature LM IA), samples from immediately before the time of the Thera eruption during mature LM IA, samples from the time of the VDL or contemporary contexts in late/end LM IA, and then samples from contexts and archaeological periods known to be after the eruption (LM IB through LM II).We did not use the dates excluded in ref. [52]. A pair of Boundaries, one after a Tau_Boundary and the set of ^14^C dates on the short-lived samples from the Akrotiri VDL (combining those in (i) and (ii) above), and one after a Phase including all the other immediate TPQ or contemporary dates for the eruption, which are cross-referenced, provide the date estimate for the Thera eruption as constrained by all the prior, contemporary and subsequent information. The model uses the data in ref. [52] with additions. The Akrotiri VDL set comprises the 25 dates used in (i) – that is excluding the three Hd dates in ref. [52] which vary substantially – and the additional date reported since on insect chitin [ref. S107]. The other dates, approaches and contexts added to the model in ref. [52] come from refs. [53, 54, 56, S109, S110, S124, S160, S164–S168]. In particular, as in (ii) above, a Tau_Boundary paired with a Boundary is used to model an end Boundary and approximate date estimate for each of the destruction contexts in the model. For the model and a discussion of its construction and logic/assumptions, see Supplementary Table S3. The full model is shown in Supplementary Fig. S12. The modelled Thera eruption date is shown in Figs. 6a and Supplementary Fig. S13. The modelled calendar age ranges at 68.3% hpd and 95.4% hpd for a selection of elements in the model as shown in Supplementary Fig. S12 are listed in Supplementary Table S4. We note one issue. There are two ^14^C dates on a small piece of charcoal from the Pelekita Cave found above the deposit of Theran tephra [ref. S95]. The locus is described as “just above the tephra layer” in ref. [S95]. The interpretation is that the charcoal is “anthropogenic in origin, in terms of deposition. Considering its stratigraphic position, the charcoal piece probably reflects renewed usage of the cave by humans after the Minoan Santorini eruption” [ref. S95]. It might seem that this charcoal offers a *terminus ante quem* (TAQ) for the eruption. However, ref. [S95] observes that the ^14^C ages are older than the average age for the Akrotiri VDL, and so raises the possibility that “the charcoal consists of wood that already grew before the eruption, but was used by people to make a fire in the cave after the eruption (old-wood effect)”. In view of the indications for some rather older east Mediterranean-Anatolian ^14^C ages through much of the 16^th^ century BC, based on the AA GOR dataset [ref. 38], Fig. 3e, there is in fact no necessary ^14^C reason to have to assume that this charcoal sample pre-dates the eruption (given allowance for laboratory error and inter-laboratory variation especially). It could be both correctly dated and from after the eruption and so a useful additional constraint. However, since there is doubt here, we consider the primary model without these two dates as a TAQ for the eruption. Supplementary Fig. S16a shows the dating probability for the Thera eruption as in Fig. 6a if in fact the two Pelekita Cave dates are included as an eruption TAQ (A_model_ ~131, A_overall_ ~140), and Supplementary Fig. S16b shows the dating probability for the Thera eruption as in Fig. 6b (with the hypothetical additional +8 ^14^C years adjustment) if in fact the two Pelekita Cave dates are included as an eruption TAQ (A_model_ ~148, A_overall_ ~148). The differences observed are that the Pelekita Cave TAQ reduces the probability and extent of the mid-16^th^ century BCE range for the Thera eruption estimate a little in each case. Thus in Supplementary Fig. S16a the 95.4% hpd range ends 1558 BCE versus 1545 BCE in Fig. 6a, and in Supplementary Fig. S16b the 95.4% ranges ends 1547 BCE versus 1538 BCE.

We also consider an alternative model. It has been suggested in the past that perhaps volcanic CO_2_ affected some or all of the ^14^C dates from Thera (e.g. ([refs. S157–S159]), despite no positive evidence for this among any of the dates on archaeological samples and despite similar ^14^C ages being measured on samples from late LM IA contexts from loci well away from Thera and so removed from any putative volcanic CO_2_ effect [refs. 33, 52, 54, 56]. If there *were* a volcanic (i.e. depleted) CO_2_ effect, then the ^14^C ages would be made older (likely much older) [ref. 33]. Hence those raising the volcanic CO_2_ issue argued that this might be why the ^14^C dates from Thera indicated a higher chronology than the conventional age. For this reason, we re-ran the Supplementary Fig. S12 model but excluding all the ^14^C dates from Thera (see Supplementary Table S3). The modelled age for the Thera eruption Boundary from this ‘no-Thera-data-model’ using unmodified IntCal20 is shown in Supplementary Fig. S15a. This model in fact more clearly indicates a later 17^th^ century BCE date than the with-Thera-data model, but otherwise yields a very similar (but less constrained) age estimate for the Thera eruption. This finding demonstrates that we may conclude that volcanic CO_2_ is therefore not causing any observable effect on the samples from Thera. We accordingly regard the modelled age in Fig. 6a, using all available data, as our best current estimate for the date of the Thera eruption using IntCal20. Supplementary Fig. 15b re-runs the Supplementary Fig. 15a model but allowing for a hypothetical additional +8 ^14^C years. Again a late 17^th^ century BCE date range is most likely (64.1% of the 68.3% hpd range), although there is now slightly increased probability in the earlier to mid-16^th^ century BCE.

The date for the Thera eruption (Fig. 6a) is modelled as 1617–1602 BCE (64.7%) and 1567–1565 BCE (3.5%) (68.3% hpd) and 1619–1596 BCE (72.6%) BCE and 1576–1545 BCE (22.9%) BCE (95.4% hpd). This is the modelled age from one run of the model (the run with the median A_model_ value out of the 11 model runs as shown in Supplementary Fig. S12). Supplementary Fig. S13 illustrates the modelled Thera eruption age from this example and also 10 other runs of the model to give an indication of the range among the model runs. There are only small variations between each model run and the 11 examples are all very similar. Eight of the 11 model runs have some 68.3% hpd range somewhere 1567–1562 BCE, and three model runs have all the 68.3% hpd range 1617–1601 BCE. All the 95.4% hpd ranges are very similar, with a most likely range across the 11 runs of 1619–1596 BCE with 65.8% to 74.5% hpd and a less likely range across the 11 runs of 1577–1543 BCE with 20.9% to 29.6% hpd. We use the probabilities and age ranges indicated in Fig. 6a from the median run in the main text—the possible range of variation (and a measure of the robustness of the findings) can be gauged from Supplemental Fig. S13. The incorporation of the additional constraints has—as anticipated and the benefit of Bayesian Chronological Modelling [refs. 23, 52, S169]—narrowed the date ranges obtained in (i) to (iii) above. Across the 11 model runs, the 95.4% hpd range ends 1543 BCE (range 1545–1543 BCE), and this represents the latest even plausible date for the eruption. This rules out the conventional or ‘low’ chronologies and their synthesis of Aegean and east Mediterranean prehistory at this time. Instead, only the ‘compromise high chronology’ (a Thera eruption date in the earlier-mid 16^th^ century BCE) [ref. S40] or the ‘high’ chronology (a Thera eruption date in the late 17^th^ century BCE or start of the 16^th^ century BCE) is possible. The latter is clearly the more likely.

The issues of possible offsets, between laboratory data, and accounting for possible differences in typical growing seasons, is discussed in the main text and in Supplementary Discussion 1 above. In the case of the data from the southern Aegean (from various trees species as wood-charcoal and from agricultural crops), the relevant traditional growing season likely covers late winter through earlier summer (depending on crop), for example (for the agricultural crops) based on comparison with other similar southern Aegean islands like Karpathos and Amorgos and information from Crete [refs. S170–S172], and as compatible with the available information on the pre-eruption Theran countryside and its vegetation [refs. S131, S173]. This means only a likely small to modest possible growing season ^14^C offset for pre-eruption Theran plants (and herbivores eating these) versus the source regions of the IntCal20 wood across this period—much smaller than possible or typical for the cases of Egypt [refs. 6, 7] or the southern Levant [ref. 8] where there is a larger offset in growing season timings (which include the winter and end around the start of the summer)—versus central and northern Europe. There were of course likely variations around any average relationship [ref. 10] (Fig. 3). As discussed in Supplementary Discussion 1, in the interval 1700–1500 BCE it is evident that the effect of the AA BCP data (especially) and the AA IrO data to some extent, has been to slightly inflate, and so pre-‘Mediterranean-ise’ the IntCal20 curve (a notable difference compared to the situation with the previous IntCal13 calibration dataset [ref. 10]). Thus the calendar age ranges and probabilities achieved in (i) to (iv) above without any additional adjustment should be either (a) approximately representative/appropriate for the Aegean and Thera data, or potentially even be (b) slightly shifted to too recent dates where there is not a positive ^14^C offset between the east Mediterranean and IntCal (and so central and northern Europe and North America) (see main text, [ref. 10]). Therefore, these dating estimates for the date of the Thera eruption should in fact be either appropriate, or minima. This especially applies to the Hd data for the Thera olive branch, since these dates, obtained by LLGPC [ref. 56], likely need an adjustment to slightly older ^14^C ages when calibrated against an AMS ^14^C dominated calibration curve. The dates from the Demokritos laboratory (DEM dates in the Supplementary Fig. 10 model) are also non-AMS ^14^C technology [refs. S164, S168].

The AA GOR data, however, raise the issue of whether there might, nonetheless, be a small additional offset applicable in the east Mediterranean-Anatolian region in the period 1700–1500 BCE and especially during the period of the reversal and plateau ~1600–1540 BCE (see main text, Supplementary Discussion 1). As discussed in the main text and Supplementary Discussion 1, we have therefore considered (but do not support) the case of an additional +8 ^14^C year offset (as an approximate maximum possible case). We thus re-ran the Aegean model in Supplementary Fig. S12 with a +8 ^14^C year offset (Delta_R of 8,0 in OxCal). The alternative dating probability for the Thera eruption is shown in Fig. 6b (compared to Fig. 6a using IntCal un-modified). There is a marked difference. The un-modified IntCal20 model strongly favours the late/end 17^th^ century BCE. The +8 ^14^C years offset model in contrast strongly favours the earlier-mid 16^th^ century BCE. A small change, therefore, could make a large difference in this case. We also consider the no-Thera-data model with a +8 ^14^C years offset in Supplementary Fig. 15b and the model including the Pelekita Cave TAQ with a +8 ^14^C years offset in Supplementary Fig. 16b.

However, we argue that an average +8 ^14^C years adjustment is in fact likely too large as an approximate ‘maximum’ offset consideration (see Supplementary Discussion 1, Supplementary Fig. S14). As discussed in the main text, with the revision of IntCal20 with numerous modern AMS ^14^C dates, and comparing measurements by AMS ^14^C with sections of this revised calibration curve where there has been substantial new input of AMS ^14^C dates (thus contrast the situation discussed for the MBA chronology in the main text and as shown in Fig. 1), instances of apparent seasonal (growing season) offsets have reduced when compared to the previous situation with IntCal13. Offsets that were in the ~19–21 ^14^C year range have reduced to ~12±5 ^14^C years (this is a major change versus the situation with IntCal13 and revises assessments in e.g. refs. [6, 8, 10]). These offsets were for likely maximum offsets cases for the mid-latitudes of the NH (Egypt and the southern Levant where the typical growing season is almost the opposite of that for central-northern Europe and North America). The potential growing season offset for the Aegean-Anatolian region should be smaller (with variations within the Aegean-Anatolian region in local climate and environment ranging from relatively similar to central-northern Europe—hence likely no real offset—to more ‘Mediterranean’ with some offset). The estimate of up to ~8 ^14^C years for the Aegean case as the ‘maximum’ came as towards the plausible maximum from several comparisons and considerations of adjustments for the AA GOR dataset (see Supplementary Discussion 1) indicating perhaps (i) a ~3.7±2.7 to 8.3±2.6 ^14^C years general offset or (ii) a specific offset 1600–1540 BCE from 1.5±4.0 to 10.0±3.7 ^14^C years depending on whether and how much of the noise/outliers are removed from the AA GOR dataset (see Supplementary Discussion 1), or (iii) a consideration that if we adjust the AA GOR data by the difference between parallel AA IrO and ETH IrO (6.2±1.8 ^14^C years [ref. 39]), and remove those data over 2σ from the 10 point weighted adjacent average, this becomes a positive offset across the period 1600–1540 BCE of ~7.8±3.8 ^14^C years (Supplementary Fig. S6) – or, if we use the analysis in Supplementary Fig. S14 this would be a positive offset of either 5.2±4.8 ^14^C years or 5.6±5.2 ^14^C years for 1600–1540 BCE, and (iv) going to no more than about half to two-thirds of the likely mid-latitude maximum seasonal (growing season) offset around 12 ^14^C years (main text). Clearly this is all rather approximate and imperfect. Therefore, one major outcome of the present study is the need to robustly define the seasonal (growing season) offsets for the Aegean (and the wider Mediterranean) comparing data all measured under comparable conditions. For the present, we regard the ‘cleaned’ AA GOR dataset shown in Supplementary Fig. S14 as likely a best estimate, and hence the likely real AA GOR offset versus IntCal20 (allowing for the observed inter-laboratory AA offset of either +5.8±2.7 ^14^C years versus IntCal20 based on AA IrO or +6.2±1.8 ^14^C years based on AA IrO versus ETH IrO), as small to negligible overall (respectively 4.1±3.4 ^14^C years or 3.7±2.7 ^14^C years), and only very slightly larger (and still small to negligible) for the period 1600–1540 BCE at respectively 5.6±5.2 ^14^C years or 5.2±4.8 ^14^C years.

*Discussion*

Thirty years ago the dating probabilities for the Thera eruption, between a 17^th^ century BCE range, and a mid-16^th^ century BCE range, were assessed at about 70:30 [ref. S174]. The possible ranges at 68% confidence were stated to be 1674–1606 BCE versus 1554–1534 BCE. This was based on a smaller dataset from Thera, and a much less intensely defined calibration record. Today, the big picture is little changed in macro terms—just refined or narrowed substantially. We still have the same possible ambiguity. However, the major difference is that the possible ranges are now substantially narrower. The more likely 17^th^ century BCE dating range is circumscribed to the last few decades of the 17^th^ century BCE. The earlier-mid 16^th^ century BCE range rules out most of the later 16^th^ century BC. The large dataset analysis in Fig 6a and Supplementary Fig. S13 limits the total dating window at 95.4% hpd to 1619–1543 BCE, a 77-year inclusive period. It points especially to the possible regions of 1617–1601 BCE (average 62.8% hpd) and 1570–1562 BCE (average 5.4% hpd). The two big changes IntCal20 brings are thus, first, to push the 17^th^ century BCE range down to the very last decades of the century, and, second, the MHW highlights a wiggle and region close to and following 1570 BCE (and perhaps to ~1560 BCE) as the less likely alternative.

The major area of uncertainty now is the question of whether or not an additional small ^14^C offset may apply in this period. Several indications suggest no, but the AA GOR dataset may suggest yes (main text, Supplemental Discussion 1, see above)—but this dataset is rather less satisfactory with considerable noise and several large outlying values. As illustrated, even a small offset makes a fundamental difference in this case. This is therefore a topic needing clarification and robust resolution.

The revised Thera date ranges (Fig. 6a and b) bring a new twist to the question of possible relationships with likely volcanic signals noted in various proxy archives. Further, the up-scaling of the eruption’s impact [refs. S36, S80, S122, S123] makes a recognized signal in the ice-core records more plausible. In particular, we can consider the recent tree-ring calibrated re-dating of the Greenland ice-core record of major volcanism and the common inter-core tie-points [refs. 68, S175] (Fig. 6). Several changes are immediately called for when compared with previous discussions. The 1653 BCE volcanic signal (ice-core and matching frost-damaged ring in the BCP record [refs. 68, 69]) may now be discounted as irrelevant to Thera. Similarly, the Porsuk RY854 growth anomaly previously associated with the Thera eruption [ref. S153] is dated 1667–1646 BCE (68.3% hpd) and 1677–1643 BCE (95.4% hpd) with IntCal20 (re-running model 6a from ref. [18], A_model_= ~96, A_overall_= ~119). Thus the cause of this growth anomaly is also clearly distinct from the date of the Thera eruption (but if a volcanic origin is sought it could of course link with the 1653 BCE ice-core signal of ref. [68]).

A similar assessment applies to a BCP ring-width minima noted at 1649 BCE [ref. 69]. The much-discussed 1627 BCE ice-core signal and likely corresponding 1627 BCE frost damage record in BCP, and widely attested Northern Hemisphere tree-ring growth anomalies 1628–1626 BCE [refs. 68, 69, S61, S63, S64, S176, S177], long thought to be a Thera candidate, is also now unlikely from the ^14^C evidence (an important IntCal20 change). 1627 BCE can therefore likely be ruled out as associated with the Thera eruption with IntCal20. Furthermore, this eruption appears most likely to represent a volcano other than Thera based on analysis of the associated tephra, and is perhaps Aniakchak II [refs. S74, S154, S178].

Using non-modified IntCal20 (Figs. 5a, 6a), possible Thera eruption associations begin especially with the tree-ring minima noted 1619–1617 BCE in Finnish and Yamal series [ref. 69] which are on the upper edge of the new plausible Thera eruption date range. 1617 BCE, in particular, falls just inside the most likely 68.3% hpd range (Fig. 6a). In contrast, whereas previously an ice-core volcanic signal at 1610 BCE had seemed too late for the likely Thera eruption ^14^C range [ref. 68], it now lies right in the middle of the most likely dating range. This signal could also correspond with the likely volcanic signal in the Sofular Cave speleothem suggested to be associated with Thera [ref. S152]. Thus it forms a prime candidate for further investigation. A BCP growth minimum noted for 1597 BCE [ref. 69] is possible, but there is no major volcanic signal in the ice-cores. A volcanic ice-core signal at 1586 BCE [ref. 68] falls more or less in the gap between the 95.4% hpd ranges in Fig. 6a, but falls inside the range in the Fig. 5a analysis. It is close enough to deserve investigation, but seems less than likely. Tree-ring growth anomalies are noted at 1584 BCE and 1579 BCE in Finnish tree series, for which a volcanic origin is suggested [ref. 70]. The former misses the Fig. 6a range but could match Fig. 5a, while the latter fits within the 95.4% hpd ranges in Figs 5a, 6a. Again this period would be worth investigation. The only other instance in the set of volcanic tie-points [ref. S175] likely (on revision for dates following ref. [68]) to lie before 1540 BCE, and so be of possible relevance to Thera in Fig. 6a, is at ~1560 BCE—and this interval also deserves attention [ref. 38]. Growth minima, but not frost damage (which in particular appears associated with major volcanism [refs. 68, S61]), are reported in the BCP record for 1544 BCE and 1524 BCE [ref. 69]. The former makes the very outer extremity of the 95.4% hpd range. The latter is now well outside the possible range and may be discounted despite suggestions to associate it with Thera [ref. S179]. Ref. [38] states that “Unusually narrow growth or frost-damaged cells” are recorded in BCP for 1560 BCE and 1546 BCE. The reference cited, [ref. 69], does not list these supposed years—thus it unclear whether these are subsequently recognized instances of frost damage or growth minima, or if this is an error. 1560 BCE is already noted above. 1546 BCE, like 1544 BCE mentioned above, is within the latest part of the 95.4% ranges and should be checked.

Using the hypothetical +8 ^14^C years adjusted model (Figs. 5b, 6b), would mean we can rule out all possible dates before 1610 BCE. The 1610 BCE eruption falls within the 95.4% range as do the dates mentioned above at 1597 BCE, 1584 BCE, 1579 BCE, 1560 BCE, 1546 BCE, 1544 BCE and, in addition, so does the signal at 1539 BC. The Fig. 6b model would especially favour (within the 68.3% hpd range) the dates 1560 BCE, 1546 BCE and 1544 BCE. Only the first is a volcanic signal in the Greenland ice core record (which now seems a likely expectation for the Minoan eruption of Thera in view of the reassessment of its scale and volatile emissions: refs. S36, S80, S123).

The search for a specific Thera eruption signature in ice-cores, where volcanic tephra could offer positive support, is thus changed, and much narrowed, applying the IntCal20 calibration. To achieve a precise geological marker that can be related against absolute or near-absolute archives, like tree-rings, ice-cores or (some) speleothems, or used to date less secure records like sediment cores, a specific absolute date is necessary. As yet, for the Minoan eruption of Thera, this will require further work. However, the possible date range, as much narrowed from IntCal20, already brings sufficient clarity to resolve a long-running controversy in archaeology and history. The Thera eruption date is before ~1543/1538 BCE at a minimum considering either of Fig. 6a or Fig. 6b. The question of a small regional ^14^C offset moves the dating probability within the dating range, but does not much change the latest plausible date. This rules out the conventional or ‘low’ chronology (dates ~1530-1500 BCE), and so provides key coordination for historical synthesis in the region.

Based on assessments of both the historical evidence and ^14^C (in a period largely unaffected by the recent changes to IntCal), the Egyptian New Kingdom (18^th^ Dynasty) begins in the mid-16^th^ century BCE [refs. 7, 33–36, S141–S143], for example somewhere between ~1565 BCE (‘high’) to ~1540 BCE (‘low’) [ref. S141]. This is when Ahmose successfully reconquers Egypt, driving out the Hyksos (and all but eradicating them from subsequent history) [refs. S180–S184]. Before this, the Hyksos had ruled in the Nile Delta at the heart of a Levantine and east Mediterranean trading system for well over a century. Their capital, Avaris (the archaeological site of Tell el-Dab‘a), was the super-site of SW Asia and the Mediterranean during this period and the center of a maritime trade system [refs. S182, S185, S186]. Under the previous conventional chronology, much of the LM IA period and all the LM IB period were contemporary with Egypt’s resurgent 18^th^ Dynasty regime and its conquest and ensuing domination of the SE Mediterranean from Ahmose onwards. Indeed, the start of the LM I period was originally placed to indicate a parallel progression following the change to the New Kingdom in Egypt [refs. S187, S188].

In contrast, with either an earlier-mid 16^th^ century BCE date for the Thera eruption, or a late 17^th^ century BCE date, the long LM IA period, the apogee of the New Palace period and of Cretan prominence and influence in the Aegean and wider Mediterranean [refs. S38, S49, S57, S129, S130, S165, S189–S193], is instead contemporary with the very different and preceding world of the Hyksos. This is a marked difference [refs. S40, S161], with regard to trade and cultural connections and influences evident across material culture, art, language and religion in the earlier Cretan New Palace period. Only part to most of the subsequent LM IB period would lie contemporary with the 18^th^ Dynasty. This distinction and the correct cultural synchronization is important to a wider historical reconstruction, and it has long been recognized that the Thera eruption date is pivotal:

“Why is it important to fix the date of the Minoan eruption of Santorini? Essentially the answer is that in order to write the history of international relations of the later Middle and the Late Bronze Age in the eastern Mediterranean we need to establish whether, at the time of the Minoan eruption of Santorini, the Egypt which was linked to the Aegean, Cyprus and the Levantine region was that of late Dyn. XIII or earlier Second Intermediate Period on the one hand or that of the early New Kingdom (early Dyn. XVIII) on the other.” (ref. [S156], quoted also by ref. [S160])

We now have an answer from IntCal20. It was the Egypt of late Dynasty 13 and the Second Intermediate Period, and the group (as referred to by the Egyptians) we call the Hyksos from the period before Ahmosis and the 18^th^ Dynasty [refs. S180–S185]. Those advocating the ‘high’ chronology have repeatedly observed this point [refs. 33, 52–54, S40, S161], but, now that the much refined IntCal20 calibration curve confirms that the ‘low’ chronology is not possible—whether unmodified or even with a hypothetical small regional ^14^C offset in operation—at last the correct history can be addressed and the important Hyksos period—which has long failed to receive due attention—can receive the historical focus it merits.

The overall dating model (Supplementary Fig. S12, Table S4) confirms that the LM IA period was long, as also the subsequent LM IB period. Evidence from both archaeology and ^14^C has for some time indicated that the LM IB period, formerly considered short [refs. S40, S188), was in fact of much longer duration [refs. S137, S165, S167, S193]. The dates for the close of the LM IB period, and the transition to LM II, in the earlier through mid-15^th^ century BCE work well with the known Egyptian connections. It is a few mature LM IB (and its mainland contemporary LH IIA) products, and not LM IA, that are found in earlier 18^th^ Dynasty contexts through the reign of Tuthmosis III. Based on comparisons of decorative motifs shown worn by the Keftiu in wall-paintings from some elite Egyptian tombs—thought to represent people from Crete—and Aegean ceramic designs, it has been generally agreed that the LM II period (at least) must begin by later in the reign of Tuthmosis III [refs. S40, S53, S59, S134, S135, S139, S144, S146, S149, S165, S194, S195]. His nearly 54-year reign ends somewhere ~1450-1425 BC, depending on various Egyptian historical chronologies, entirely consistent with the Aegean ^14^C evidence.

IntCal20 has not completely solved the Thera dating debate. An exact date remains to be identified. However, it has usefully narrowed the possible date range and effectively resolved some previous questions. A few targets for where to look in Greenland ice-cores and other archives are identified. For the archaeology of the eastern Mediterranean, the possible IntCal20 date range concludes the long-running ‘high’ v. ‘low’ chronology divide. The ‘low’ (or conventional) chronology is no longer possible. The plausible late 17^th^ century BCE or early-mid 16^th^ century BCE ranges both require a new and broadly similar historical synthesis. We move to refinement, and leave behind a past state of uncertainty.

The question of the association with the LM IB destructions is also clarified, but not resolved. At the extreme minimum there is a period of several decades intervening, and in fact there is likely a period more of ~75–100 years (earlier-mid 16^th^ century BCE eruption) to perhaps even 100–150 years (late 17^th^ century BCE eruption). Any direct and causal link appears loose at best. Arguments for severe economic and related dislocation post-eruption in LM IB on Crete [ref. S38], leading to decline and collapse, have cogency over a period of years to a few decades, but perhaps loose effect as the temporal interval moves into subsequent generations and to around 50 years and beyond? The eruption may rather be part of explaining the changed nature of the southern Aegean as the LM IB period begins, and be part of the reason for wider restructurings that occur through the course of this long period across the Aegean reflecting changed social, economic and political connections that in turn provide new opportunities and historical trajectories—for example for developing centres in southern Greece. Deconvolved, the search for direct Thera eruption impacts on Crete should be directed to the close of LM IA. As long noted, there is evidence here for changes and some destructions that might correspond [refs. S38, S56, S58, S109, S110, S113]. The much later LM IB destructions on Crete are instead selective and focused in a way that indicates organized human agency [ref. S196].

**
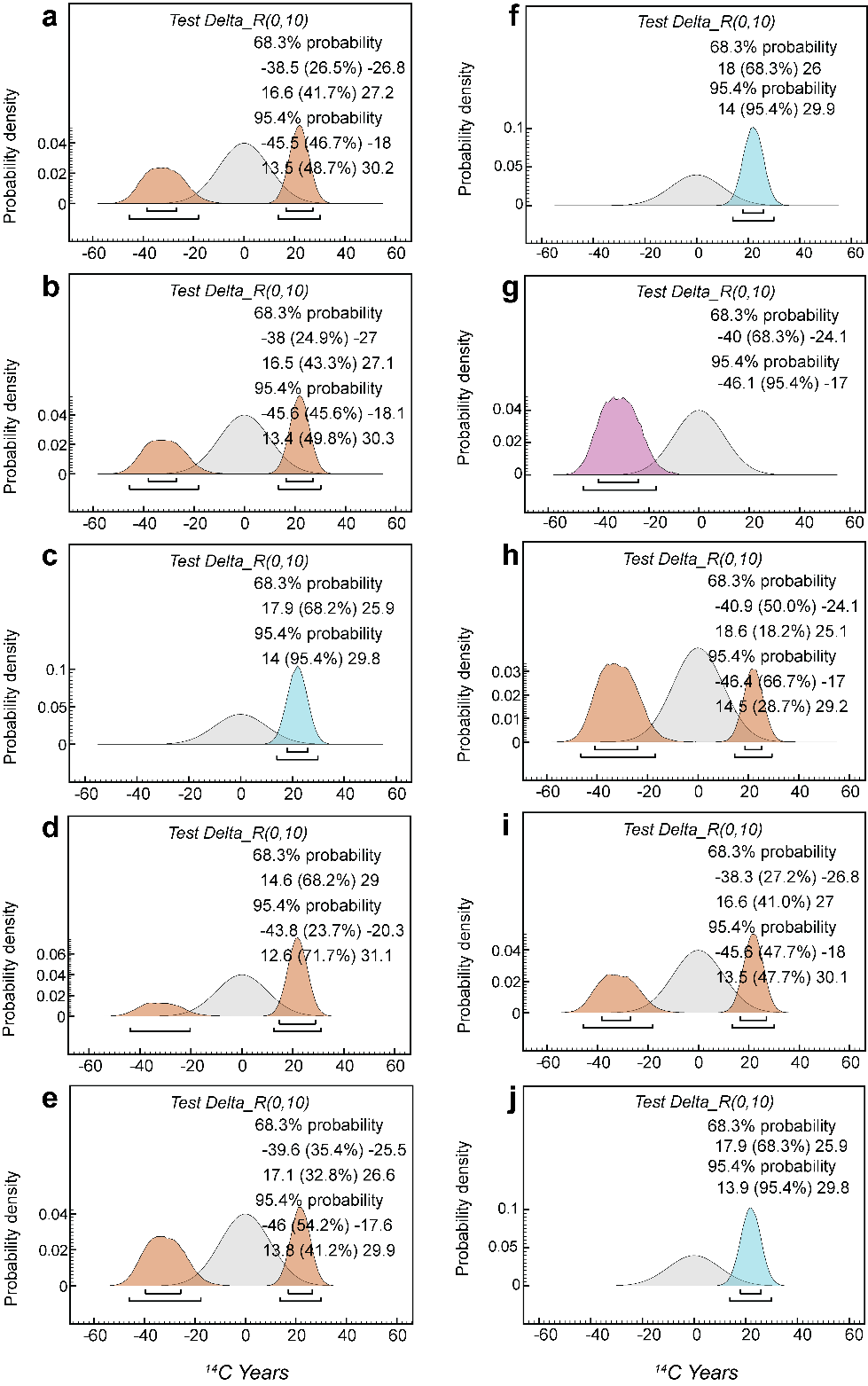
**

**Supplementary Fig. S1.** Systematic ^14^C offset test for the dataset and wiggle-match in Fig. S1a using a neutral prior of 0±10 ^14^C years and the OxCal ΔR function [ref. 24]. IntCal20 [ref. 1] is used with resolution set at 1 year. In several instances there is a bimodal outcome and poor Convergence (<95), with alternative ranges either ~22±5 ^14^C years or -32±8 ^14^C years (the 6 orange shaded posterior density plots). In some cases there is a resolution with good Convergence for an offset of ~22±4 ^14^C years (the 3 cyan shaded plots). Occasionally there is resolution with good Convergence for the alternative of -32±8 ^14^C years (the 1 magenta shaded plot). Overall, most probability favours the ~22±5 ^14^C years offset.

**
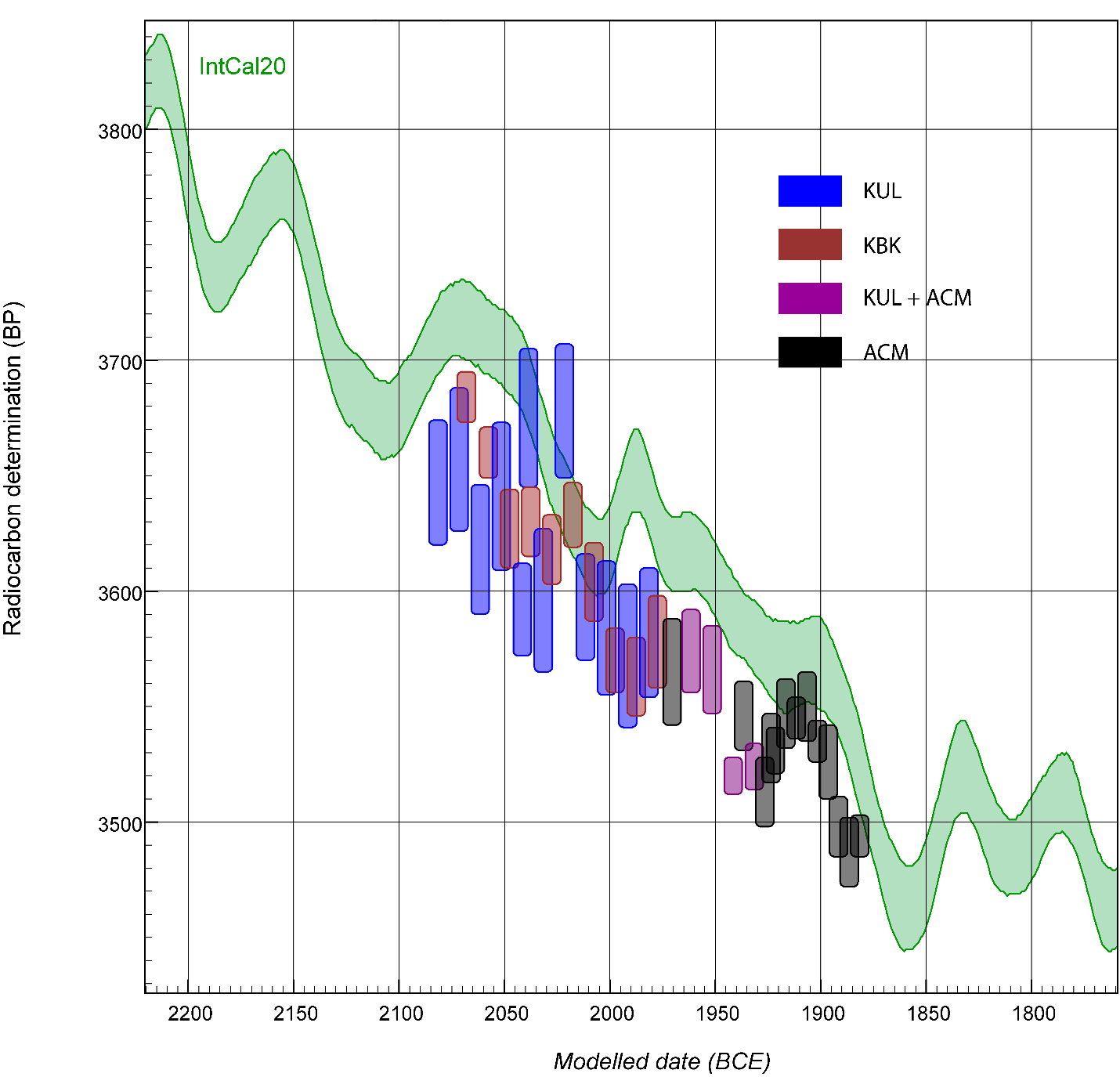
**

**Supplementary Fig. S2.** The wiggle-match of the MBA dataset (Fig. 1a) using an OxCal ΔR value of -32±8 ^14^C years (the less likely of the two options apparent in the analysis summarized in Supplementary Fig. S1) versus IntCal20 (±1σ). There is a poor visual fit. Nearly all the data do not match (lie on) the calibration curve. Instead, they lie below the calibration curve, and apparent structure, like the ‘wiggle’ represented in the ACM data, fail to correspond with the calibration curve. OxCal 4.4.1, IntCal20, curve resolution set at 1 year.





**Supplementary Fig. S3.** The all-data set of individual ^14^C measurements on the Kültepe (KUL), Karahöyük (KBK) and Acemhöyük (ACM) samples (n = 76) (data from published work [ref. 18] and Supplementary Table S1) placed according to the Fig. 1b fit. The 4 dates with an X marking are excluded from the Fig. 1b model. Three are the least compatible (flagged as outliers by the OxCal SSimple Outlier model [ref. 24]) in weighted averages which otherwise fail a χ^2^ test (see Supplementary Table S3) [ref. 42]. The fourth date (OxA-30907) [ref. 18] recorded a large difference between the δ^13^C value in the AMS versus independent MS. This is regarded as a reason to exclude it. OxA-30908 is also indicated (see Methods) – this is by far the largest outlier remaining in the dataset used for the Fig. 1b wiggle-match. It is part of the RY631 weighted average (which nonetheless passes a χ^2^ test at the 5% level [ref. 42]) but has an ~64% outlier probability with the SSimple Outlier model [ref. 24]. Removal does not change the wiggle-match placement. We note one other alternative: we consider the 75 data (excluding only OxA-30907 with a likely technical/sample issue) separately (i.e., no combining of data, in case this might be thought to bias). With a neutral ΔR test of 0±10 ^14^C years, this ‘separates’ dataset indicates a best fit wiggle-match only +2 years earlier than shown here or in Fig. 1 with an offset of ~26 ^14^C years (σ values all <4). If the same no-combinations model is then re-run with a ΔR of 26±4 ^14^C years, the placement is just +1 year from that found in Fig.1b and as shown above. This alternative thus makes no substantive difference to the fit used in the main text (Fig. 1b) or the historical associations observed in Fig. 2. Data from OxCal 4.4.1 and IntCal20, curve resolution 1 year.

**

Supplementary Fig. S4.** The MBA time series plotted against IntCal20 as in Fig. 1b but showing also a set of ETH ^14^C measurements on dendrochronologically dated (known age) single-year samples from Erstein, France (Supplementary Table S2).

**
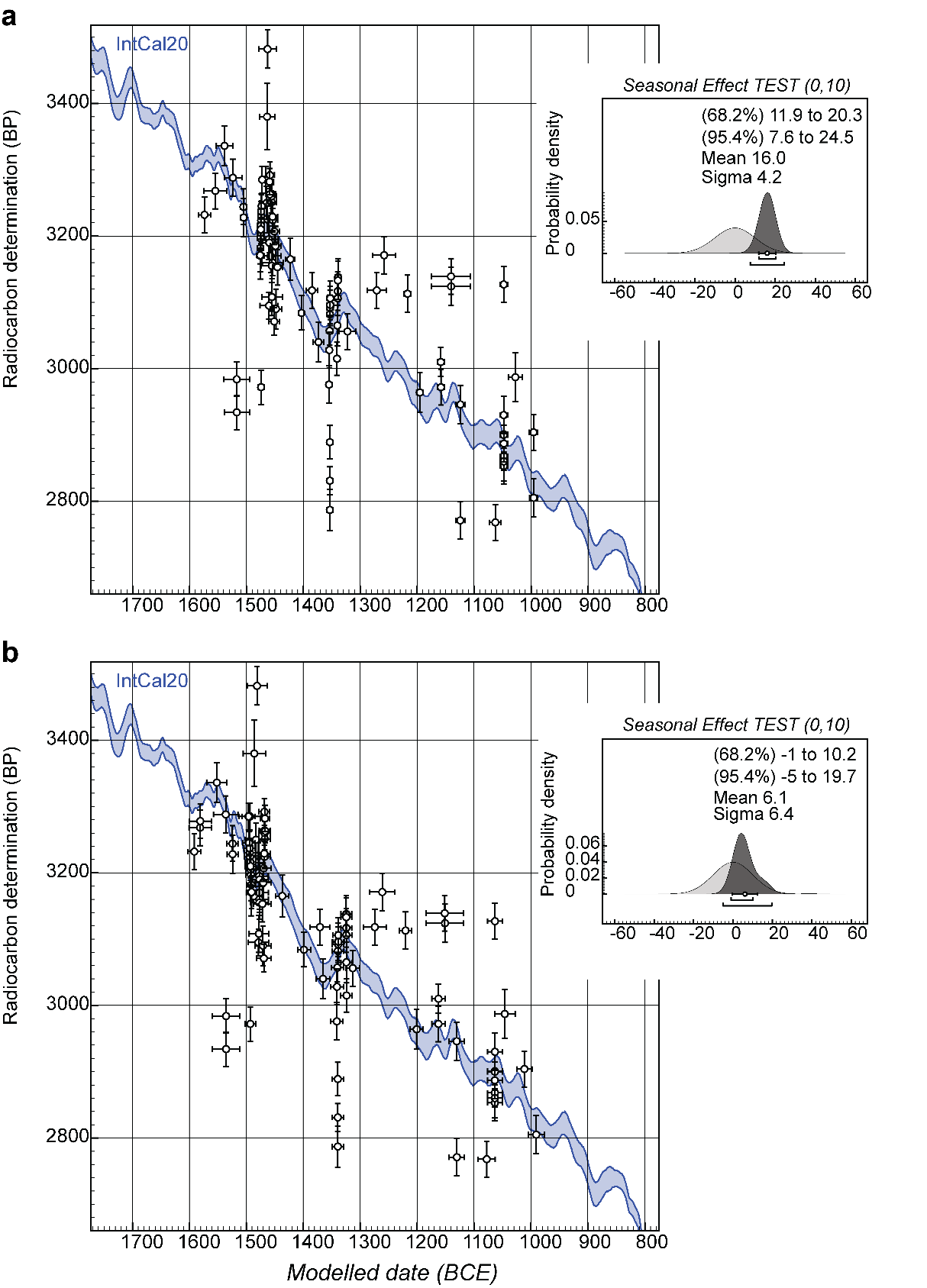
**

**Supplementary Fig. S5.** Comparison of two published Egyptian NK models re-run with IntCal20 [ref. 1], OxCal [ref. 23] 4.1.7, neutral ΔR Seasonal Offset Tests of 0±10 ^14^C years [ref. 24], and curve resolution set at 1 year. **a**, Original model [ref. 7] (Shaw version) re-run with IntCal20 (±1σ). **b**, Alternative Egyptian NK model [ref. 33] using reign lengths adjusted to reflect recent historical chronology discussion [refs. 34–36], and using the longest plausible (‘ultra-high’) reigns options for the 18^th^ Dynasty [refs. 34, S141] (so a ‘maximum’ case). The slightly longer chronology and hence slightly different placements of the ^14^C data within the NK time series result in differing values for a comparison of the Egyptian data versus IntCal20 in terms of an offset. Thus the OxCal ΔR values are 16±4 ^14^C years for **a**, but only 6±6 ^14^C years for **b**. The model in **a** places the start of the NK (accession Ahmose) ~1563-1553 BCE (68.3% hpd), ~1569-1548 BCE (95.4% hpd) (range usually 0-1 year over multiple runs); the model in **b** places the accession of Ahmose ~1586-1577 BCE (68.3% hpd), ~1590-1572 (81-92%), 1568-1556 (4-14%) BCE (95.4% hpd) (range of 0-1 years on 68.3% ranges, 0-1 years on most likely sub-range and 0-5 years on less likely sub-range within the 95.4% ranges over multiple runs). Two data (extreme outliers) do not plot within the view shown. Models with Convergence (C) values ≥95 used.

**

**

**Supplementary Fig. S6.** The wiggle-match of the 186 Arizona (AA) annual ^14^C dates on samples from the Gordion (GOR) chronology [ref. 38] and (Inset) the best fit placement for the first year dated (Gordion RY 834) against IntCal20 [ref. 1] using OxCal [ref. 22]. This fit places the series 1678±1 BCE (3627±1 Cal BP) to 1493±1 BCE (3441±1 Cal BP). Thus the end of the 1028-years Gordion chronology (RY1764) is placed 748±1 BCE (~3 years earlier than the preferred date in ref. [38]). The weighted average offset of the AA GOR time series (μ±σ) versus IntCal20 is 11.2±1.9 ^14^C years. The trend in the AA GOR data conforms well generally with IntCal20. Whether the ‘blips’ to older ^14^C ages ~1595–1590 BCE (3544–3549 Cal BP), ~1554 – 1551 BCE (3503 – 3500 Cal BP) and ~1512–1511 BCE (3461–3460 Cal BP) are real (and to what scale), or rather represent noise/outliers is unclear – all include data more than 2σ from either the 10 point or 20 point weighted adjacent average suggesting probably substantially noise/outliers. The AA Gor data overall are a little more positively offset 1600–1540 BCE (16.2±3.2 ^14^C years versus 11.2±1.9 ^14^C years), or, if we adjust the AA GOR data by the difference between parallel AA IrO and ETH IrO (6.2±1.8 ^14^C years [ref. 39]), and remove those data over 2σ from the 10 point weighted adjacent average, this becomes a positive offset across this period of ~7.8±3.8 ^14^C years. Hence we consider the impact of a maximum possible offset of ~8 ^14^C years in the main text.


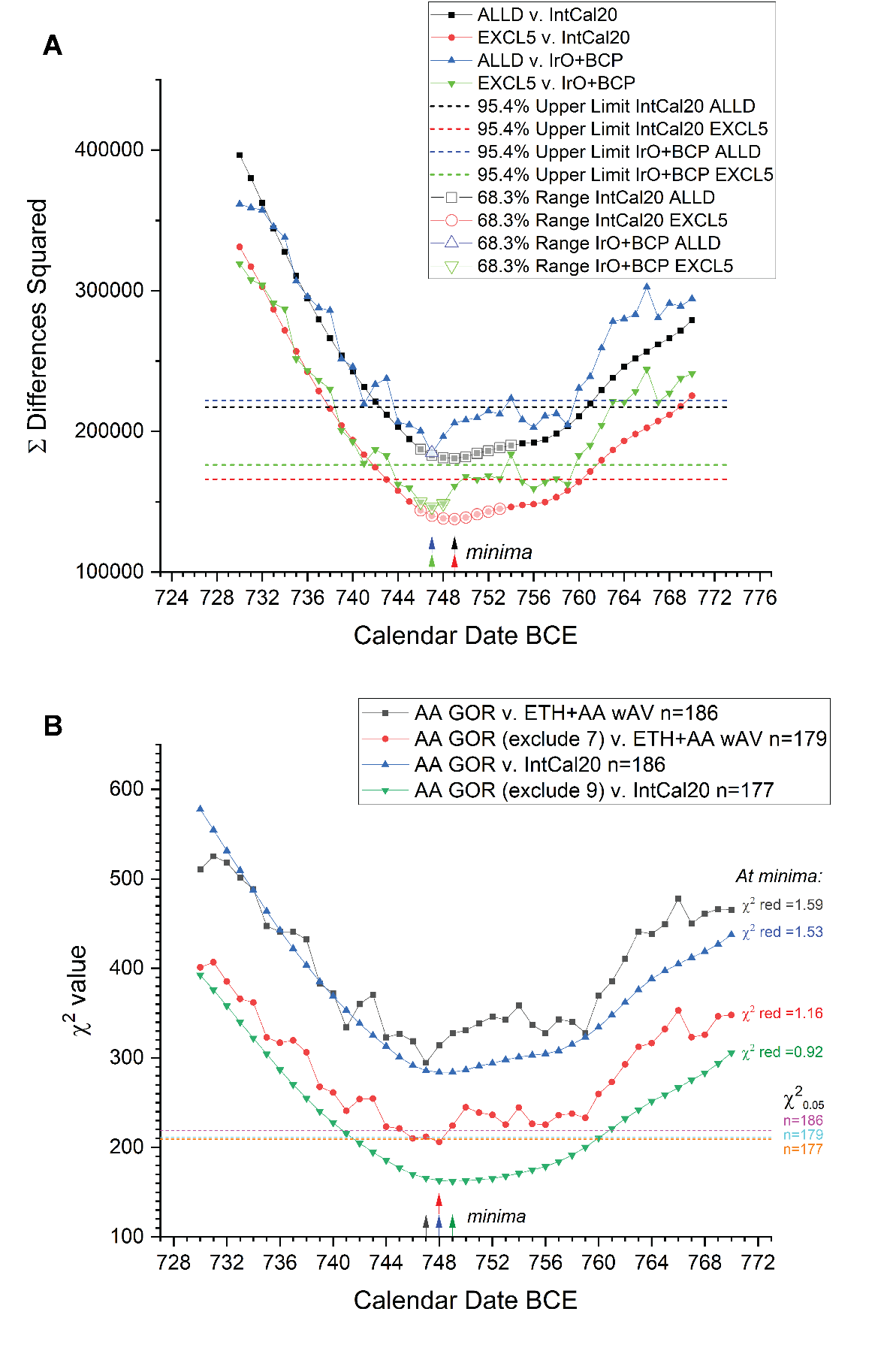


**Supplementary Fig. S7.** A. Least squares curve fitting of the AA Gor dataset [ref. 38] (all data, ALLD, or minus 5 largest outliers, EXCL5 – see Supplementary Discussion 2) versus IntCal20 [ref. 1] and the weighted average of the AA and ETH IrO data in ref. [39]. Curve fitting method follows ref. [40]. The minima and calculated χ^2^ 95.4% and 68.3% fit ranges are indicated. B. χ^2^ test of the AA GOR dataset versus IntCal20 and against the weighted average of the ETH IrO+AA IrO+AA BCP (ETH+AA wAV) following method in ref. [41] in terms of the placement of the last tree-ring and felling date GOR RY1764 (see Supplementary Discussion 2). The all-data (n=186) series do not offer a fit within the χ^2^ 0.05 (95%) critical threshold in either case. The fits excluding those data where the difference between the AA GOR data set and the reference curve is >3 times the measurement error on the AA data (n=179 v. ETH+AA wAV, n=177 v. IntCal20) are also shown. The n=179 series versus ETH+AA wAV offers fits at 749 BCE and 747 BCE within the χ^2^ 0.05 threshold (748 BCE very narrowly misses also), and the n=177 series offers fits versus IntCal20 within the χ^2^ 0.05 threshold from 759–742 BCE.





**Supplementary Fig. S8.** Comparison of the Hd GOR and AA GOR datasets against IntCal20 [ref. 1]. The Hd GOR data are shown as published, and also with a +11.8±2.1 ^14^C years adjustment (comparable to the difference between Hd GeO and ETH IrO between 3625–3431 Cal BP/1676–1482 BCE: see main text). Such an adjustment then produces a large average offset of Hd GOR to IntCal20, +14.2±2.1 ^14^C years adjustment, which appears much too large (see main text). Thus, while the Hd GeO data in this period appear too recent for unknown reasons, we use the Hd GOR data as published since these data offer appropriate values. The AA GOR data, placed at GOR RY1764 = 748 BCE, are noisy with several major outliers, but on average are +11.2±1.9 ^14^C years older than IntCal20. This appears too large an average offset (see main text, Supplementary Discussion 1). If the AAG GOR measurements are adjusted by the difference between AA and ETH data on parallel IrO (+6.2±1.8 ^14^C years, AA older: see Fig. 3e, ref. [39]), then the difference becomes more plausible at ~5.0±3.3 ^14^C years. Inset: this shows the period with the AA GOR data in more detail (see also fig. 3e). While noisy and with outliers, a 10 point FFT smoothing of the AA GOR data follows IntCal20 fairly closely, and if the data were reduced a little, e.g. by the AA IrO to ETH IrO offset, even more so. Both this and a linear interpolation of the Hd GOR data indicate at most a little by way of a positive ^14^C offset in the 16^th^ century BCE (there is a minor upward wiggle in the AA GOR data ~3544-3599 Cal BP/1595–1590 BCE, but the apparently too old ^14^C measurement for AA GOR RY921 placed at 1591 BCE is partly responsible). The ‘blip’ around 3500 Cal BP/1551 BCE is the possible exception, but this unusual occurrence needs replication—otherwise it appears likely to be another instance of substantial noise/outliers in this dataset. See also Supplementary Fig. S6, and especially Supplementary Fig. S14.

**

**

**Supplementary Fig. S9.** Hd GeO, Hd TuP, Hd IrO and Hd BCH (*Cedrus libani*, from Bcharre, Lebanon) ^14^C data versus IntCal20. The OxCal ΔR [ref. 24] offsets for a neutral prior of 0±10 ^14^C years are listed. Data from refs. [10, 37, S197].


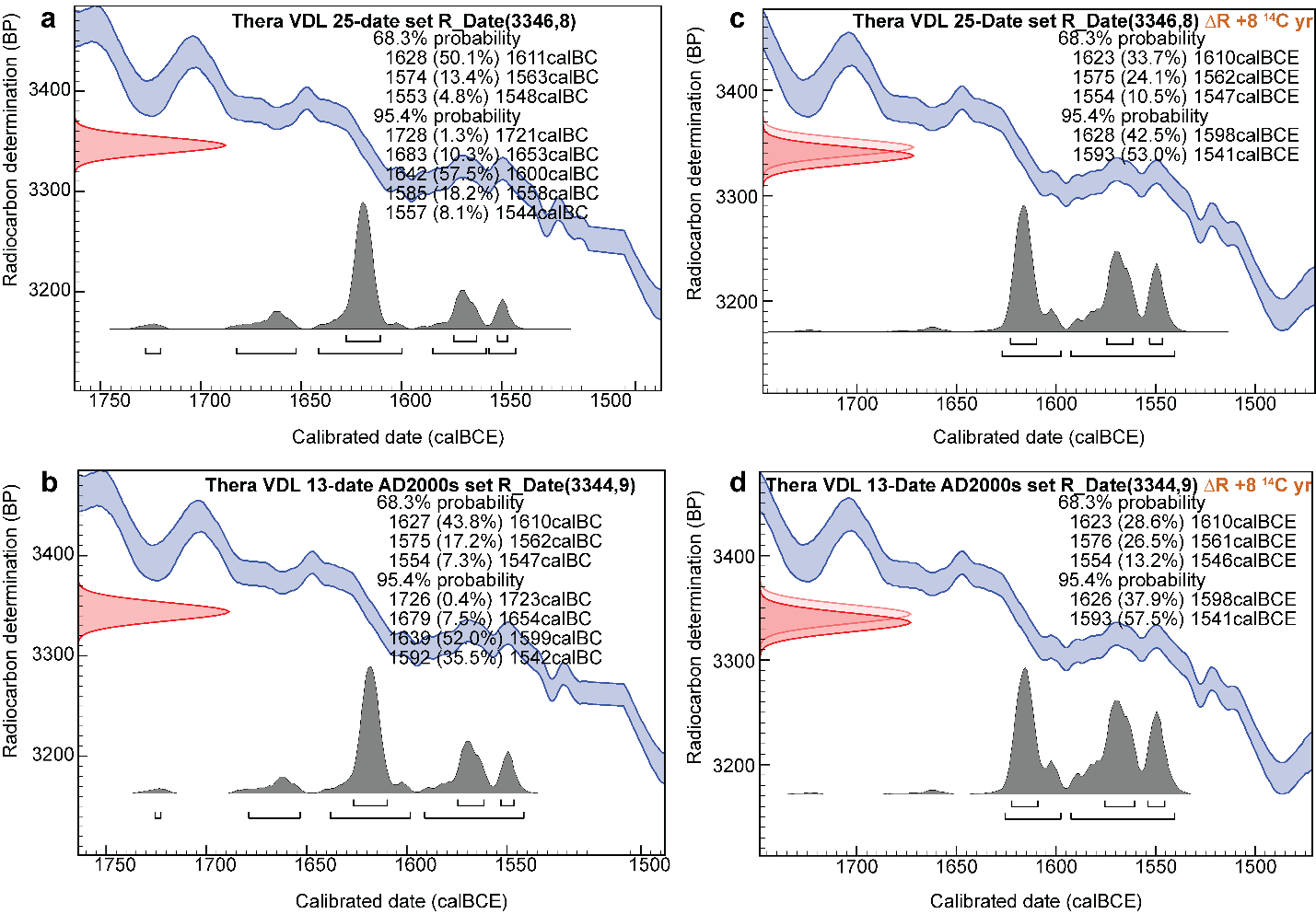


**Supplementary Fig. S10. Calibrated age probabilities for weighted average ^14^C ages for the Thera volcanic destruction level (VDL) with IntCal20. (a)** calibration of the weighted average age from the set of 25 dates on short-lived samples from the Akrotiri VDL from the Oxford (OxA), Copenhagen (K) and Vienna (VERA) laboratories [refs. 33, 52–54] with IntCal20. **(b)** the same but using only the 13 dates (from the OxA and VERA laboratories) run in the AD2000s. **(c)** as **(a)** but adding in a hypothetical +8 year Aegean ^14^C year offset. **(d)** as **(b)** but adding in a hypothetical +8 year Aegean ^14^C year offset. Data from OxCal [ref. 23] 4.4.1 with IntCal20 [ref. 1]. Curve resolution set at 1 year.


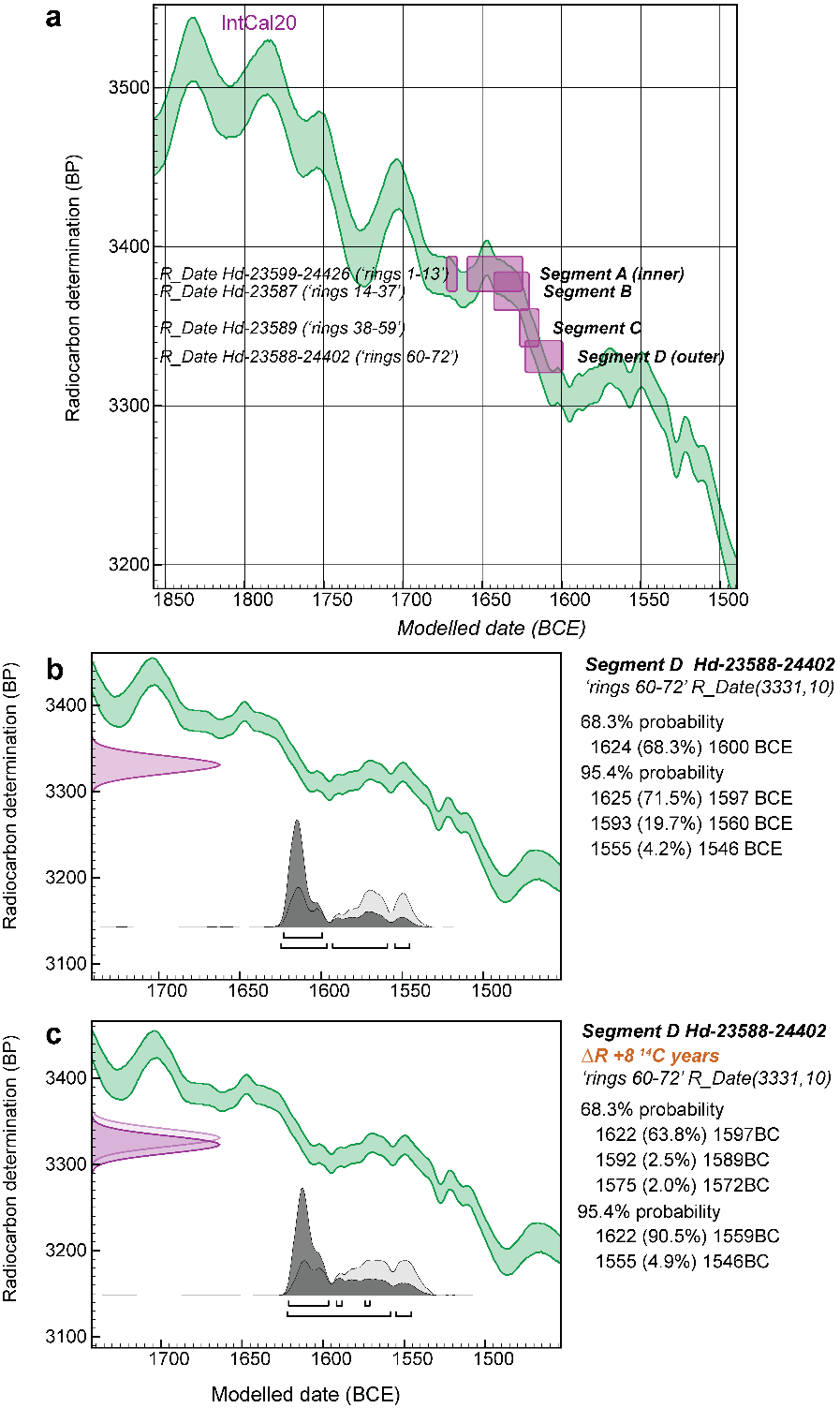


**Supplementary Fig. S11.** Dating the Santorini olive branch ordered sequence of ^14^C dates [refs. 54, 56] from the inner segment (Segment A) to outer segment (Segment D) of the sample against IntCal20 (±1σ) [ref. 1] (no ring count employed [ref. 55]). **(a)** 68.3% hpd ranges for each sample placed against the IntCal20 ^14^C calibration curve. **(b)** Modelled calibrated probability (black) and non-modelled calibrated probability (grey) for the last (outer) segment (D) from **(a)**. **(c)** As **(b)** but with an arbitrary ΔR of 8 ^14^C years included – probability remains strongly in last two decades of the 17^th^ century BCE. Data from OxCal [refs. 22–24] 4.4.1 using the Sequence function and IntCal20, resolution 1 year.


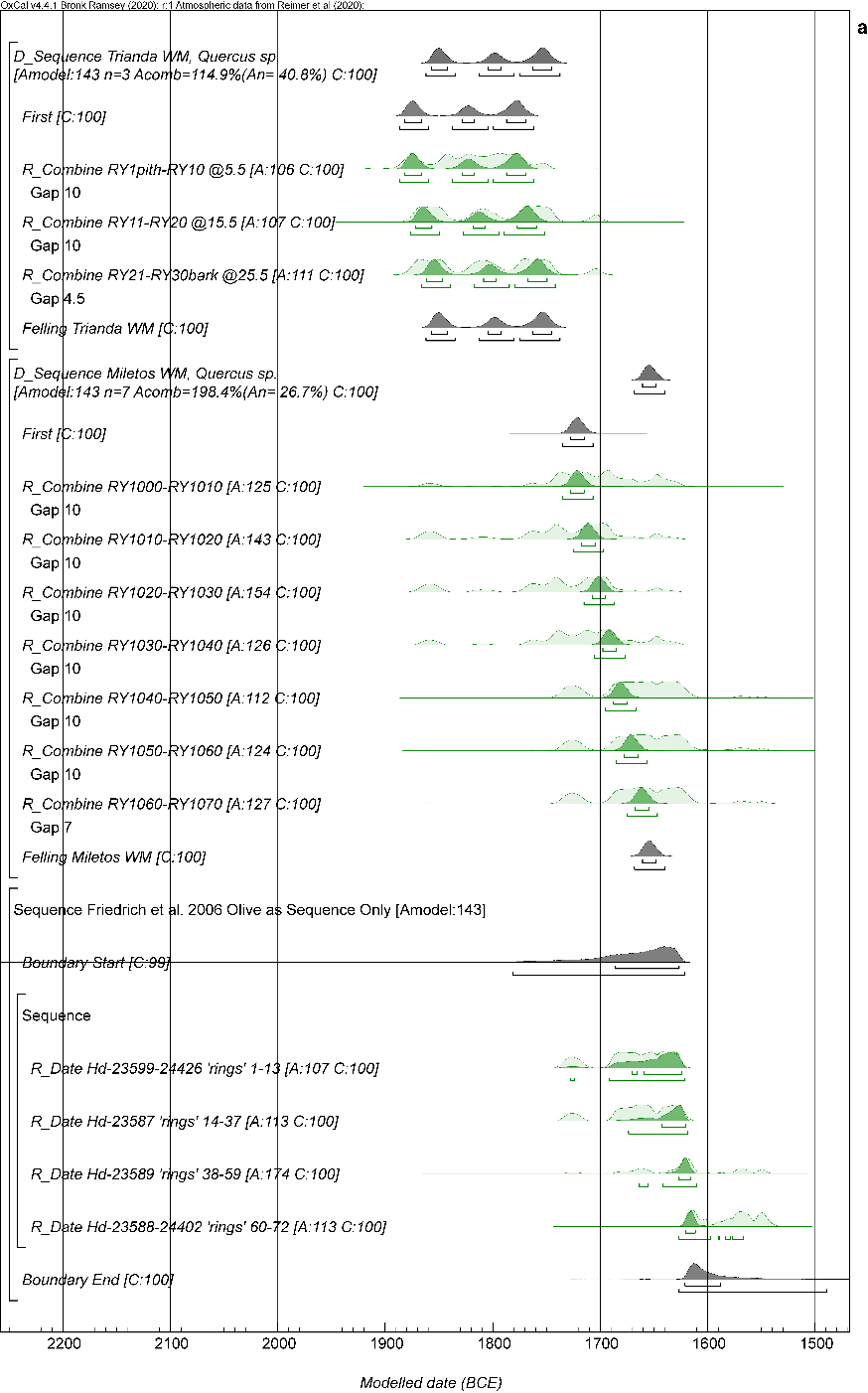


**Supplementary Fig. S12. (a)–(f).** Aegean OxCal 4.4.1 Bayesian chronological model [refs. 22–24] using IntCal20 [ref. 1] incorporating ^14^C data and archaeological sequence information for samples from Thera, Miletos, Rhodes and Crete. Curve resolution set at 1 year. See Supplementary Discussion 3 and Table S3. The Sequence is a modification of previous work [ref. 52], updated and revised to include data from refs. S109, S110, S124, S160, S164–S168. Part **a** (above) shows three preliminary wiggle-match or Sequence analyses which are then cross-referenced within the main Sequence model in (**b), (d)** (below). The red arrows (see parts (**b)** and (**d**)) indicate the 3 samples excluded from the model with an outlier query (probabilities for being in the correct position are ~0-1% only across multiple model runs). Light histograms indicate non-modelled calendar probabilities for ^14^C dates. Dark (solid) histograms indicate modelled probabilities. The lines under the solid histograms indicate the 68.3% hpd and 95.4% hpd ranges. The lines and brackets on the left indicate the model structure. **(f)** shows a summary of key elements from **(a)** to **(e)**.


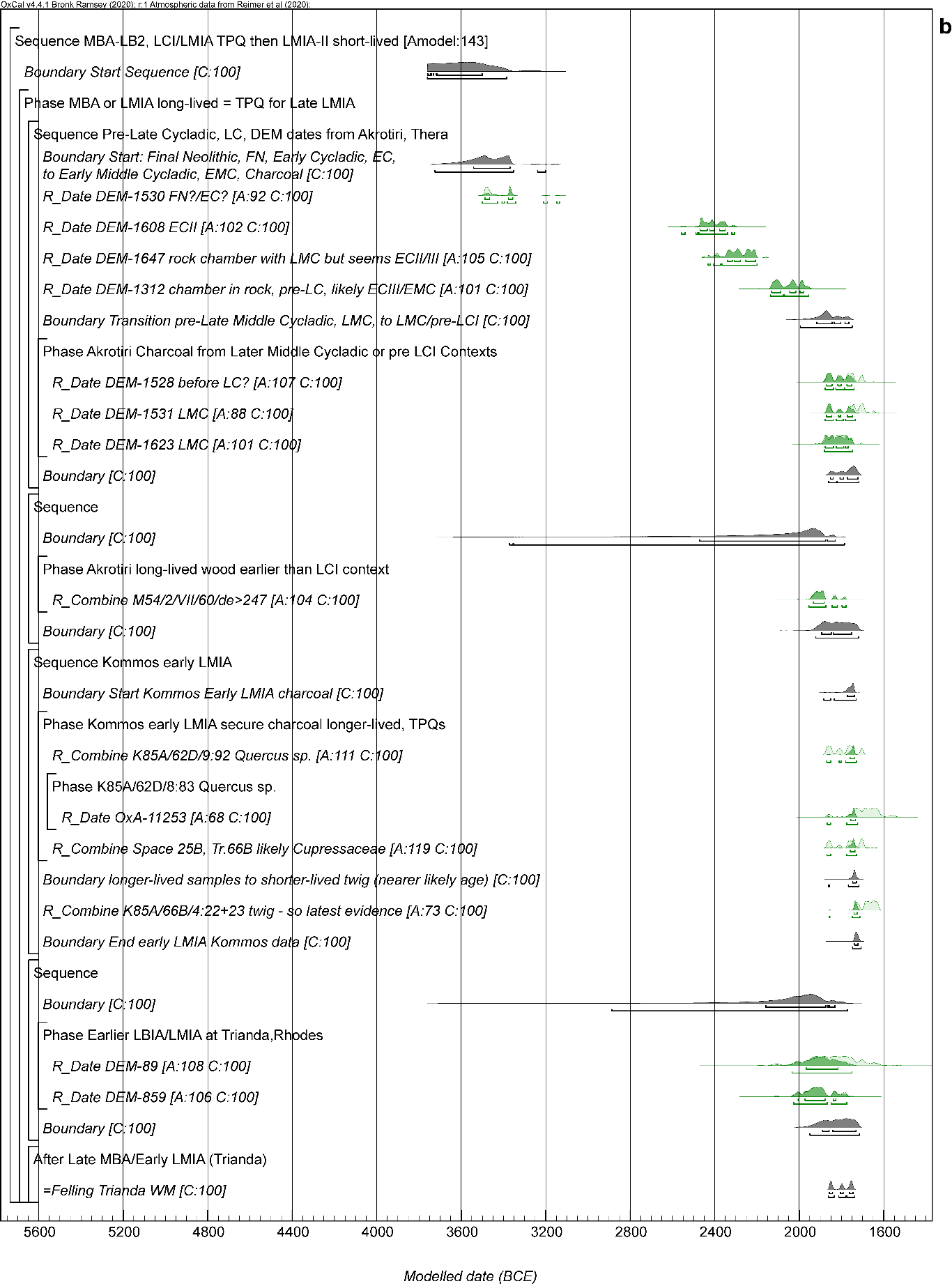


**Supplementary Fig. S12.** Part **b.**


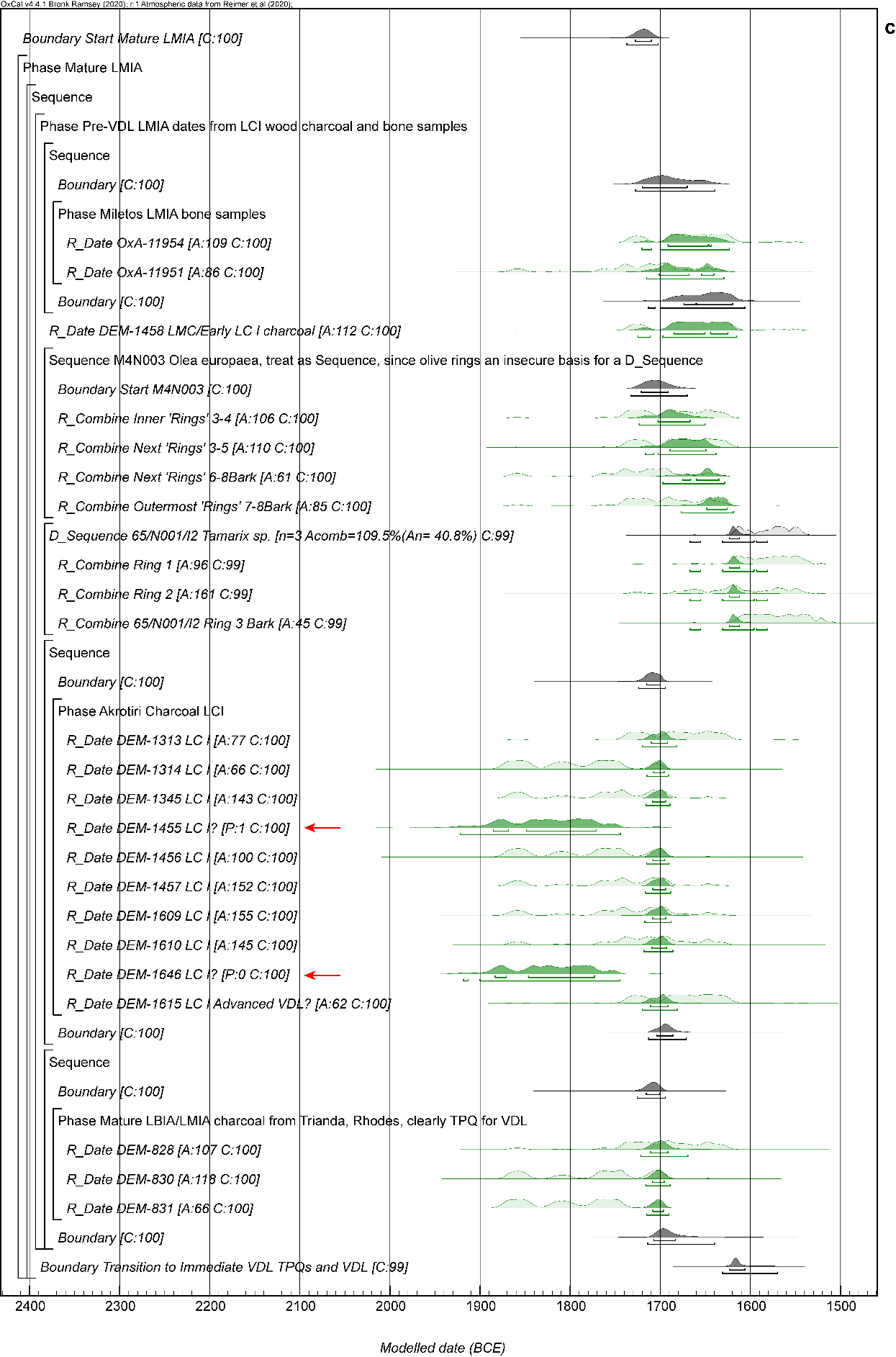


**Supplementary Fig. S12.** Part **c**.


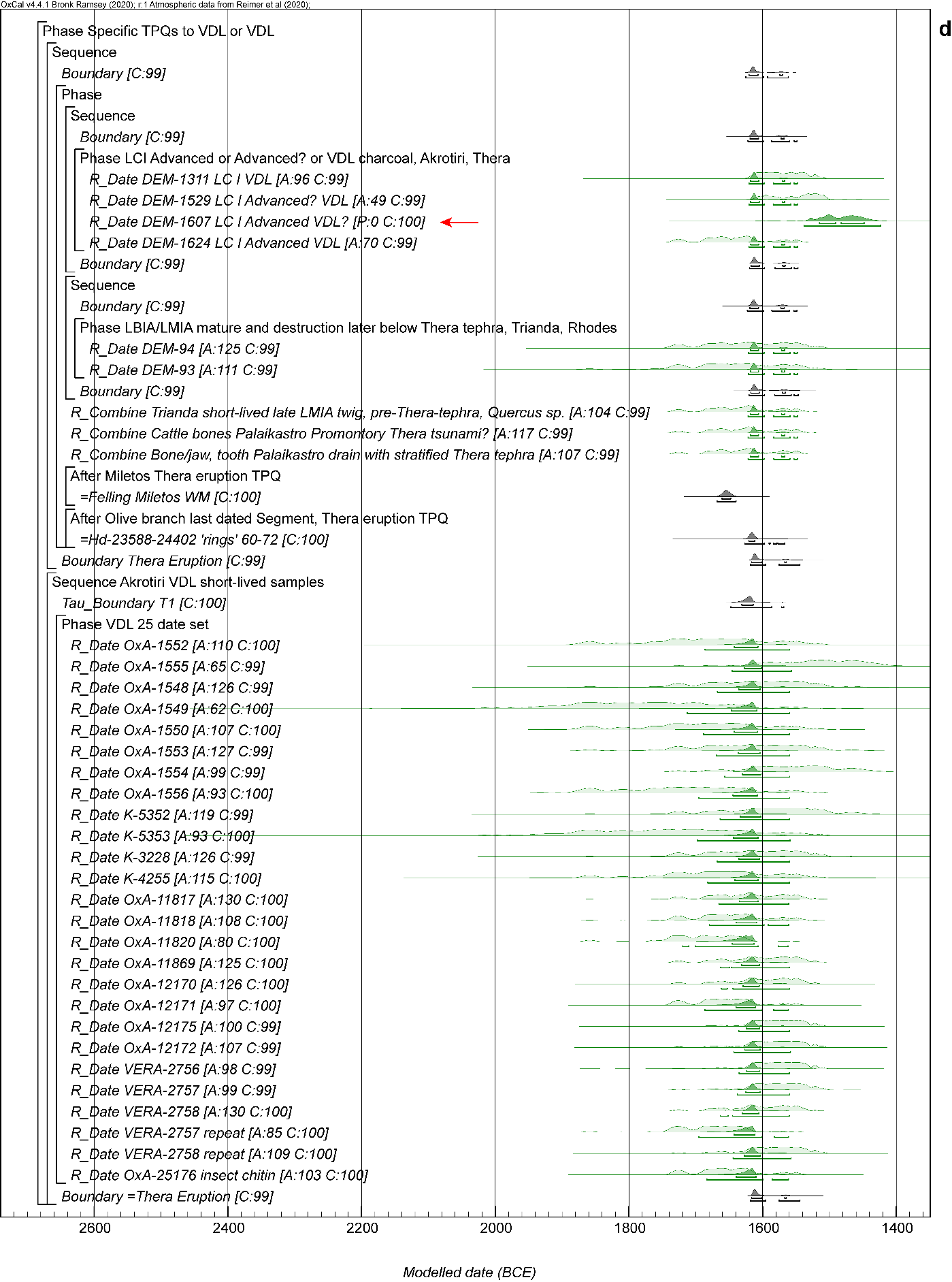


**Supplementary Fig. S12.** Part **d.**


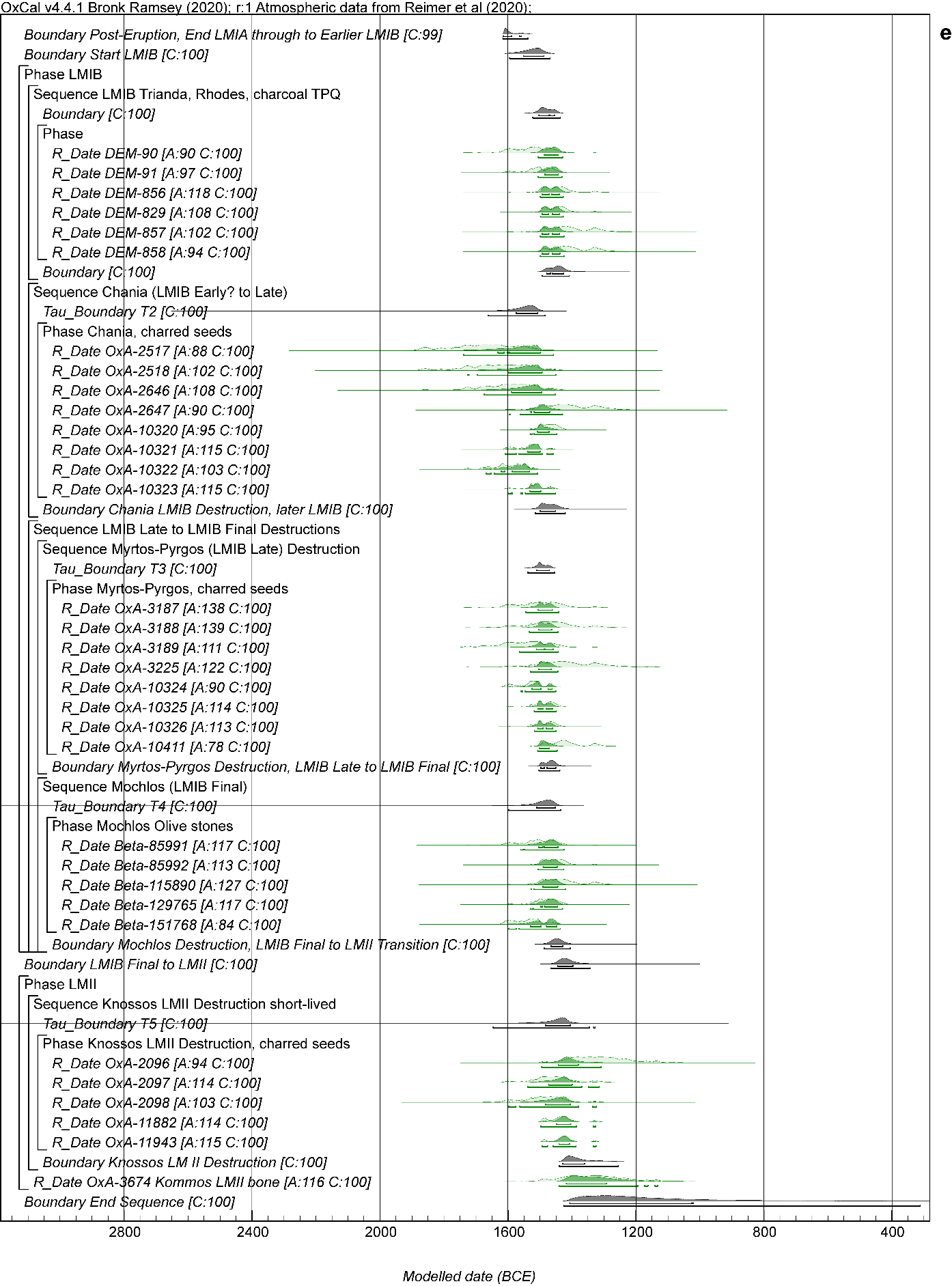


**Supplementary Fig. S12.** Part **e.**

**
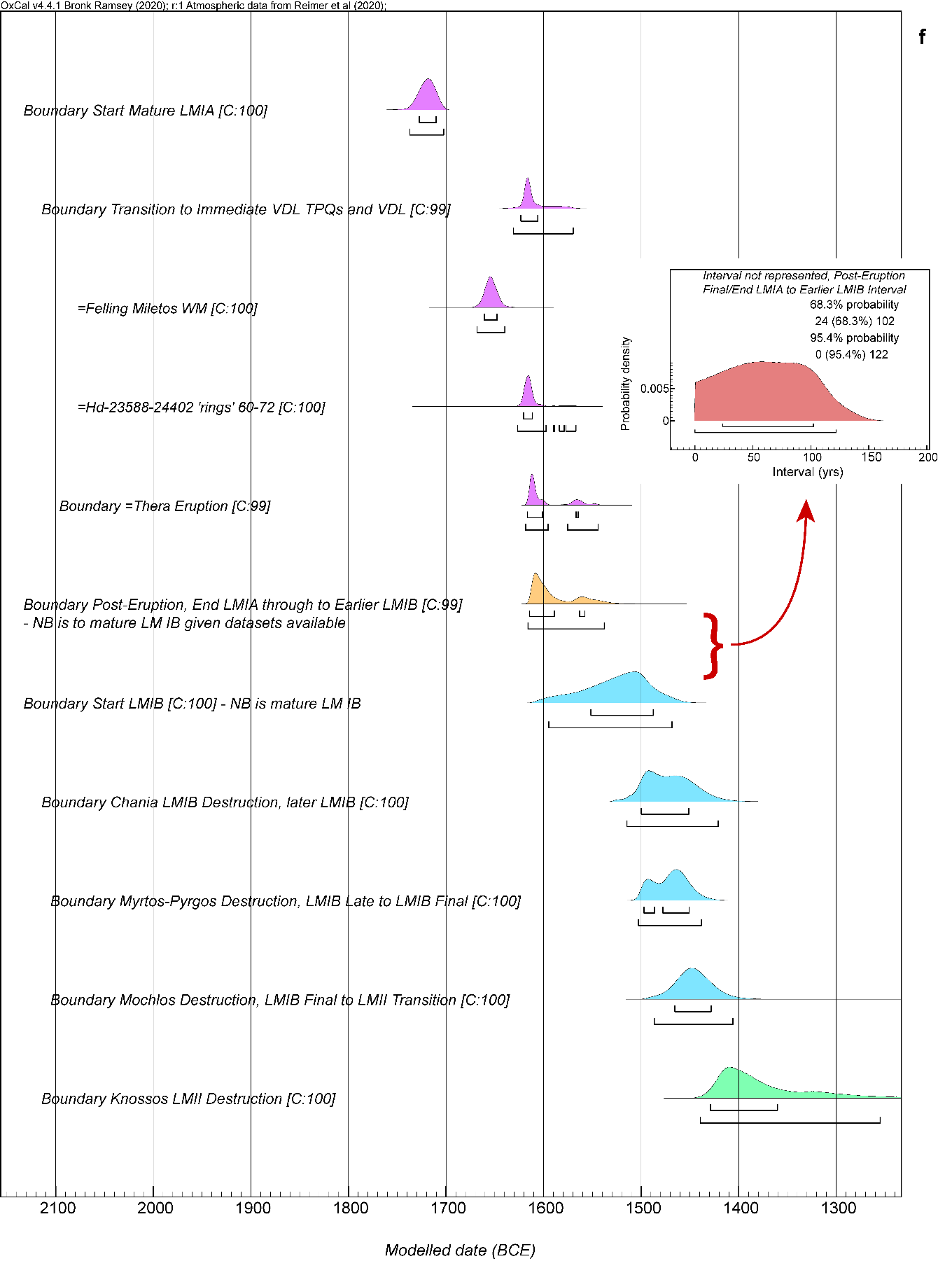
**

**Supplementary Fig. S12.** Part **f.** Selected elements from **(a)** to **(e)** and the Interval query between end LM IA and the beginning of represented (that is mature) LM IB in the model.





**Supplementary Fig. S13. The modelled Thera Eruption Boundary from 11 different runs of the Fig. 6a model.** The model results are all very similar with only very minor variation. The range of the 68.3% hpd and 95.4% hpd ranges are listed along with the average hpd percentage probabilities (and the minimum and maximum values across the 11 model runs). The run with the median A_model_ value is run 11 (which is the model result shown in Fig. 6a).





**Supplementary Fig. S14. The AA GOR data series placed against IntCal20 excluding those n=16 AA GOR data where the difference to IntCal20 is >2 times their measurement errors (>2SD).** The χ^2^ best fit of this ‘cleaned’ series versus IntCal20 has a minimum value for GOR RY1764 at 749 BCE. The χ^2^ reduced value is ~0.76. The weighted average difference of the (remaining n=170) AA GOR data v. IntCal20 is 9.9±2.0 ^14^C years. If the ETH IrO to AA IrO difference (-6.2±1.8 ^14^C years) or the AA IrO to IntCal20 difference (-5.8±2.7 ^14^C years) (see Supplementary Discussion 1) were subtracted, this would indicate a more likely real offset between AA GOR and IntCal20 of just 3.7±2.7 ^14^C years or 4.1±3.4 ^14^C years respectively. This is small to negligible. The apparent increased offset 1600-1540 BCE (see Supplementary Discussion 1) is also much reduced considering this ‘cleaned’ dataset to an additional and small to negligible 1.5±4.0 ^14^C years (and was clearly largely a product of several large outliers in the data).


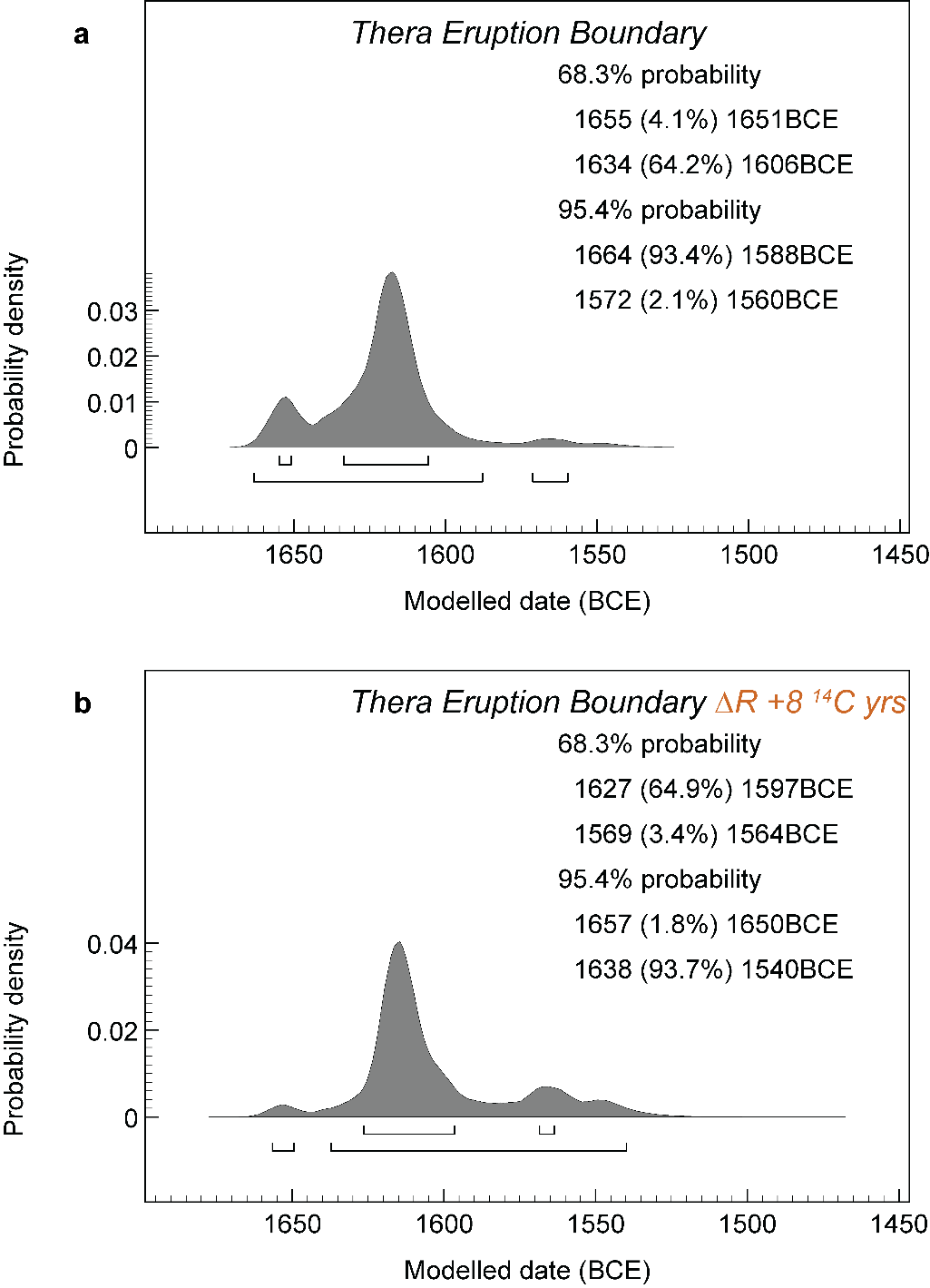


**Supplementary Fig. S15.** The Fig. 6 models and Thera Eruption Boundary date estimates re-run excluding all dates from Thera and hence avoiding any possibility of effects on samples from volcanic ^14^CO_2_ (or any other Thera-specific issue). In the past critics of an earlier date for the Thera eruption have regularly suggested that volcanic ^14^CO_2_ contamination might have affected some or all of the ^14^C dates from Thera [refs. S157–S159], despite no positive evidence for this among any of the dates on archaeologically relevant samples and despite similar ^14^C ages being measured on samples from late LM IA contexts from loci well away from Thera and so removed from any putative volcanic CO_2_ effect [refs. 33, 52, 54, 56]. Hence this test of a no-Thera-data model. **(a)** The Fig. 6a model using IntCal20 unmodified. **(b)** The Fig. 6b model re-run allowing for a hypothetical +8 ^14^C years Aegean offset (see main text and Supplementary Discussions 1, 3). Data from OxCal [refs. 22–24] 4.4.1 and IntCal20 with curve resolution set at 1 year.


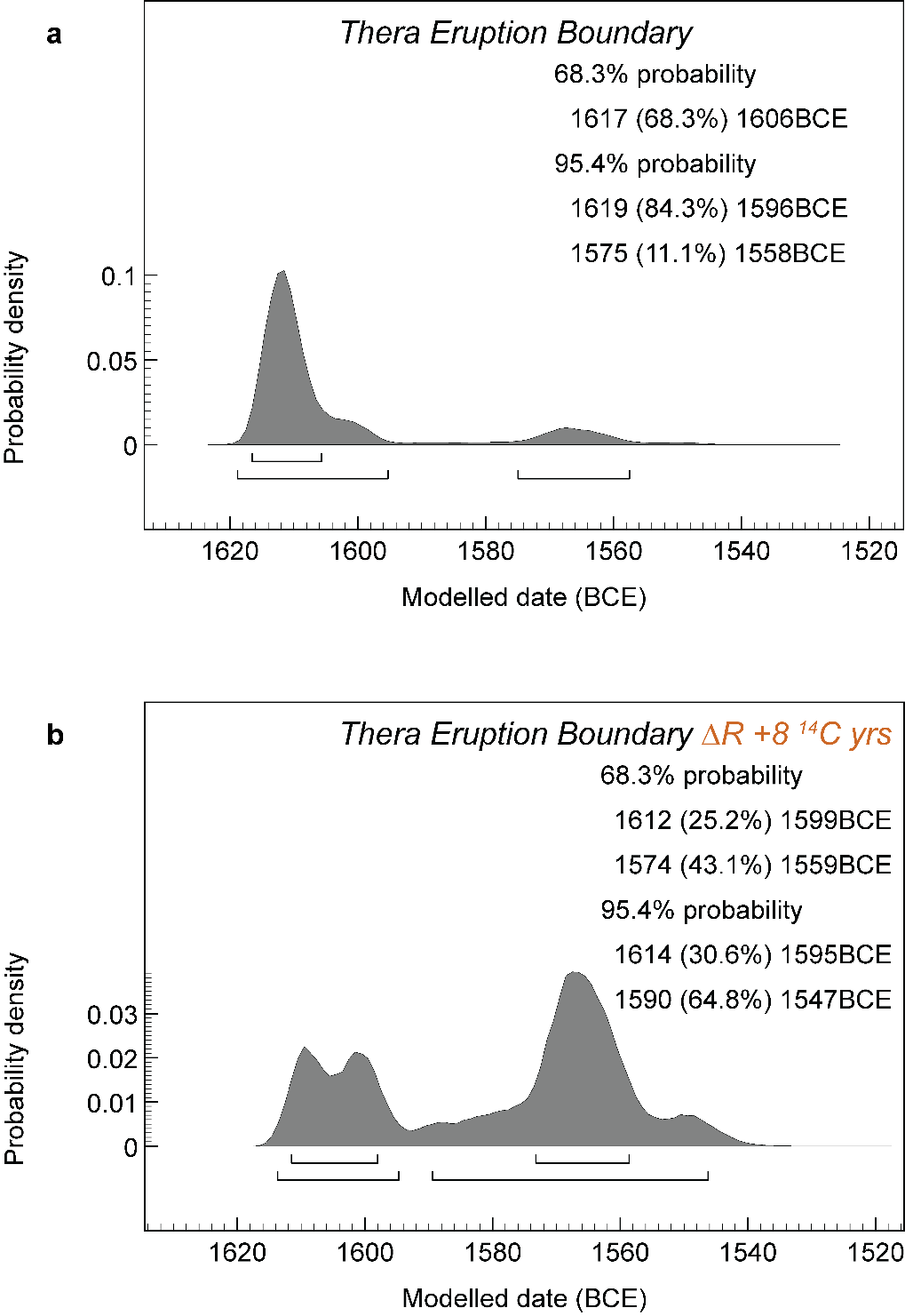


**Supplementary Fig. S16.** The Thera Eruption Boundary from revised versions of the Fig. 6a and 6b models adding in the two ^14^C dates on a small piece of charcoal from above the Thera tephra later in Pelekita Cave [ref. S95] as an eruption *terminus ante quem* (TAQ). **(a)** The Fig. 6a model revised using unmodified IntCal20. **(b)** The Fig. 6b model revised allowing for a hypothetical +8 ^14^C years Aegean offset (see main text and Supplementary Discussions 1, 3). The results are largely similar to the results shown in Fig. 6a and b respectively. The difference the possible TAQ makes is in each to reduce the probability and extent of the mid-16^th^ century BCE range a little. Thus in **(a)** the 95.4% hpd range ends 1558 BCE versus 1545 BCE in Fig. 6a, and in **(b)** the 95.4% ranges ends 1547 BCE versus 1538 BCE. Data from OxCal [refs. 22–24] 4.4.1 and IntCal20 with curve resolution set at 1 year.





**Supplementary Fig. S17.** The distribution of the differences in ^14^C ages between pairs of same age AA GOR and AA IrO samples [refs. 38, 39] according to placement of the AA GOR dataset (either with GOR RY1764 = 745 BCE as in refs. [38, 39], or with GOR RY1764 = 748 BCE as in this paper).

**Supplementary Table S1.** ETH ^14^C results on tree-rings from Middle Bronze Age (MBA) Acemhöyük (ACM), Turkey. The δ^13^C values shown are those from the AMS.

| **ETH ID** | **Sample ID** | **Sample RY** | **Species** | **^14^C Age BP** | **SD** | **δ^13^C  (‰)** |
| --- | --- | --- | --- | --- | --- | --- |
| 78941.1.1 | ACM-48A-1A | 637-645 | *Juniperus* sp. | 3515 | 16 | -25.4 |
| 78941.1.2 | ACM-48A-1A | 637-645 | *Juniperus* sp. | 3520 | 15 | -20.8 |
| 78942.1.1 | ACM-48A-2A | 647-655 | *Juniperus* sp. | 3576 | 15 | -18.5 |
| 78943.1.1 | ACM-48A-3A | 656-662 | *Juniperus* sp. | 3532 | 15 | -21.4 |
| 78944.1.1 | ACM-48A-4A | 657-665 | *Juniperus* sp. | 3527 | 15 | -22.6 |
| 78945.1.1 | ACM-48A-5A | 667-675 | *Juniperus* sp. | 3541 | 15 | -23.1 |
| 78945.2.1 | ACM-48A-5A | 667-675 | *Juniperus* sp. | 3545 | 15 | -20.3 |
| 78946.1.1 | ACM-48A-6A | 677-685 | *Juniperus* sp. | 3546 | 15 | -23.2 |
| 78946.2.1 | ACM-48A-6A | 677-685 | *Juniperus* sp. | 3540 | 15 | -23.2 |
| 78947.1.1 | ACM-48A-7A | 687-695 | *Juniperus* sp. | 3556 | 15 | -20.1 |
| 78949.1.1 | ACM-48A-9A | 640-642 | *Juniperus* sp. | 3524 | 15 | -22.4 |
| 78950.1.2 | ACM-48A-10A | 645-647 | *Juniperus* sp. | 3546 | 15 | -22.1 |
| 78951.1.1 | ACM-48A-11A | 650-652 | *Juniperus* sp. | 3517 | 16 | -26.3 |
| 78951.1.2 | ACM-48A-11A | 650-652 | *Juniperus* sp. | 3541 | 15 | -23.2 |
| 78952.1.1 | ACM-48A-12A | 655-657 | *Juniperus* sp. | 3513 | 15 | -20.1 |
| 78953.1.1 | ACM-48A-13A | 660-662 | *Juniperus* sp. | 3521 | 15 | -23.0 |
| 78954.1.1 | ACM-48A-14A | 665-667 | *Juniperus* sp. | 3547 | 15 | -21.0 |
| 78955.1.1 | ACM-48A-15A | 670-672 | *Juniperus* sp. | 3551 | 15 | -22.9 |
| 78956.1.1 | ACM-48A-16A | 675-677 | *Juniperus* sp. | 3550 | 15 | -22.0 |
| 78957.1.1 | ACM-48A-17A | 680-682 | *Juniperus* sp. | 3516 | 15 | -23.5 |
| 78958.1.1 | ACM-48A-18A | 685-687 | *Juniperus* sp. | 3526 | 16 | -23.2 |
| 78959.1.1 | ACM-48A-19A | 690-692 | *Juniperus* sp. | 3505 | 15 | -19.7 |
| 78960.1.1 | ACM-48A-20A | 695-697 | *Juniperus* sp. | 3487 | 15 | -20.2 |
| 78961.1.1 | ACM-48A-21A | 700-702 | *Juniperus* sp. | 3488 | 15 | -23.7 |
| 78961.1.2 | ACM-48A-21A | 700-702 | *Juniperus* sp. | 3505 | 15 | -24.9 |

**Supplementary Table S2.** ETH ^14^C results on known-age single-year oak tree-rings from Erstein, Switzerland. The known-age tree-rings come from ERST 5964-218-37 [refs. 25, 26]. The δ^13^C values shown are those from the AMS.

| **ETH ID** | **Sample ID** | **Sample Date BCE** | **Species** | **^14^C Age BP** | **SD** | **δ^13^C (‰)** |
| --- | --- | --- | --- | --- | --- | --- |
| 76306.1.1 | ERST006-218-37 | 1834 | *Quercus* sp. | 3527 | 21 | -29.8 |
| 76315.1.1 | ERST006-218-37 | 1843 | *Quercus* sp. | 3498 | 21 | -31.4 |
| 76317.1.1 | ERST006-218-37 | 1845 | *Quercus* sp. | 3523 | 21 | -30.1 |
| 76319.1.1 | ERST006-218-37 | 1847 | *Quercus* sp. | 3519 | 21 | -32.7 |
| 76321.1.1 | ERST006-218-37 | 1849 | *Quercus* sp. | 3502 | 21 | -29.6 |
| 76323.1.1 | ERST006-218-37 | 1851 | *Quercus* sp. | 3525 | 21 | -30.3 |
| 76325.1.1 | ERST006-218-37 | 1853 | *Quercus* sp. | 3522 | 21 | -32.3 |
| 76327.1.1 | ERST006-218-37 | 1855 | *Quercus* sp. | 3525 | 21 | -31.1 |
| 76331.1.1 | ERST006-218-37 | 1859 | *Quercus* sp. | 3496 | 21 | -28.2 |

**Supplementary Table S3.** Parts 1–9. OxCal CQL2 code for the models used for the wiggle-match analyses and analyses shown in Figs. 1–6 and explanatory information. We annotate these to indicate the outliers mentioned in the text. The user must select IntCal20 (default calibration curve with the release of IntCal20 in OxCal 4.4) or, if using earlier versions, and having uploaded the IntCal20 data file to the user’s OxCal directory, call it as a file, e.g.:

Curve("IntCal20","IntCal20.14c");

*Contents of Supplementary Table S3:*

1. MBA time series wiggle-match (for Fig. 1, Supplementary Figs. S1–S3).
2. Hd GOR time series wiggle-match (for Fig. 3a, c, Supplementary Fig. S8).
3. Miletos and Noceto ^14^C dating model for Fig. 4a using published data [refs. 10, 52].
4. Amarna period ^14^C dating model for Fig. 4b using data previously published [refs. 7, 51].
5. Model for dating the Thera/Santorini volcanic eruption by ^14^C using an appropriate method previously published [refs. 53, 54].
6. Model for Thera olive branch as a Sequence in OxCal (Fig. 5).
7. Aegean ^14^C model for the periods before, around, and after the Thera eruptions, modifying and extending the Bayesian chronological model previously published [ref. 52], adding information, approaches and data from refs. [53, 54, 56, S109, S110, S124, S160, S164–S168]: see Fig. 6a, Supplementary Figs. S12, S13, Table S4. The Fig. 6b model added a Delta_R of 8 ^14^C years.
8. The Aegean model in 7 but excluding all the data from Thera (Supplementary Fig. S15).
9. The Aegean model in 7 but adding in the Pelekita Cave TAQ (Supplementary Fig. S16).

**1. MBA time series wiggle-match (for Fig. 1, Supplementary Figs. S1–S3).** This file is the Fig. 1a version. The three outliers and one excluded sample/date from the Supplementary Figs. S1–S3 versions are noted. For Fig. 1b, the Delta_R line is changed from 0,10 to 22,5.

Options()

{

Resolution=1;

//IntCal20 default calibration file – otherwise insert call here, e.g.: Curve="IntCal20.14c";

};

Plot()

{

Delta_R("Seasonal Offset",0,10);

//See Methods and Supplementary Fig. S2

Outlier_Model("SSimple",N(0,2),0,"s");

D_Sequence ("MBA, KUL + KBK + ACM")

{

R_Date ("OxA-29954 KUL RY 497-505 @ 501",3647,27)

{

Outlier ("SSimple",0.05);

color="blue";

};

Gap(10);

R_Date ("OxA-30900 KUL RY 507-515 @ 511", 3657,31)

{

Outlier ("SSimple",0.05);

color="blue";

};

Gap(3.5);

R_Date ("Hd-22955 KBK RY 509-520 @ 514.5",3684,11)

{

Outlier ("SSimple",0.05);

color="brown";

};

Gap (6.5);

R_Date ("OxA-29955 KUL RY 517-525 @ 521",3618,28)

{

Outlier ("SSimple",0.05);

color="blue";

};

Gap(4);

R_Date ("Hd-22956 KBK RY 520-530 @ 525",3660,11)

{

Outlier ("SSimple",0.05);

color="brown";

};

Gap (6);

R_Date ("OxA-30901 KUL RY 527-535 @ 531", 3641,32)

{

Outlier ("SSimple",0.05);

color="blue";

};

Gap(4);

R_Date ("Hd-22957 KBK RY 530-540 @ 535",3627,17)

{

Outlier ("SSimple",0.05);

color="brown";

};

Gap (6);

R_Combine ("RY 537-545 @ 541 KUL")

{

Outlier("SSimple",0.05);

color="blue";

R_Date ("OxA-29956 KUL @541",3570,28)

{

Outlier ("SSimple",0.05);

};

R_Date ("OxA-29957 KUL @541", 3613,28)

{

Outlier ("SSimple",0.05);

};

};

Gap(3);

R_Date ("OxA-30902 KUL RY 543-545 @ 544", 3675,30)

{

Outlier ("SSimple",0.05);

color="blue";

};

Gap(1);

R_Date ("Hd-22986 KBK RY 540-550 @ 545",3630,15)

{

Outlier ("SSimple",0.05);

color="brown";

};

Gap (6);

R_Date ("OxA-30903 KUL RY 547-555 @ 551", 3596,31)

{

Outlier ("SSimple",0.05);

color="blue";

};

Gap(4);

R_Date ("Hd-23043 KBK RY 550-560 @ 555",3618,15)

{

Outlier ("SSimple",0.05);

color="brown";

};

Gap (6);

R_Date ("OxA-29958 KUL RY 557-565 @ 561", 3678,29)

{

Outlier ("SSimple",0.05);

color="blue";

};

Gap(4);

R_Date ("Hd-22987 KBK RY 560-570 @ 565",3633,14)

{

Outlier ("SSimple",0.05);

color="brown";

};

Gap (6);

R_Combine ("RY 567-575 @ 571 KUL")

{

Outlier("SSimple",0.05);

color="blue";

R_Date ("OxA-30904 KUL RY 567-575 @ 571", 3607,30)

{

Outlier ("SSimple",0.05);

};

R_Date ("OxA-30905 KUL RY 567-575 @ 571", 3575,33)

{

Outlier ("SSimple",0.05);

};

};

Gap(4);

R_Date ("Hd-23045 KBK RY 570-580 @ 575",3604,17)

{

Outlier ("SSimple",0.05);

color="brown";

};

Gap (6);

R_Date ("OxA-29959 KUL RY 577-585 @ 581", 3584,29)

{

Outlier ("SSimple",0.05);

color="blue";

};

Gap(4);

R_Date ("Hd-23026 KBK RY 580-590 @ 585",3570,14)

{

Outlier ("SSimple",0.05);

color="brown";

};

Gap (6);

R_Date ("OxA-30906 KUL RY 587-595 @ 591", 3572,31)

{

Outlier ("SSimple",0.05);

color="blue";

};

Gap(4);

R_Date ("Hd-23027 KBK RY 590-600 @ 595",3563,17)

{

Outlier ("SSimple",0.05);

color="brown";

};

Gap (6);

R_Date ("OxA-29960 KUL RY 597-605 @ 601", 3582,28)

{

Outlier ("SSimple",0.05);

color="blue";

};

Gap(4);

R_Date ("Hd-22958 KBK RY 600-610 @ 605",3578,20)

{

Outlier ("SSimple",0.05);

color="brown";

};

Gap (7);

//R_Date ("OxA-30907 KUL RY 607-615 @ 611", 3669, 29)

//{

// Outlier ("SSimple",0.05);

// color="blue";

//};

//NB high offset δ13C AMS v MS - grounds to be suspicious - EXCLUDE

//Gap(1);

R_Combine ("RY 609-615 @ 612 ACM")

{

Outlier("SSimple",0.05);

color="Black";

R_Date ("OxA-30890 ACM RY 609-615 @ 612", 3566,32)

{

Outlier ("SSimple",0.05);

};

R_Date ("OxA-31514 ACM RY 609-615 @ 612", 3564,32)

{

Outlier ("SSimple",0.05);

};

};

Gap(9);

R_Combine ("KUL & ACM RY 617-625 @ 621")

{

Outlier("SSimple",0.05);

color="purple";

R_Date ("OxA-29961 KUL RY 617-625 @ 621", 3539,27)

{

Outlier ("SSimple",0.05);

};

R_Date ("OxA-30892 ACM RY 617-625 @ 621", 3582,32)

{

Outlier ("SSimple",0.05);

};

R_Date ("OxA-31515 ACM RY 617-625 @ 621", 3613,31)

{

Outlier ("SSimple",0.05);

};

};

Gap(10);

R_Combine ("KUL & ACM RY 627-635 @ 631")

{

Outlier("SSimple",0.05);

color="purple";

R_Date ("OxA-30908 KUL RY 627-635 @ 631", 3626,31)

{

Outlier ("SSimple",0.05);

};

R_Date ("OxA-30892 ACM RY 627-635 @ 631", 3532,33)

{

Outlier ("SSimple",0.05);

};

R_Date ("OxA-31516 ACM RY 627-635 @ 631", 3535,31)

{

Outlier ("SSimple",0.05);

};

};

Gap(10);

R_Combine ("KUL & ACM RY @ 641")

{

Outlier("SSimple",0.05);

color="purple";

R_Date ("OxA-29962 KUL RY 637-645 @ 641", 3522,28)

{

Outlier ("SSimple",0.05);

};

R_Date("ETH78949.1.1 ACM RY640-642 @ 641",3524,15)

{

Outlier ("SSimple",0.05);

};

R_Date("ETH78941.1.1 ACM RY637-645 @ 641",3515,16)

{

Outlier ("SSimple",0.05);

};

R_Date("ETH78941.1.2 ACM RY637-645 @ 641",3520,15)

{

Outlier ("SSimple",0.05);

};

R_Date ("OxA-30893 ACM RY 637-645 @ 641", 3559,32)

{

Outlier ("SSimple",0.05);

};

R_Date ("OxA-31517 ACM RY 637-645 @ 641", 3473,32)

{

Outlier ("SSimple",0.05);

};

};

Gap(5);

R_Date("ETH78950.1.2 ACM 645-647 @ 646",3546,15)

{

Outlier ("SSimple",0.05);

color="black";

};

Gap(5);

R_Combine ("KUL & ACM RY @ 651")

{

Outlier("SSimple",0.05);

color="purple";

R_Date ("OxA-30909 KUL RY 647-655 @ 651", 3490,30)

{

Outlier ("SSimple",0.05);

};

//R_Date("ETH78942.1.1 ACM RY 647-655 @ 651",3576,15)

//{

// Outlier ("SSimple",0.05);

//};

//Outlier Initial Model ~53%

R_Date ("OxA-30894 ACM RY 647-655 @ 651", 3548,32)

{

Outlier ("SSimple",0.05);

};

R_Date ("OxA-31518 ACM RY 647-655 @ 651", 3495,31)

{

Outlier ("SSimple",0.05);

};

R_Date("ETH78951.1.1 ACM RY 650-652 @ 651",3517,16)

{

Outlier ("SSimple",0.05);

};

R_Date("ETH78951.1.2 ACM RY 650-652 @ 651",3541,15)

{

Outlier ("SSimple",0.05);

};

};

Gap(5);

R_Date("ETH78952.1.1 ACM 655-657 @ 656",3513,15)

{

Outlier ("SSimple",0.05);

color="black";

};

Gap(3);

//R_Combine ("KUL & ACM RY @ 659")

//{

//Outlier("SSimple",0.05);

//color="purple";

//R_Date ("OxA-29963 KUL RY 656-662 @ 659", 3457,28)

//{

// Outlier ("SSimple",0.05);

//};

//Outlier Initial Model ~65%

R_Date("ETH78943.1.1 ACM RY 656-662 @ 659",3532,15)

{

Outlier ("SSimple",0.05);

color=”black”;

};

//};

Gap(2);

R_Combine ("RY @ 661 ACM")

{

Outlier("SSimple",0.05);

color="black";

R_Date("ETH78944.1.1 ACM RY 657-665 @ 661",3527,15)

{

Outlier ("SSimple",0.05);

};

R_Date ("OxA-30895 ACM RY 657-665 @ 661", 3588,32)

{

Outlier ("SSimple",0.05);

};

R_Date ("OxA-31519 ACM RY 657-665 @ 661", 3540,31)

{

Outlier ("SSimple",0.05);

};

R_Date("ETH78953.1.1 ACM RY 660-662 @ 661",3521,15)

{

Outlier ("SSimple",0.05);

};

};

Gap(5);

R_Date("ETH78954.1.1 ACM RY 665-667 @ 666",3547,15)

{

Outlier ("SSimple",0.05);

color="black";

};

Gap(5);

R_Combine ("RY @ 671 ACM")

{

Outlier("SSimple",0.05);

color="black";

R_Date("ETH78945.1.1 ACM RY 667-675 @ 671",3541,15)

{

Outlier ("SSimple",0.05);

};

R_Date("ETH78945.2.1 ACM RY 667-675 @ 671",3545,15)

{

Outlier ("SSimple",0.05);

};

R_Date ("OxA-30896 ACM RY 667-675 @ 671", 3547,33)

{

Outlier ("SSimple",0.05);

};

R_Date ("OxA-31520 ACM RY 667-675 @ 671", 3528,31)

{

Outlier ("SSimple",0.05);

};

R_Date("ETH78955.1.1 ACM RY 670-672 @ 671",3551,15)

{

Outlier ("SSimple",0.05);

};

};

Gap(2);

Date("RY673");

Gap(3);

R_Date("ETH78956.1.1 ACM RY 675-677 @ 676",3550,15)

{

Outlier ("SSimple",0.05);

color="black";

};

Gap(5);

R_Combine("RY @ 681 ACM")

{

Outlier("SSimple",0.05);

color="black";

R_Date("ETH78946.1.1 ACM RY 677-685 @ 681",3546,15)

{

Outlier ("SSimple",0.05);

};

R_Date("ETH78946.2.1 ACM RY 677-685 @ 681",3540,15)

{

Outlier ("SSimple",0.05);

};

R_Date("ETH78957.1.1 ACM RY 680-682 @ 681",3516,15)

{

Outlier ("SSimple",0.05);

};

R_Date ("OxA-30897 ACM RY 677-685 @ 681", 3593,32)

{

Outlier ("SSimple",0.05);

};

R_Date ("OxA-31521 ACM RY 677-685 @ 681", 3492,31)

{

Outlier ("SSimple",0.05);

};

};

Gap(5);

R_Date("ETH78958.1.1 ACM RY 685-687 @ 686",3526,16)

{

Outlier ("SSimple",0.05);

color="black";

};

Gap(5);

R_Combine ("RY @ 691 ACM")

{

Outlier("SSimple",0.05);

color="black";

//R_Date("ETH78947.1.1 ACM RY 687-695 @ 691",3556,15)

//{

// Outlier ("SSimple",0.05);

//};

//Outlier Initial Model ~84%

R_Date ("OxA-30898 ACM RY 687-695 @ 691", 3499,32)

{

Outlier ("SSimple",0.05);

};

R_Date ("OxA-31522 ACM RY 687-695 @ 691", 3452,39)

{

Outlier ("SSimple",0.05);

};

R_Date("ETH78959.1.1 ACM RY 690-692 @ 691",3505,15)

{

Outlier ("SSimple",0.05);

};

};

Gap(5);

R_Date("ETH78960.1.1 ACM RY 695-697 @ 696",3487,15)

{

Outlier ("SSimple",0.05);

color="black";

};

Gap(5);

R_Combine ("RY @ 701 ACM")

{

Outlier("SSimple",0.05);

color="black";

R_Date ("OxA-30899 ACM RY 697-705 @ 701", 3498,32)

{

Outlier ("SSimple",0.05);

};

R_Date ("OxA-31523 ACM RY 697-705 @ 701", 3470,31)

{

Outlier ("SSimple",0.05);

};

R_Date("ETH78961.1.1 ACM RY 700-702 @ 701",3488,15)

{

Outlier ("SSimple",0.05);

};

R_Date("ETH78961.1.2 ACM RY 700-702 @ 701",3505,15)

{

Outlier ("SSimple",0.05);

};

};

Gap(31);

Date("RY732");

};

};

**2. Hd GOR time series wiggle-match (for Fig. 3a, c, Supplementary Fig. S8).** This file is listed with all data but excludes the first 14 larger individual outliers to achieve satisfactory OxCal agreement indices as used for the Fig. 3 fit version. The dates excluded are marked in orange. Note: where there is an R_Combine, in particular for RY906.5, we remove the individual date indicated as the outlier (RY906.5b) and re-run, rather than removing both elements of the R_Combine. Removing 13 outliers is required (in order from largest downwards) to achieve almost satisfactory OxCal A_model_ and A_overall_ values around 60 (58 and 61). Removal of the 14 individual outliers (as indicated below) achieves Amodel and Aoverall values of around 72 and 74. Note: to run the file with the data excluded (e.g. all data which achieves a placement ~750 BCE for RY1764) the Gap(); statements around the dates currently excluded must be appropriately adjusted. The numbers, e.g. “776.5”, are the GOR chronology Relative Years (RY). The dates are listed by their RY numbers below. For the Hd lab codes, see previous publications [refs. 5, 10, 37].

Options()

{

Resolution=1;

//IntCal20 default calibration file – otherwise insert call here, e.g.: Curve="IntCal20.14c";

};

Plot()

{

Outlier_Model("SSimple",N(0,2),0,"s");

D_Sequence("HdGOR")

{

Date("RY737");

Gap(39.5);

R_Date("776.5", 3430, 14)

{

Outlier ("SSimple",0.05);

};

Gap(10);

R_Date("786.5", 3409, 13)

{

Outlier ("SSimple",0.05);

};

Gap(10);

R_Date("796.5", 3432, 16)

{

Outlier ("SSimple",0.05);

};

Gap(10);

R_Date("806.5", 3426, 13)

{

Outlier ("SSimple",0.05);

};

Gap(10);

R_Date("816.5", 3403, 18)

{

Outlier ("SSimple",0.05);

};

Gap(20);

//R_Date("826.5", 3335, 16)

//{

// Outlier ("SSimple",0.05);

//};

//Gap(10);

//Outlier ~28/29%

R_Date("836.5", 3348, 17)

{

Outlier ("SSimple",0.05);

};

Gap(10);

R_Date("846.5", 3401, 16)

{

Outlier ("SSimple",0.05);

};

Gap(10);

R_Date("856.5", 3372, 13)

{

Outlier ("SSimple",0.05);

};

Gap(10);

R_Date("866.5", 3356, 18)

{

Outlier ("SSimple",0.05);

};

Gap(10);

R_Date("876.5", 3342, 16)

{

Outlier ("SSimple",0.05);

};

Gap(10);

R_Date("886.5", 3334, 18)

{

Outlier ("SSimple",0.05);

};

Gap(10);

R_Date("896.5", 3336, 19)

{

Outlier ("SSimple",0.05);

};

Gap(10);

R_Combine ("906.5")

{

Outlier("SSimple",0.05);

R_Date("906.5a", 3280, 16)

{

Outlier ("SSimple",0.05);

};

//R_Date("906.5b", 3235, 17)

//{

// Outlier ("SSimple",0.05);

//};

//Outlier ~55-56%

};

Gap(10);

R_Combine ("916.5")

{

Outlier("SSimple",0.05);

R_Date("916.5a", 3329, 14)

{

Outlier ("SSimple",0.05);

};

R_Date("916.5b", 3302, 14)

{

Outlier ("SSimple",0.05);

};

};

Gap(9);

R_Date("925.5", 3323, 22)

{

Outlier ("SSimple",0.05);

};

Gap(1);

R_Combine ("926.5")

{

Outlier("SSimple",0.05);

R_Date("926.5a", 3308, 16)

{

Outlier ("SSimple",0.05);

};

R_Date("926.5b", 3284, 17)

{

Outlier ("SSimple",0.05);

};

};

Gap(9);

R_Date("935.5", 3329, 23)

{

Outlier ("SSimple",0.05);

};

Gap(1);

R_Combine ("936.5")

{

Outlier("SSimple",0.05);

R_Date("936.5a", 3338, 18)

{

Outlier ("SSimple",0.05);

};

R_Date("936.5b", 3296, 18)

{

Outlier ("SSimple",0.05);

};

};

Gap(9);

R_Date("945.5", 3330, 21)

{

Outlier ("SSimple",0.05);

};

Gap(1);

R_Combine ("946.5")

{

Outlier("SSimple",0.05);

R_Date("946.5a", 3289, 18)

{

Outlier ("SSimple",0.05);

};

R_Date("946.5b", 3288, 22)

{

Outlier ("SSimple",0.05);

};

};

Gap(10);

R_Combine ("956.5")

{

Outlier("SSimple",0.05);

R_Date("956.5a", 3322, 19)

{

Outlier ("SSimple",0.05);

};

R_Date("956.5b", 3310, 18)

{

Outlier ("SSimple",0.05);

};

};

Gap(10);

R_Combine ("966.5")

{

Outlier("SSimple",0.05);

R_Date("966.5a", 3319, 18)

{

Outlier ("SSimple",0.05);

};

R_Date("966.5b", 3292, 16)

{

Outlier ("SSimple",0.05);

};

};

Gap(10);

R_Date("976.5", 3292, 23)

{

Outlier ("SSimple",0.05);

};

Gap(29);

//R_Date("986.5", 3223, 19)

//{

// Outlier ("SSimple",0.05);

//};

//Outlier ~12%

//Gap(19);

R_Date("1005.5", 3257, 18)

{

Outlier ("SSimple",0.05);

};

Gap(10);

R_Date("1015.5", 3218, 18)

{

Outlier ("SSimple",0.05);

};

Gap(10);

R_Date("1025.5", 3203, 20)

{

Outlier ("SSimple",0.05);

};

Gap(10);

R_Date("1035.5", 3210, 21)

{

Outlier ("SSimple",0.05);

};

Gap(10);

R_Date("1045.5", 3202, 17)

{

Outlier ("SSimple",0.05);

};

Gap(9);

R_Date("1054.5", 3160, 22)

{

Outlier ("SSimple",0.05);

};

Gap(10);

R_Date("1064.5", 3195, 21)

{

Outlier ("SSimple",0.05);

};

Gap(10);

R_Date("1074.5", 3157, 32)

{

Outlier ("SSimple",0.05);

};

Gap(10);

R_Date("1084.5", 3124, 21)

{

Outlier ("SSimple",0.05);

};

Gap(10);

R_Date("1094.5", 3134, 19)

{

Outlier ("SSimple",0.05);

};

Gap(20);

//R_Date("1104.5", 3050, 20)

//{

// Outlier ("SSimple",0.05);

//};

//Gap(10);

//Outlier ~18%

R_Date("1114.5", 3072, 17)

{

Outlier ("SSimple",0.05);

};

Gap(10);

R_Date("1124.5", 3046, 18)

{

Outlier ("SSimple",0.05);

};

Gap(10);

R_Date("1134.5", 3074, 18)

{

Outlier ("SSimple",0.05);

};

Gap(0.5);

R_Combine ("1135")

{

Outlier("SSimple",0.05);

R_Date("1135a", 3106, 18)

{

Outlier ("SSimple",0.05);

};

R_Date("1135b", 3062, 24)

{

Outlier ("SSimple",0.05);

};

};

Gap(9.5);

R_Date("1144.5", 3049, 16)

{

Outlier ("SSimple",0.05);

};

Gap(1);

R_Date("1145.5", 3045, 21)

{

Outlier ("SSimple",0.05);

};

Gap(10);

//R_Date("1155", 3140, 38)

//{

// Outlier ("SSimple",0.05);

//};

//Gap(0.5);

//Outlier ~14-15%

R_Date("1155.5", 3046, 21)

{

Outlier ("SSimple",0.05);

};

Gap(19.5);

R_Date("1175", 3122, 18)

{

Outlier ("SSimple",0.05);

};

Gap(10);

R_Date("1185", 3144, 18)

{

Outlier ("SSimple",0.05);

};

Gap(9.5);

R_Date("1194.5", 3088, 13)

{

Outlier ("SSimple",0.05);

};

Gap(0.5);

R_Date("1195", 3060, 19)

{

Outlier ("SSimple",0.05);

};

Gap(9.5);

R_Date("1204.5", 3055, 13)

{

Outlier ("SSimple",0.05);

};

Gap(10);

R_Date("1214.5", 3090, 18)

{

Outlier ("SSimple",0.05);

};

Gap(0.5);

R_Date("1215", 3058, 25)

{

Outlier ("SSimple",0.05);

};

Gap(49.5);

//R_Date("1224.5", 2996, 17)

//{

// Outlier ("SSimple",0.05);

//};

//Outlier ~14%

//Gap(40);

R_Date("1264.5", 3031, 20)

{

Outlier ("SSimple",0.05);

};

Gap(10);

R_Date("1274.5", 2992, 18)

{

Outlier ("SSimple",0.05);

};

Gap(10);

R_Date("1284.5", 3037, 23)

{

Outlier ("SSimple",0.05);

};

Gap(20);

//R_Date("1294.5", 3051, 21)

//{

// Outlier ("SSimple",0.05);

//};

//Gap(10);

//Outlier ~20-21%

R_Date("1304.5", 2962, 20)

{

Outlier ("SSimple",0.05);

};

Gap(10);

R_Date("1314.5", 2970, 18)

{

Outlier ("SSimple",0.05);

};

Gap(10);

R_Date("1324.5", 2920, 18)

{

Outlier ("SSimple",0.05);

};

Gap(0.5);

R_Date("1325", 2909, 28)

{

Outlier ("SSimple",0.05);

};

Gap(9.5);

R_Date("1334.5", 2956, 23)

{

Outlier ("SSimple",0.05);

};

Gap(0.5);

R_Date("1335", 2929, 20)

{

Outlier ("SSimple",0.05);

};

Gap(9.5);

R_Date("1344.5", 2937, 19)

{

Outlier ("SSimple",0.05);

};

Gap(0.5);

R_Date("1345", 2962, 20)

{

Outlier ("SSimple",0.05);

};

Gap(9.5);

R_Date("1354.5", 2970, 19)

{

Outlier ("SSimple",0.05);

};

Gap(10);

R_Date("1364.5", 2936, 20)

{

Outlier ("SSimple",0.05);

};

Gap(10);

R_Date("1374.5", 2955, 14)

{

Outlier ("SSimple",0.05);

};

Gap(0.5);

R_Date("1375", 2955, 20)

{

Outlier ("SSimple",0.05);

};

Gap(10);

R_Date("1385", 2946, 19)

{

Outlier ("SSimple",0.05);

};

Gap(9.5);

R_Date("1394.5", 2948, 15)

{

Outlier ("SSimple",0.05);

};

Gap(0.5);

R_Date("1395", 2938, 23)

{

Outlier ("SSimple",0.05);

};

Gap(9.5);

R_Date("1404.5", 2907, 17)

{

Outlier ("SSimple",0.05);

};

Gap(0.5);

R_Date("1405", 2899, 26)

{

Outlier ("SSimple",0.05);

};

Gap(9.5);

R_Date("1414.5", 2912, 17)

{

Outlier ("SSimple",0.05);

};

Gap(0.5);

R_Date("1415", 2918, 23)

{

Outlier ("SSimple",0.05);

};

Gap(9.5);

R_Date("1424.5", 2929, 15)

{

Outlier ("SSimple",0.05);

};

Gap(10);

R_Date("1434.5", 2935, 17)

{

Outlier ("SSimple",0.05);

};

Gap(10);

R_Date("1444.5", 2871, 19)

{

Outlier ("SSimple",0.05);

};

Gap(0.5);

R_Date("1445", 2942, 21)

{

Outlier ("SSimple",0.05);

};

Gap(9.5);

R_Date("1454.5", 2935, 16)

{

Outlier ("SSimple",0.05);

};

Gap(10);

R_Date("1464.5", 2896, 15)

{

Outlier ("SSimple",0.05);

};

Gap(20);

//R_Date("1474.5", 2934, 14)

//{

// Outlier ("SSimple",0.05);

//};

//Gap(10);

//Outlier ~33-34%

R_Date("1484.5", 2880, 15)

{

Outlier ("SSimple",0.05);

};

Gap(0.5);

R_Date("1485", 2868, 20)

{

Outlier ("SSimple",0.05);

};

Gap(10);

R_Date("1495", 2899, 14)

{

Outlier ("SSimple",0.05);

};

Gap(10);

R_Date("1505", 2848, 13)

{

Outlier ("SSimple",0.05);

};

Gap(20);

//R_Date("1515", 2895, 20)

//{

// Outlier ("SSimple",0.05);

//};

//Outlier ~24-25%

//Gap(10);

R_Date("1525", 2800, 15)

{

Outlier ("SSimple",0.05);

};

Gap(10);

R_Date("1535", 2816, 20)

{

Outlier ("SSimple",0.05);

};

Gap(10);

R_Date("1545", 2779, 22)

{

Outlier ("SSimple",0.05);

};

Gap(10);

R_Date("1555", 2811, 20)

{

Outlier ("SSimple",0.05);

};

Gap(30);

R_Date("1585", 2786, 20)

{

Outlier ("SSimple",0.05);

};

Gap(20);

R_Date("1605", 2728, 26)

{

Outlier ("SSimple",0.05);

};

Gap(19.5);

R_Date("1624.5", 2746, 25)

{

Outlier ("SSimple",0.05);

};

Gap(10);

//R_Date("1625", 2777, 20)

//{

// Outlier ("SSimple",0.05);

//};

//Gap(9.5);

//Outlier ~14%

R_Date("1634.5", 2748, 18)

{

Outlier ("SSimple",0.05);

};

Gap(0.5);

R_Date("1635", 2709, 13)

{

Outlier ("SSimple",0.05);

};

Gap(9.5);

R_Date("1644.5", 2743, 18)

{

Outlier ("SSimple",0.05);

};

Gap(0.5);

R_Date("1645", 2734, 20)

{

Outlier ("SSimple",0.05);

};

Gap(10);

R_Date("1655", 2746, 20)

{

Outlier ("SSimple",0.05);

};

Gap(10);

R_Date("1665", 2760, 25)

{

Outlier ("SSimple",0.05);

};

Gap(9.5);

R_Date("1674.5", 2730, 16)

{

Outlier ("SSimple",0.05);

};

Gap(0.5);

R_Date("1675", 2720, 20)

{

Outlier ("SSimple",0.05);

};

Gap(9.5);

R_Date("1684.5", 2683, 23)

{

Outlier ("SSimple",0.05);

};

Gap(10);

//R_Date("1685", 2744, 18)

//{

// Outlier ("SSimple",0.05);

//};

//Gap(9.5);

// **Outlier ~11% 14^th^ date to be removed to get Amodel ~72/Aoverall ~74**

R_Date("1694.5", 2712, 18)

{

Outlier ("SSimple",0.05);

};

Gap(0.5);

R_Date("1695", 2662, 13)

{

Outlier ("SSimple",0.05);

};

Gap(10);

R_Date("1705", 2640, 16)

{

Outlier ("SSimple",0.05);

};

Gap(10);

//R_Date("1714.5", 2666, 18)

//{

// Outlier ("SSimple",0.05);

//};

//Gap(0.5);

//Outlier ~18%

R_Date("1715", 2616, 12)

{

Outlier ("SSimple",0.05);

};

Gap(9.5);

R_Date("1724.5", 2589, 21)

{

Outlier ("SSimple",0.05);

};

Gap(0.5);

R_Combine ("1725")

{

Outlier("SSimple",0.05);

R_Date("1725a", 2589, 17)

{

Outlier ("SSimple",0.05);

};

R_Date("1725b", 2549, 19)

{

Outlier ("SSimple",0.05);

};

};

Gap(10);

//R_Date("1734.5", 2610, 17)

//{

// Outlier ("SSimple",0.05);

//};

//Outlier ~12%

//Gap(0.5);

R_Combine ("1735")

{

Outlier("SSimple",0.05);

//R_Date("1735a", 2608, 19)

//{

// Outlier ("SSimple",0.05);

//};

//**Outlier ~19-20%. The 1735 R_Combine fails a χ^2^ test (6.5>3.8), but with just two dates, it is difficult to assign responsibility, and so, since not a major fail, we leave both data in the initial all-data model and the outlier is then shown to be the RY1735a result.**

R_Date("1735b", 2543, 17)

{

Outlier ("SSimple",0.05);

};

};

Gap(9.5);

R_Date("1744.5", 2549, 21)

{

Outlier ("SSimple",0.05);

};

Gap(0.5);

R_Combine ("1745")

{

Outlier("SSimple",0.05);

R_Date("1745a", 2555, 17)

{

Outlier ("SSimple",0.05);

};

R_Date("1745b", 2502, 36)

{

Outlier ("SSimple",0.05);

};

};

Gap(9.5);

R_Date("1754.5", 2530, 25)

{

Outlier ("SSimple",0.05);

};

Gap(9.5);

Date("Date RY1764");

};

};

**3. Miletos and Noceto ^14^C dating model for Fig. 4a using published data [refs. 10, 52].**

Options()

{

Resolution=1;

//IntCal20 default calibration file – otherwise insert call here, e.g.: Curve="IntCal20.14c";

};

//RY Numbers reflect separate Miletos sample and Noceto time series chronologies

Plot()

{

Outlier_Model("SSimple",N(0,2),0,"s");

Delta_R("Test", 0, 10);

D_Sequence( "Miletos WM")

{

R_Combine( "RY1000-1010")

{

color="brown";

Outlier("SSimple",0.05);

R_Date( "OxA-12301", 3439,30)

{

Outlier ("SSimple",0.05);

};

R_Date( "OxA-12302", 3386,31)

{

Outlier ("SSimple",0.05);

};

};

Gap( 10);

R_Combine( "RY1010-1020")

{

color="brown";

Outlier("SSimple",0.05);

R_Date( "OxA-12303", 3467,31)

{

Outlier ("SSimple",0.05);

};

R_Date( "OxA-12407", 3385,34)

{

Outlier ("SSimple",0.05);

};

};

Gap( 10);

R_Combine( "RY1020-1030")

{

color="brown";

Outlier("SSimple",0.05);

R_Date( "OxA-12304", 3404,31)

{

Outlier ("SSimple",0.05);

};

R_Date( "OxA-12305", 3459,31)

{

Outlier ("SSimple",0.05);

};

};

Gap( 10);

R_Combine( "RY1030-1040")

{

color="brown";

Outlier("SSimple",0.05);

R_Date( "OxA-12306", 3416,31)

{

Outlier ("SSimple",0.05);

};

R_Date( "OxA-12307", 3425,31)

{

Outlier ("SSimple",0.05);

};

};

Gap( 10);

R_Combine( "RY1040-1050")

{

color="brown";

Outlier("SSimple",0.05);

R_Date( "OxA-12308", 3361,31)

{

Outlier ("SSimple",0.05);

};

R_Date( "OxA-12309", 3397,32)

{

Outlier ("SSimple",0.05);

};

};

Gap( 10);

R_Combine( "RY1050-1060")

{

color="brown";

Outlier("SSimple",0.05);

R_Date( "OxA-12310", 3345,32)

{

Outlier ("SSimple",0.05);

};

R_Date( "OxA-12311", 3397,32)

{

Outlier ("SSimple",0.05);

};

};

Gap( 10);

R_Combine( "RY1060-1070")

{

color="brown";

Outlier("SSimple",0.05);

R_Date( "OxA-12312", 3388,30)

{

Outlier ("SSimple",0.05);

};

R_Date( "OxA-12313", 3352,31)

{

Outlier ("SSimple",0.05);

};

};

};

D_Sequence ("Noceto")

{

R_Date("NOC-14A GrM-17548 RY995-999 @997",3380,25)

{

color="black";

Outlier("SSimple",0.05);

};

Gap(5);

R_Date("NOC-14A GrM-17645 RY1000-1004 @1002",3375,25)

{

color="black";

Outlier("SSimple",0.05);

};

Gap(50);

R_Combine ("RY1050-1054= 1052")

{

color="black";

Outlier("SSimple",0.05);

R_Date ("NOC-12-A1 GrM-11242 RY1050-1054 @1052",3320,25)

{

Outlier("SSimple",0.05);

};

R_Date ("NOC-14A GrM-13697 RY1050-1054 @1052",3274,15)

{

Outlier("SSimple",0.05);

};

};

Gap(10);

R_Date("NOC-14A GrM-13749 RY1060-1064 @1062",3332,15)

{

color="black";

Outlier ("SSimple",0.05);

};

Gap(20);

R_Combine ("RY1080-1084= 1082")

{

color="black";

Outlier("SSimple",0.05);

R_Date ("NOC-12-A2 GrM-11243 RY1080-1084 @1082",3360,25)

{

Outlier("SSimple",0.05);

};

R_Date ("NOC-14A GrM-13750 RY1080-1084 @1082",3332,15)

{

Outlier("SSimple",0.05);

};

};

Gap(20);

R_Date ("NOC-14A GrM-13751 RY1100-1104 @1102",3317,15)

{

color="black";

Outlier ("SSimple",0.05);

};

Gap(20);

R_Combine ("RY1120-1124= 1122")

{

color="black";

Outlier("SSimple",0.05);

R_Date ("NOC-12-A3 GrM-11331 RY1120-1124 @1122",3227,14)

{

Outlier("SSimple",0.05);

};

R_Date ("NOC-12A GrM-13696 RY1120-1124 @1122",3282,15)

{

Outlier("SSimple",0.05);

};

R_Date ("NOC-14A GrM-13752 RY1120-1124 @1122",3260,15)

{

Outlier("SSimple",0.05);

};

R_Date ("NOC-14A GrM-17679 RY1120-1124 @1122",3255,25)

{

Outlier("SSimple",0.05);

};

};

Gap(20);

R_Date ("NOC-14A GrM-13754 RY1140-1144 @1142",3263,15)

{

color="black";

Outlier ("SSimple",0.05);

};

Gap(20);

R_Date ("NOC-12-A4 GrM-17406 RY1160-1164 @1162",3250,25)

{

color="black";

Outlier ("SSimple",0.05);

};

Gap (20);

R_Date ("NOC-14A GrM-13755 RY1180-1184 @1182",3222,15)

{

color="black";

Outlier ("SSimple",0.05);

};

Gap(20);

R_Date ("NOC-14A GrM-17737 RY1200-1204 @1202",3160,35)

{

color="black";

Outlier ("SSimple",0.05);

};

};

};

**4. Amarna period ^14^C dating model for Fig. 4b using data previously published [refs. 7, 51].** The Amarna model (below) and shown in Fig. 4b employs a published dataset [ref. 51], with a revised OxCal model. Historical evidence indicates that Amarna was occupied from year 5 of Amenhotep IV (Akhenaten) and then through his remaining reign (died year 17) and for about 3 years under his successors (to Tutankhamun year 2). Tutankhamun subsequently died in his year 9 or 10 and was buried at Thebes [refs. 7, 34, 51]. The model for Fig. 4b assumes an ordered Sequence with the Amarna Midden samples (associated at earliest with Amenhotep IV (Akhenaten) year 5 and at latest with Tutankhamum year 2) before the short-lived Tutankhamun tomb samples. The total period represented by the dated samples is between a maximum of 24/25 years and a minimum of 8 years. The Amarna Midden samples comprise 4 samples on wood charcoal which may include in-built age and the remainder are short or shorter-lived samples. We apply the OxCal Charcoal Outlier model [ref. 24] to the wood charcoal samples to allow approximately for in-built age and apply the OxCal General Outlier model to the short/shorter-lived samples to assess for and down-weight any outliers [ref. 24]. Since the Amarna Midden samples likely represent estimates for various years/periods of time between Amenhotep IV year 5 and Tutankhamun year 2 (about 16 years in total) [refs. 7, 34, 51] we placed them in an unordered OxCal Phase. Six dates are excluded as (large) outliers—it has to be likely that they represent miss-associated samples [refs. 7, 51]. The dates from the Tutankhamun tomb likely represent a single year (we assume of the burial) following the death of Tutankhamun in his year 9 or 10 [refs. 7, 34, 51]. We combined them into a weighted average [ref. 42]. This failed a χ^2^ test [ref. 42]. One date in this 7-date set was a clear outlier using the SSimple Outlier [ref. 24] and we excluded this date (indicated in the runfile below). The total period of time from Amenhotep IV year 5 to Tutankhamun year 9/10 is believed to be ~24/25 years [refs. 34, 51]. The minimum period of time between any sample in the Amarna Midden and the Tutankhamun tomb should be ~8 years and the maximum ~25 years. We thus apply a uniform probability constraint of 8–25 years via an OxCal Interval query on the time difference between an OxCal Date estimate for the Amarna Midden (an estimate for the time between the start and end Boundaries around the Amarna Midden Phase) and the weighted average for the samples from the Tutankhamun tomb. We tested the Amarna model for a seasonal ^14^C offset using a neutral prior of 0±10 ^14^C years [ref. 24]. This found an offset across the whole model of only of 7±9 ^14^C years versus IntCal20. However, the Tutankhamun tomb dataset (n=6), placed ~1327 BCE, indicates a maximum possible offset of ~19 ^14^C years versus IntCal20 (Fig. 3a).

Options()

{

Resolution=1;

//IntCal20 default calibration file – otherwise insert call here, e.g.: Curve="IntCal20.14c";

};

Plot( )

{

Delta_R("Seasonal Test",0,10);

Outlier_Model("/",T(5),U(0,4),"r");

Outlier_Model("Charcoal",Exp(1,-10,0),U(0,3),"t");

Outlier_Model("SSimple",N(0,2),0,"s");

Sequence( )

{

Boundary("S");

Phase( "Amarna Midden Akhenaten")

{

R_Date( "Q-2401 wood", 3035, 35)

{

Outlier("Charcoal", 1);

color="blue";

};

R_Date( "Q-2402 charcoal", 3055, 35)

{

Outlier("Charcoal", 1);

color="blue";

};

R_Date( "OxA-14611 charcoal", 3066, 32)

{

Outlier("Charcoal", 1);

color="blue";

};

R_Date( "OxA-14612 wood", 3109, 31)

{

Outlier("Charcoal", 1);

color="blue";

};

//remaining samples shorter or short-lived

R_Date( "Q-2403", 3050, 35)

{

Outlier("General", 0.05);

color="blue";

};

R_Date( "Q-2404", 3025, 35)

{

Outlier("General", 0.05);

color="blue";

};

R_Date( "Q-2405", 3088, 35)

{

Outlier("General", 0.05);

color="blue";

};

R_Date( "OxA-14537", 3116, 31)

{

Outlier("General", 0.05);

color="blue";

};

R_Date( "OxA-14538", 3134, 32)

{

Outlier("General", 0.05);

color="blue";

};

R_Date( "OxA-14539", 3058, 31)

{

Outlier("General", 0.05);

color="blue";

};

R_Date( "OxA-14540", 3109, 33)

{

Outlier("General", 0.05);

color="blue";

};

R_Date( "OxA-14563", 3134, 34)

{

Outlier("General", 0.05);

color="blue";

};

R_Date( "OxA-14564", 3068, 33)

{

Outlier("General", 0.05);

color="blue";

};

R_Date( "OxA-14565", 3137, 33)

{

Outlier("General", 0.05);

color="blue";

};

R_Date("OxA-18057", 3082, 29)

{

Outlier("General", 0.05);

color="blue";

};

R_Date("OxA-18407", 3096, 28)

{

Outlier("General", 0.05);

color="blue";

};

R_Date("OxA-18953", 3092, 27)

{

Outlier("General", 0.05);

color="blue";

};

//R_Date("OxA-18954", 2976, 28)

//{

// Outlier("General", 0.05);

//};

//Outlier ~39%

R_Date("OxA-18956", 3028, 27)

{

Outlier("General", 0.05);

color="blue";

};

//R_Date("OxA-20482", 2787, 31)

//{

// Outlier("General", 0.05);

//};

//Outlier ~100%

R_Date("OxA-18512", 3051, 27)

{

Outlier("General", 0.05);

color="blue";

};

R_Date("OxA-18412", 3064, 28)

{

Outlier("General", 0.05);

color="blue";

};

//R_Date("OxA-19004", 2862, 26)

//{

// Outlier("General", 0.05);

//};

//Outlier ~100%

//R_Date("OxA-19263", 2798, 27)

//{

// Outlier("General", 0.05);

//};

//Outlier ~100%

//R_Date("VERA-4686", 2847, 36)

//{

// Outlier("General", 0.05);

//};

//Outlier ~100%

//R_Date("VERA-4686B", 2918, 30)

//{

// Outlier("General", 0.05);

//};

//Outlier ~98%

R_Date("OxA-18955", 3115, 30)

{

Outlier("General", 0.05);

color="blue";

};

R_Date("VERA-4687", 3094, 37)

{

Outlier("General", 0.05);

color="blue";

};

R_Date("VERA-4687B", 3070, 37)

{

Outlier("General", 0.05);

color="blue";

};

R_Date("VERA-4685", 3096, 34)

{

Outlier("General", 0.05);

color="blue";

};

R_Date("VERA-4685B", 3116, 35)

{

Outlier("General", 0.05);

color="blue";

};

Date("AM");

};

Boundary ("Amarna to Tutankhamun burial");

Phase ("Tutankhamun SL Thebes")

{

R_Combine ("Tut")

{

Outlier("General", 0.05);

color="orange";

R_Date("OxA-17868", 3065, 31)

{

Outlier ("SSimple",0.05);

color="orange";

};

R_Date("OxA-18950", 3138, 28)

{

Outlier ("SSimple",0.05);

color="orange";

};

R_Date("OxA-18951", 3137, 29)

{

Outlier ("SSimple",0.05);

color="orange";

};

R_Date("OxA-18952", 3117, 29)

{

Outlier ("SSimple",0.05);

color="orange";

};

R_Date("OxA-19003", 3106, 26)

{

Outlier ("SSimple",0.05);

color="orange";

};

R_Date("OxA-19132", 3133, 29)

{

Outlier ("SSimple",0.05);

color="orange";

};

//R_Date("OxA-19550", 3015, 25)

//{

// Outlier("General", 0.05);

//};

//Outlier ~99%

};

};

Boundary ("E");

};

Difference("D","Tut","AM",Interval("MinMax",U(8,25)));

};

//Outliers from preliminary runs versus IntCal20 with no Delta_R allowance

**5. Model for dating the Thera/Santorini volcanic eruption by ^14^C using an appropriate method previously published [refs. 53, 54] (see Fig. 5a).** This model regards the group of dated samples on short-lived materials from the VDL at Akrotiri on Thera as a group of events which are assumed to be distributed exponentially towards the end of the final pre-volcanic eruption phase at Akrotiri using a Tau Boundary paired with a Boundary in OxCal [ref. 24]. The exponential (Tau Boundary) model is particularly appropriate because it assumes that all the ^14^C-dated samples are older than the eruption (most probably only very slightly), but perhaps a few by a more substantial amount. This ensures that dates on individual residual samples or individual samples older for some other reason will not cause us to overestimate the age of the eruption. The end boundary “E” is thus the estimated date for the eruption. The model for Fig. 5b inserts a line “Delta_R ("Med/NE offset 8",8,0);”. The set of 24 dates employed by ref. 53 are used (see ref. [53] for explanation), with the addition of the subsequently published date on insect chitin [ref. S124].

Options()

{

Resolution=1;

//IntCal20 default calibration file – otherwise insert call here, e.g.: Curve="IntCal20.14c";

};

Plot( )

{

//Fig. 5b model insert appropriate Delta_R value here

Sequence()

{

Tau_Boundary("T");

Phase()

{

R_Date( "Hd-7092-6795", 3360, 60);

//R_Date( "Hd-5058-5519", 3490, 80);

//Date in ref. [52] set but excluded by ref. [53] in discussion

//R_Date( "Hd-6059-7967", 3140, 70);

//Date in ref. [52] set but excluded by ref. [53] in discussion

R_Date( "OxA-1552", 3390,65);

//R_Date( "OxA-1555", 3245, 65);

//Date in ref. [52] set but excluded by ref. [53] in discussion

R_Date( "OxA-1548", 3335,60);

//R_Date( "OxA-1549", 3460, 80);

//Date in ref. [52] set but excluded by ref. [53] in discussion

R_Date( "OxA-1550", 3395,65);

R_Date( "OxA-1553", 3340,65);

R_Date( "OxA-1554", 3280,65);

R_Date( "OxA-1556", 3415,70);

R_Date( "K-5352", 3310,65);

R_Date( "K-5353", 3430,90);

R_Date( "K-3228", 3340,55);

R_Date( "K-4255", 3380,60);

R_Date( "OxA-11817", 3348,31);

R_Date( "OxA-11818", 3367,33);

R_Date( "OxA-11820", 3400,31);

R_Date( "OxA-11869", 3336,34);

R_Date( "OxA-12170", 3336,28);

R_Date( "OxA-12171", 3372,28);

R_Date( "OxA-12175", 3318,28);

R_Date( "OxA-12172", 3321,32);

R_Date( "VERA-2756", 3317,28);

R_Date( "VERA-2757", 3315,31);

R_Date( "VERA-2758", 3339,28);

R_Date( "VERA-2757 repeat", 3390,32);

R_Date( "VERA-2758 repeat", 3322,33);

R_Date ("OxA-25176", 3368, 29);

//Date added from ref. S124

};

Boundary("E");

};

};

**6. Model for Thera olive branch as a Sequence in OxCal (Fig. S11)**. Data from ref. [56], see also ref. [54]. The rings recognized and used in the original study [ref. 56] are not used following subsequent critique [ref. 55]. Instead, we use an OxCal Sequence from inner-most to outer-most dated segments. We retain the ‘ring’ numbers only to help readers identify the ^14^C dates.

Options()

{

Resolution=1;

//IntCal20 default calibration file – otherwise insert call here, e.g.: Curve="IntCal20.14c";

};

Plot( )

{

//Fig. S10c model insert Delta_R("8 14C year offset test",8,0);

Sequence("Friedrich et al. 2006 Olive as Sequence Only")

{

Boundary("Start");

Sequence()

{

R_Date( "Hd-23599-24426 'rings' 1-13", 3383, 11);

R_Date( "Hd-23587 'rings' 14-37", 3372, 12);

R_Date( "Hd-23589 'rings' 38-59", 3349, 12);

R_Date( "Hd-23588-24402 'rings' 60-72", 3331, 10);

};

Boundary("End");

};

};

**7. Aegean ^14^C model for the periods before, around, and after the Thera eruptions, modifying and extending the Bayesian chronological model previously published (ref. 52), adding information, approaches and data from refs. [53, 54, 56, S109, S110, S124, S160, S164–S168]: see Fig. 6a, Supplementary Fig. S12, Table S4.** The model comprises:

1. Wiggle-matches on an earlier LM IA oak sample from Trianda, Rhodes (hence a TPQ for a point in earlier LM IA), and a mature LM IA oak sample from Miletos found covered by Theran tephra and hence a TPQ for the Thera eruption event.
2. A Sequence (from innermost to outermost) of four dated segments of an olive branch found buried by (and likely killed by) the Thera eruption [ref. 56]. Only a Sequence is used as we cannot reliably use the ‘ring’ information from such an olive sample [refs. 54, 55]. The outermost dated segment provides a TPQ (likely close) for the Thera eruption.
3. A Phase of dates comprising TPQ information, some by a long way, for the LM IA period through to dates for the earlier LMIA period and all offering TPQ information for mature LM IA and the Thera eruption. This includes a Sequence of dates from Final Neolithic, Early Cycladic, Early Middle Cycladic, though Late Middle Cycladic from Akrotiri, Thera [ref. S168] (the Sequence is based on the assigned cultural associations, see ref. [S168], and the very obvious sequence of older to more recent ^14^C ages), a Phase with other dates on pre-Late Cycladic I wood, a Sequence from Kommos on Crete comprising dates on wood charcoal from early LM IA at Kommos (in the case of one sample, K85A/62D/8:83, the two ^14^C dates on the same sample disagree and fail a χ^2^ test [ref. 42] and we arbitrarily remove the clearly much too old age estimate from the model, VERA-2638), and the Trianda early LM IA wiggle-match in (i) above and other Trianda dates [ref. S164].
4. A Phase of data related to mature LM IA. Overall, this Phase ends with, or just following, the Thera eruption. Within the Phase there is a Sequence, with a Phase of pre-VDL pre-late LM IA dates (from wood charcoal and from bone samples) before a Phase with the latest dates including the short-lived samples from the Akrotiri VDL and evidence that is either a close TPQ or approximately contemporary with the VDL and eruption. The pre-VDL Phase contains dates on bone samples from mature LM IA at Miletos, a Sequence on a small olive wood sample (treated as a Sequence given the issues over recognizing correct dendrochronological sequences in olive wood [ref. 55]), a wiggle-match on a small *Tamarix* sp. branch/twig out to bark, a suite of random Late Cycladic I dates on wood charcoal [ref. S168] (note: we include here one date listed as VDL? because the ^14^C age appears to belong with the LCI group – it is otherwise a likely a too old outlier in the VDL group), and we also assign to this Phase three LB IA/LM IA dates from Trianda on Rhodes on wood-charcoal samples which appear to belong in this grouping a TPQs for the close of LM IA and the VDL [ref. S164]. The olive sample (M4N003) had one segment, ‘rings 5-6’, where the two ^14^C age measurements on the same sample fail to agree as representing the same ^14^C age, failing a χ^2^ test [ref. 42], and so we exclude this sample and these two dates (since it is not obvious that just one of them is incorrect). All these samples will date by varying amounts before the latest stage of Late Cycladic I/LM IA and before the VDL. There then follows a Phase with samples which should date either very shortly before or around the time of the VDL at Akrotiri on Thera and so the Thera eruption. These include a Phase of dates on wood charcoal from Late Cycladic I advanced or VDL contexts, a short-lived oak twig from late LM IA Trianda in a context that was covered by Thera tephra, some cattle bones from a plausible Thera eruption tsunami context at the Palaikastro Promontory, Crete [ref. S109], and some sheep/goat remains and a tooth from a drain deposit at Palaikastro stratified with Thera tephra [ref. S110]. In the cases of the two Palaikastro contexts it is possible that the relevant animals and the dated material were killed/stratified by the Thera eruption and its impacts and so these dates could be dates for the eruption. However, it is also possible that the relevant animals had died previously and their remains became incorporated into these deposits with Thera tsunami and tephra deposits. Thus they are either close TPQs, or dates for the Thera eruption. This Phase also includes the TPQs from the Miletos oak wiggle-match from (i) above (sample found in Thera tephra covered context) and the olive branch outermost segment from the olive branch found buried on Thera and a close eruption TPQ from (ii) above. The end Boundary following this Phase of samples is regarded as equating approximately with the Thera eruption. Finally, there is the 26-date set of dates on short-lived samples from the VDL (as used by ref. [52] minus the 3 widely varying Hd dates, see discussion in ref. [33]) at Akrotiri, Thera, to which we add the ^14^C value obtained in a subsequent study on insect chitin from a VDL storage jar (charred bean weevils, *B. Rufipes*, from pithos 1, one of several large storage jars found in room 5 on the south wall of the ground floor of the West House at Akrotiri [ref. S124]). These dates are modelled as a Phase with a Tau_Boundary paired with a Boundary (see model 5 above) so that the end Boundary is regarded as an approximation for the Thera eruption which follows more or less immediately [refs. 53, 54]. This Boundary is cross-referenced with the Thera eruption Boundary from the other close TPQs to Thera eruption dates to give a dating estimate incorporating all information within the model for this event. Note: a small charcoal sample was found stratified above a deposit of Thera tephra at Pelekita Cave, Crete [ref. S95]. This sample *might* offer a *terminus ante quem* (TAQ) for the Thera eruption, but perhaps does not (relevant tree-rings could have grown earlier and the ^14^C ages could be consistent with such a suggestion) (see ref. [S95] and Supplementary Discussion 3). Accordingly, being cautious, we do *not* use the two ^14^C dates on this sample in our primary model. Including them in fact makes very little difference, see Supplementary Fig. S16.
5. The LM IB period follows LM IA on Crete [refs. S134, S137] and a phase associated with LM IB follows on Rhodes after the close of LM IA Thera tephra fall and a period of damage/abandonment and then new occupation/building [refs. S90, S92, S93]. Where present, Thera eruption products (like tephra) pre-date LM IB (see above). We however have no ^14^C data relating specifically to earlier LM IB. In fact, most of the evidence relates to close of LM IB destructions at various sites – Chania is perhaps the partial exception [refs. 52, S137, S165, S193]. The Boundary “Start LM IB” in the model is thus acting as start of mature LM IB. Work to date some ‘earlier’ LM IB contexts should be a research priority. We again use Tau_Boundaries paired with Boundaries to model the dates, all on short-lived samples, from each of the LM IB sites and their destruction horizons. Thus the end Boundary approximates the LM IB destruction for each site (the Trianda set are all on wood-charcoal samples [ref. S164], including some in-built age, and hence the relationship is less clear in this case), but the data leading to this may include some dates on pre-destruction samples. All these LM IB dates and sites date after the Thera eruption, and likely by some time. The Chania LMIB Phase floats within the LMIB Phase because the relative placement of this site’s LM IB destruction within the Cretan sequence is not well defined. We use the sequence within LM IB proposed by Rutter [ref. S198] and as used also in ref. [S165]. Thus there is a Sequence, with the LMIB Late destruction set from Myrtos-Pyrgos placed before the LM IB Final set from Mochlos.
6. The LM II period follows LM IB on Crete. We have a set of dates on short-lived samples from a close of LM II destruction context at Knossos, Crete. We again model them with a Tau_Boundary paired with a Boundary. There is also a date from Kommos on a LM II bone sample.
7. Our schematic sequence (with > indicating older than) is:
8. Pre-LM IA/LC I > (2) Earlier LM IA/LC I > (3) Mature LMIA but pre-VDL/Eruption > (4) Akrotiri VDL and contemporaries > (5) post-eruption deposits and LM IB > (6) LM II.
9. Three dates in the OxCal runfile listed below have Outlier queries applied (DEM-1455, DEM-1646, DEM-1607). The model runs including these samples (A_model_ ~65 and _Aoverall_ ~71), but these samples yield very poor individual OxCal Agreement values (respectively ~14.2, ~7.7, and ~3.8 across multiple model runs). These three dates are all on wood-charcoal samples. They stand out in their groupings as too old in two cases (thus likely and plausible residual or ‘old wood’ issues), but in one case the date is too recent (given the rest of the data and constraints of the model). We therefore applied an Outlier query as in the runfile below to each of these three dates. This removes the date from the model and assesses the probability that this date belongs in its stated place within the model. In each case the probability for these dates being in the correct position is respectively 1%, 0% and 0%. Without these three dates the overall model achieves good OxCal A_model_ and A_overall_ values, typically around 141.8 to 143.4 and 151.5 to 151.9 respectively. We therefore employ this version of the model. We note that the results for the date of the Thera eruption are similar in either case.
10. The model in total lists 153 ^14^C dates. Dates run on the same sample are combined (OxCal R_Combine) and tested for whether the dates obtained are consistent with representing the same ^14^C age [ref. 42] – two cases where this is a problem are noted above. As a result three ^14^C dates are excluded (see (iii) and (iv) above). Thus the initial model run employs 150 dates. Three further dates are identified in this initial model as exhibiting very poor individual OxCal Agreement index values, and so in a revised model (as listed below) they are excluded with Outlier queries (see (viii) above). Therefore, the preferred model employs 147 ^14^C dates. We set the kIterations value high in the Options, kIterations=3000, because in a complicated model like this with several sub-Phases and some cross-references and no obvious (only one possibility) solution in all cases, there is an increased danger otherwise of the model completing with a poor Convergence solution (but this of course means that the model takes a long time to complete). Needless to say, only outcomes with good Convergence (C) values (≥ 95) should be used. Note: even within the set of models with good Convergence, individual runs exhibit very small differences (for the example of the Thera Eruption Boundary, see Supplementary Fig. S13).

Options()

{

Resolution=1;

kIterations=3000;

//IntCal20 default calibration file – otherwise insert call here, e.g.: Curve="IntCal20.14c";

};

Plot("Thera eruption Aegean Sequence")

{

//Data from Manning et al. 2006 unless otherwise noted

//RY Relative Years, equals tree-rings

//The hypothetical +8 14C years model: insert “Delta_R(“Hypothetical Aegean Offset?”,8,0);”

D_Sequence("Trianda WM, Quercus sp.")

{

First( );

R_Combine("RY1pith-RY10 @5.5")

{

R_Date("OxA-10730", 3490, 45);

R_Date("OxA-11948", 3526, 25);

R_Date("VERA-2742", 3476, 28);

};

Gap( 10);

R_Combine("RY11-RY20 @15.5")

{

R_Date("OxA-10729", 3410, 45);

R_Date("OxA-11946", 3474, 24);

R_Date("VERA-2741", 3485, 28);

};

Gap( 10);

R_Combine("RY21-RY30bark @25.5")

{

R_Date("OxA-10728", 3455, 45);

R_Date("OxA-11945", 3473, 24);

R_Date("VERA-2740", 3481, 32);

};

Gap(4.5);

Date("Felling Trianda WM");

};

Page( );

D_Sequence("Miletos WM, Quercus sp.")

{

First( );

R_Combine("RY1000-RY1010")

{

R_Date("OxA-12301", 3439, 30);

R_Date("OxA-12302", 3386, 31);

};

Gap( 10);

R_Combine("RY1010-RY1020")

{

R_Date("OxA-12303", 3467, 31);

R_Date("OxA-12407", 3385, 34);

};

Gap( 10);

R_Combine("RY1020-RY1030")

{

R_Date("OxA-12304", 3404, 31);

R_Date("OxA-12305", 3459, 31);

};

Gap( 10);

R_Combine("RY1030-RY1040")

{

R_Date("OxA-12306", 3416, 31);

R_Date("OxA-12307", 3425, 31);

};

Gap( 10);

R_Combine("RY1040-RY1050")

{

R_Date("OxA-12308", 3361, 31);

R_Date("OxA-12309", 3397, 32);

};

Gap( 10);

R_Combine("RY1050-RY1060")

{

R_Date("OxA-12310", 3345, 32);

R_Date("OxA-12311", 3397, 32);

};

Gap( 10);

R_Combine("RY1060-RY1070")

{

R_Date("OxA-12312", 3388, 30);

R_Date("OxA-12313", 3352, 31);

};

Gap( 7);

Date("Felling Miletos WM");

};

Page( );

Sequence("Friedrich et al. 2006 Olive as Sequence Only")

{

Boundary("Start");

Sequence()

{

R_Date("Hd-23599-24426 'rings' 1-13", 3383, 11);

R_Date("Hd-23587 'rings' 14-37", 3372, 12);

R_Date("Hd-23589 'rings' 38-59", 3349, 12);

R_Date("Hd-23588-24402 'rings' 60-72", 3331, 10);

//Friedrich et al. 2006 - only Sequence, no rings used

};

Boundary("End");

};

Page();

Sequence("MBA-LB2, LCI/LMIA TPQ then LMIA-II short-lived")

{

Boundary( "Start Sequence");

Phase("MBA or LMIA long-lived = TPQ for Late LMIA")

{

Sequence("Pre-Late Cycladic, LC, DEM dates from Akrotiri, Thera")

{

Boundary("Start: Final Neolithic, FN, Early Cycladic, EC, to Early Middle Cycladic, EMC, Charcoal");

R_Date("DEM-1530 FN?/EC?", 4618, 25);

R_Date("DEM-1608 ECII", 3929, 25);

R_Date("DEM-1647 rock chamber with LMC but seems ECII/III",3840, 25);

R_Date("DEM-1312 chamber in rock, pre-LC, likely ECIII/EMC",3664, 25);

Boundary("Transition pre-Late Middle Cycladic, LMC, to LMC/pre-LCI");

Phase ("Akrotiri Charcoal from Later Middle Cycladic or pre LCI Contexts")

{

R_Date("DEM-1528 before LC?", 3462, 25);

R_Date("DEM-1531 LMC", 3441, 25);

R_Date("DEM-1623 LMC", 3499, 25);

//Data from Maniatis 2012

};

Boundary();

};

Sequence()

{

Boundary();

Phase("Akrotiri long-lived wood earlier than LCI context")

{

R_Combine("M54/2/VII/60/de>247")

{

R_Date("OxA-11250", 3550, 45);

R_Date("Hd22037", 3552, 19);

};

};

Boundary();

};

Sequence("Kommos early LMIA")

{

//Data Manning et al. 2006; see comments Manning & Bronk Ramsey 2009

Boundary("Start Kommos Early LMIA charcoal");

Phase( "Kommos early LMIA secure charcoal longer-lived, TPQs")

{

R_Combine("K85A/62D/9:92 Quercus sp.")

{

R_Date("OxA-11251", 3505, 40);

R_Date("VERA-2636", 3445, 25);

};

Phase("K85A/62D/8:83 Quercus sp.")

{

R_Date( "OxA-11253", 3397, 38);

//R_Date("VERA-2638", 3600, 19);

//Same sample but X2 22.484 > 3.8 - given other data regard VERA-2638 as too old outlier and exclude

};

R_Combine("Space 25B, Tr.66B likely Cupressaceae")

{

R_Date("OxA-11883", 3485, 33);

R_Date("OxA-11944", 3435, 25);

R_Date("OxA-3429", 3350, 70);

};

};

Boundary("longer-lived samples to shorter-lived twig (nearer likely age)");

R_Combine("K85A/66B/4:22+23 twig - so latest evidence")

{

R_Date("OxA-11252", 3375, 45);

R_Date("VERA-2637", 3390, 20);

};

Boundary("End early LMIA Kommos data");

};

Sequence()

{

Boundary();

Phase("Earlier LBIA/LMIA at Trianda,Rhodes")

{

R_Date("DEM-89",3517,83);

R_Date("DEM-859",3568,44);

};

Boundary();

//Data from Marketou et al. 2001 charcoal samples

};

After("Late MBA/Early LMIA (Trianda)")

{

Date("=Felling Trianda WM");

};

};

Boundary("Start Mature LMIA");

Phase("Mature LMIA")

{

Sequence()

{

Phase("Pre-VDL LMIA dates from LCI wood charcoal and bone samples")

{

Sequence()

{

Boundary();

Phase("Miletos LMIA bone samples")

{

R_Date("OxA-11954", 3377, 24);

R_Date("OxA-11951", 3423, 23);

};

Boundary();

};

R_Date ("DEM-1458 LMC/Early LC I charcoal",3375, 25);

//Date from Maniatis 2012

Sequence("M4N003 Olea europaea, treat as Sequence, since olive rings an insecure basis for a D_Sequence")

{

Boundary("Start M4N003");

R_Combine("Inner 'Rings' 3-4")

{

R_Date("OxA-10319", 3424, 38);

R_Date("VERA-2747", 3386, 30);

};

R_Combine("Next 'Rings' 3-5")

{

R_Date("OxA-10316", 3342, 38);

R_Date("VERA-2744", 3427, 31);

};

//R_Combine("Next 'Rings' 5-6")

//{

// R_Date("OxA-10318", 3355, 40);

// R_Date("VERA-2746", 3471, 28);

//};

//X2 fails, 5.616 > 3.8, thus exclude this pair of dates on same sample

R_Combine("Next 'Rings' 6-8Bark")

{

R_Date("OxA-10315", 3446, 39);

R_Date("VERA-2743", 3413, 28);

};

R_Combine("Outermost 'Rings' 7-8Bark")

{

R_Date("OxA-10317", 3440, 35);

R_Date("VERA-2745", 3386, 28);

};

};

D_Sequence("65/N001/I2 Tamarix sp.")

{

R_Combine("Ring 1")

{

R_Date("OxA-10314", 3330, 27);

R_Date("VERA-2751", 3325, 28);

};

R_Combine("Ring 2")

{

R_Date("OxA-10313", 3353, 27);

R_Date("VERA-2749", 3335, 33);

};

R_Combine("65/N001/I2 Ring 3 Bark")

{

R_Date("OxA-10312", 3293, 27);

R_Date("VERA-2748", 3319, 28);

};

};

Sequence()

{

Boundary();

Phase ("Akrotiri Charcoal LCI")

{

R_Date ("DEM-1313 LC I", 3396, 25);

R_Date ("DEM-1314 LC I", 3467, 25);

R_Date ("DEM-1345 LC I", 3441, 25);

R_Date ("DEM-1455 LC I", 3508, 30)

{

Outlier();

};

R_Date ("DEM-1456 LC I", 3456, 25);

R_Date ("DEM-1457 LC I", 3436, 25);

R_Date ("DEM-1609 LC I", 3433, 25);

R_Date ("DEM-1610 LC I", 3420, 25);

R_Date ("DEM-1646 LC I", 3508, 25)

{

Outlier();

};

R_Date ("DEM-1615 LC I Advanced VDL?", 3389,25);

//Published as VDL? Have assigned to LCI as 14C age is outlier, too old, for VDL whereas fits as LCI

};

Boundary();

//Data from Maniatis 2012

//The two outliers are too old and thus plausibly really later MBA or earlier LCI TPQ wood-charcoal

};

Sequence()

{

Boundary();

Phase("Mature LBIA/LMIA charcoal from Trianda, Rhodes, clearly TPQ for VDL")

{

R_Date ("DEM-828", 3407,25);

R_Date ("DEM-830", 3449,21);

R_Date ("DEM-831", 3466,23);

};

Boundary();

//Data from Marketou et al. 2001. Dates on charcoal from mature LBIA/LMIA but clearly TPQ for VDL

};

};

Boundary ("Transition to Immediate VDL TPQs and VDL");

Phase ("Specific TPQs to VDL or VDL")

{

Sequence()

{

Boundary();

Phase()

{

Sequence()

{

Boundary();

Phase("LCI Advanced or Advanced? or VDL charcoal, Akrotiri, Thera")

{

R_Date ("DEM-1311 LC I VDL", 3307, 25);

R_Date ("DEM-1529 LC I Advanced? VDL", 3281,25);

R_Date ("DEM-1607 LC I Advanced VDL", 3228,30)

{

Outlier();

};

R_Date ("DEM-1624 LC I Advanced VDL", 3360,25);

};

Boundary();

//Data from Maniatis 2012

//One outlier that is too recent

};

Sequence()

{

Boundary();

Phase("LBIA/LMIA mature and destruction later below Thera tephra, Trianda, Rhodes")

{

R_Date ("DEM-94", 3347,46);

R_Date ("DEM-93", 3358,48);

};

Boundary();

//Data from Marketou et al. 2001

};

R_Combine( "Trianda short-lived late LMIA twig, pre-Thera-tephra, Quercus sp.")

{

R_Date( "OxA-10643", 3367, 39);

R_Date( "OxA-11884", 3344, 32);

};

R_Combine("Cattle bones Palaikastro Promontory Thera tsunami?")

{

R_Date("GrA-30336 Cattle bone",3310,35);

R_Date("GrA-30339 Cattle bone",3390,35);

//Bruins et al. 2008; 2009

//Animals could have died before tephra, or because of eruption impact - thus in Phase as shortly before, to contemporary with, eruption.

};

R_Combine("Bone/jaw, tooth Palaikastro drain with stratified Thera tephra")

{

R_Date("GrA-30336 goat/sheep bone/jaw",3325,40);

R_Date("GrA-30339 goat/sheep bone/jaw",3345,40);

R_Date("GrA-29042 tooth",3385,40);

//Bruins et al. 2009

//Animals could have died before tephra, or because of eruption impact - thus in Phase as shortly before, to contemporary with, eruption.

};

After("Miletos Thera eruption TPQ")

{

Date("=Felling Miletos WM");

};

After("Olive branch last dated Segment, Thera eruption TPQ")

{

Date("=Hd-23588-24402 'rings' 60-72");

};

};

Boundary("Thera Eruption");

};

Sequence("Akrotiri VDL short-lived samples")

{

Tau_Boundary("T1");

Phase("VDL 25 date set")

{

R_Date( "OxA-1552", 3390, 65);

R_Date( "OxA-1555", 3245, 65);

R_Date( "OxA-1548", 3335, 60);

R_Date( "OxA-1549", 3460, 80);

R_Date( "OxA-1550", 3395, 65);

R_Date( "OxA-1553", 3340, 65);

R_Date( "OxA-1554", 3280, 65);

R_Date( "OxA-1556", 3415, 70);

R_Date( "K-5352", 3310, 65);

R_Date( "K-5353", 3430, 90);

R_Date( "K-3228", 3340, 55);

R_Date( "K-4255", 3380, 60);

R_Date( "OxA-11817", 3348, 31);

R_Date( "OxA-11818", 3367, 33);

R_Date( "OxA-11820", 3400, 31);

R_Date( "OxA-11869", 3336, 34);

R_Date( "OxA-12170", 3336, 28);

R_Date( "OxA-12171", 3372, 28);

R_Date( "OxA-12175", 3318, 28);

R_Date( "OxA-12172", 3321, 32);

R_Date( "VERA-2756", 3317, 28);

R_Date( "VERA-2757", 3315, 31);

R_Date( "VERA-2758", 3339, 28);

R_Date( "VERA-2757 repeat", 3390, 32);

R_Date( "VERA-2758 repeat", 3322, 33);

R_Date ("OxA-25176 insect chitin", 3368, 29);

};

Boundary("=Thera Eruption");

};

};

};

Interval("Interval Mature LMIA");

};

Boundary("Post-Eruption, End LMIA through to Earlier LMIB");

Interval("Interval not represented, Post-Eruption Final/End LMIA to Earlier LMIB");

Boundary("Start LMIB");

Phase("LMIB")

{

Sequence("LMIB Trianda, Rhodes, charcoal TPQ")

{

Boundary();

Phase()

{

R_Date("DEM-90",3258,54);

R_Date("DEM-91",3240,35);

R_Date("DEM-856",3175,41);

R_Date("DEM-829",3171,33);

R_Date("DEM-857",3142,52);

R_Date("DEM-858",3138,50);

};

//Data from Marketou et al. 2001

Boundary();

};

Sequence ("Chania (LMIB Early? to Late)")

{

Tau_Boundary ("T2");

Phase ("Chania, charred seeds")

{

R_Date("OxA-2517", 3380, 80);

R_Date("OxA-2518", 3340, 80);

R_Date("OxA-2646", 3315, 70);

R_Date("OxA-2647", 3150, 70);

R_Date("OxA-10320", 3208, 26);

R_Date("OxA-10321", 3268, 27);

R_Date("OxA-10322", 3338, 26);

R_Date("OxA-10323", 3253, 25);

};

Boundary ("Chania LMIB Destruction, later LMIB");

};

Sequence ("LMIB Late to LMIB Final Destructions")

{

Sequence( "Myrtos-Pyrgos (LMIB Late) Destruction")

{

Tau_Boundary ("T3");

Phase ("Myrtos-Pyrgos, charred seeds")

{

R_Date("OxA-3187", 3230, 70);

R_Date("OxA-3188", 3200, 70);

R_Date("OxA-3189", 3270, 70);

R_Date("OxA-3225", 3160, 80);

R_Date("OxA-10324", 3270, 26);

R_Date("OxA-10325", 3228, 26);

R_Date("OxA-10326", 3227, 25);

R_Date("OxA-10411", 3150, 40);

};

Boundary ("Myrtos-Pyrgos Destruction, LMIB Late to LMIB Final");

};

Sequence( "Mochlos (LMIB Final)")

{

Tau_Boundary ("T4");

Phase ("Mochlos Olive stones")

{

R_Date("Beta-85991", 3240, 50);

R_Date("Beta-85992", 3180, 40);

R_Date("Beta-115890", 3170, 60);

R_Date("Beta-129765", 3220, 40);

R_Date("Beta-151768", 3270, 40);

//Data and LMIB Late-Final Sequence after Manning 2009

};

Boundary ("Mochlos Destruction, LMIB Final to LMII Transition");

};

};

};

Boundary("LMIB Final to LMII");

Phase ("LMII")

{

Sequence("Knossos LMII Destruction short-lived")

{

Tau_Boundary ("T5");

Phase ("Knossos LMII Destruction, charred seeds")

{

R_Date("OxA-2096", 3070, 70);

R_Date("OxA-2097", 3190, 65);

R_Date("OxA-2098", 3220, 65);

R_Date("OxA-11882", 3156, 33);

R_Date("OxA-11943", 3148, 23);

};

Boundary ("Knossos LM II Destruction");

};

R_Date("OxA-3674 Kommos LMII bone", 3090, 80);

};

Boundary("End Sequence");

};

};

**8. The Aegean model in 7. but excluding all the data from Thera (Supplementary Fig. S15)**

Options()

{

Resolution=1;

kIterations=3000;

//IntCal20 default calibration file – otherwise insert call here, e.g.: Curve="IntCal20.14c";

};

Plot("Thera eruption Aegean Sequence")

{

//Data from Manning et al. 2006 unless otherwise noted

//RY Relative Years, equals tree-rings

//The hypothetical +8 14C years model: insert “Delta_R(“Hypothetical Aegean Offset?”,8,0);”

D_Sequence("Trianda WM, Quercus sp.")

{

First( );

R_Combine("RY1pith-RY10 @5.5")

{

R_Date("OxA-10730", 3490, 45);

R_Date("OxA-11948", 3526, 25);

R_Date("VERA-2742", 3476, 28);

};

Gap( 10);

R_Combine("RY11-RY20 @15.5")

{

R_Date("OxA-10729", 3410, 45);

R_Date("OxA-11946", 3474, 24);

R_Date("VERA-2741", 3485, 28);

};

Gap( 10);

R_Combine("RY21-RY30bark @25.5")

{

R_Date("OxA-10728", 3455, 45);

R_Date("OxA-11945", 3473, 24);

R_Date("VERA-2740", 3481, 32);

};

Gap(4.5);

Date("Felling Trianda WM");

};

Page( );

D_Sequence("Miletos WM, Quercus sp.")

{

First( );

R_Combine("RY1000-RY1010")

{

R_Date("OxA-12301", 3439, 30);

R_Date("OxA-12302", 3386, 31);

};

Gap( 10);

R_Combine("RY1010-RY1020")

{

R_Date("OxA-12303", 3467, 31);

R_Date("OxA-12407", 3385, 34);

};

Gap( 10);

R_Combine("RY1020-RY1030")

{

R_Date("OxA-12304", 3404, 31);

R_Date("OxA-12305", 3459, 31);

};

Gap( 10);

R_Combine("RY1030-RY1040")

{

R_Date("OxA-12306", 3416, 31);

R_Date("OxA-12307", 3425, 31);

};

Gap( 10);

R_Combine("RY1040-RY1050")

{

R_Date("OxA-12308", 3361, 31);

R_Date("OxA-12309", 3397, 32);

};

Gap( 10);

R_Combine("RY1050-RY1060")

{

R_Date("OxA-12310", 3345, 32);

R_Date("OxA-12311", 3397, 32);

};

Gap( 10);

R_Combine("RY1060-RY1070")

{

R_Date("OxA-12312", 3388, 30);

R_Date("OxA-12313", 3352, 31);

};

Gap( 7);

Date("Felling Miletos WM");

};

Page( );

// Sequence("Friedrich et al. 2006 Olive as Sequence Only")

// {

// Boundary("Start");

// Sequence()

// {

// R_Date("Hd-23599-24426 'rings' 1-13", 3383, 11);

// R_Date("Hd-23587 'rings' 14-37", 3372, 12);

// R_Date("Hd-23589 'rings' 38-59", 3349, 12);

// R_Date("Hd-23588-24402 'rings' 60-72", 3331, 10);

//Friedrich et al. 2006 - only Sequence, no rings used

// };

// Boundary("End");

// };

Page();

Sequence("MBA-LB2, LCI/LMIA TPQ then LMIA-II short-lived")

{

Boundary( "Start Sequence");

Phase("MBA or LMIA long-lived = TPQ for Late LMIA")

{

//Sequence("Pre-Late Cycladic, LC, DEM dates from Akrotiri, Thera")

//{

// Boundary("Start: Final Neolithic, FN, Early Cycladic, EC, to Early Middle Cycladic, EMC, Charcoal");

// R_Date("DEM-1530 FN?/EC?", 4618, 25);

// R_Date("DEM-1608 ECII", 3929, 25);

// R_Date("DEM-1647 rock chamber with LMC but seems ECII/III",3840, 25);

// R_Date("DEM-1312 chamber in rock, pre-LC, likely ECIII/EMC",3664, 25);

// Boundary("Transition pre-Late Middle Cycladic, LMC, to LMC/pre-LCI");

// Phase ("Akrotiri Charcoal from Later Middle Cycladic or pre LCI Contexts")

// {

// R_Date("DEM-1528 before LC?", 3462, 25);

// R_Date("DEM-1531 LMC", 3441, 25);

// R_Date("DEM-1623 LMC", 3499, 25);

//Data from Maniatis 2012

// };

// Boundary();

//};

// Sequence()

// {

// Boundary();

// Phase("Akrotiri long-lived wood earlier than LCI context")

// {

// R_Combine("M54/2/VII/60/de>247")

// {

// R_Date("OxA-11250", 3550, 45);

// R_Date("Hd22037", 3552, 19);

// };

// };

// Boundary();

// };

Sequence("Kommos early LMIA")

{

//Data Manning et al. 2006; see comments Manning & Bronk Ramsey 2009

Boundary("Start Kommos Early LMIA charcoal");

Phase( "Kommos early LMIA secure charcoal longer-lived, TPQs")

{

R_Combine("K85A/62D/9:92 Quercus sp.")

{

R_Date("OxA-11251", 3505, 40);

R_Date("VERA-2636", 3445, 25);

};

Phase("K85A/62D/8:83 Quercus sp.")

{

R_Date( "OxA-11253", 3397, 38);

//R_Date("VERA-2638", 3600, 19);

//Same sample but X2 22.484 > 3.8 - given other data regard VERA-2638 as too old outlier and exclude

};

R_Combine("Space 25B, Tr.66B likely Cupressaceae")

{

R_Date("OxA-11883", 3485, 33);

R_Date("OxA-11944", 3435, 25);

R_Date("OxA-3429", 3350, 70);

};

};

Boundary("longer-lived samples to shorter-lived twig (nearer likely age)");

R_Combine("K85A/66B/4:22+23 twig - so latest evidence")

{

R_Date("OxA-11252", 3375, 45);

R_Date("VERA-2637", 3390, 20);

};

Boundary("End early LMIA Kommos data");

};

Sequence()

{

Boundary();

Phase("Earlier LBIA/LMIA at Trianda,Rhodes")

{

R_Date("DEM-89",3517,83);

R_Date("DEM-859",3568,44);

};

Boundary();

//Data from Marketou et al. 2001 charcoal samples

};

After("Late MBA/Early LMIA (Trianda)")

{

Date("=Felling Trianda WM");

};

};

Boundary("Start Mature LMIA");

Phase("Mature LMIA")

{

Sequence()

{

Phase("Pre-VDL LMIA dates from LCI wood charcoal and bone samples")

{

Sequence()

{

Boundary();

Phase("Miletos LMIA bone samples")

{

R_Date("OxA-11954", 3377, 24);

R_Date("OxA-11951", 3423, 23);

};

Boundary();

};

//R_Date ("DEM-1458 LMC/Early LC I charcoal",3375, 25);

//Date from Maniatis 2012

//Sequence("M4N003 Olea europaea, treat as Sequence, since olive rings an insecure basis for a D_Sequence")

//{

// Boundary("Start M4N003");

// R_Combine("Inner 'Rings' 3-4")

// {

// R_Date("OxA-10319", 3424, 38);

// R_Date("VERA-2747", 3386, 30);

// };

// R_Combine("Next 'Rings' 3-5")

// {

// R_Date("OxA-10316", 3342, 38);

// R_Date("VERA-2744", 3427, 31);

// };

//R_Combine("Next 'Rings' 5-6")

//{

// R_Date("OxA-10318", 3355, 40);

// R_Date("VERA-2746", 3471, 28);

//};

//X2 fails, 5.616 > 3.8, thus exclude this pair of dates on same sample

// R_Combine("Next 'Rings' 6-8Bark")

// {

// R_Date("OxA-10315", 3446, 39);

// R_Date("VERA-2743", 3413, 28);

// };

// R_Combine("Outermost 'Rings' 7-8Bark")

// {

// R_Date("OxA-10317", 3440, 35);

// R_Date("VERA-2745", 3386, 28);

// };

// };

//D_Sequence("65/N001/I2 Tamarix sp.")

//{

// R_Combine("Ring 1")

// {

// R_Date("OxA-10314", 3330, 27);

// R_Date("VERA-2751", 3325, 28);

// };

// R_Combine("Ring 2")

// {

// R_Date("OxA-10313", 3353, 27);

// R_Date("VERA-2749", 3335, 33);

// };

// R_Combine("65/N001/I2 Ring 3 Bark")

// {

// R_Date("OxA-10312", 3293, 27);

// R_Date("VERA-2748", 3319, 28);

// };

// };

// Sequence()

// {

// Boundary();

// Phase ("Akrotiri Charcoal LCI")

// {

// R_Date ("DEM-1313 LC I", 3396, 25);

// R_Date ("DEM-1314 LC I", 3467, 25);

// R_Date ("DEM-1345 LC I", 3441, 25);

// R_Date ("DEM-1455 LC I", 3508, 30)

// {

// Outlier();

// };

// R_Date ("DEM-1456 LC I", 3456, 25);

// R_Date ("DEM-1457 LC I", 3436, 25);

// R_Date ("DEM-1609 LC I", 3433, 25);

// R_Date ("DEM-1610 LC I", 3420, 25);

// R_Date ("DEM-1646 LC I", 3508, 25)

// {

// Outlier();

// };

// R_Date ("DEM-1615 LC I Advanced VDL?", 3389,25);

//Published as VDL? Have assigned to LCI as 14C age is outlier, too old, for VDL whereas fits as LCI

// };

// Boundary();

//Data from Maniatis 2012

//The two outliers are too old and thus plausibly really later MBA or earlier LCI TPQ wood-charcoal

//};

Sequence()

{

Boundary();

Phase("Mature LBIA/LMIA charcoal from Trianda, Rhodes, clearly TPQ for VDL")

{

R_Date ("DEM-828", 3407,25);

R_Date ("DEM-830", 3449,21);

R_Date ("DEM-831", 3466,23);

};

Boundary();

//Data from Marketou et al. 2001. Dates on charcoal from mature LBIA/LMIA but clearly TPQ for VDL

};

};

Boundary ("Transition to Immediate VDL TPQs and VDL");

Phase ("Specific TPQs to VDL or VDL")

{

Sequence()

{

Boundary();

Phase()

{

//Sequence()

// {

// Boundary();

// Phase("LCI Advanced or Advanced? or VDL charcoal, Akrotiri, Thera")

// {

// R_Date ("DEM-1311 LC I VDL", 3307, 25);

// R_Date ("DEM-1529 LC I Advanced? VDL", 3281,25);

// R_Date ("DEM-1607 LC I Advanced VDL", 3228,30)

// {

// Outlier();

// };

// R_Date ("DEM-1624 LC I Advanced VDL", 3360,25);

//};

//Boundary();

//Data from Maniatis 2012

//One outlier that is too recent

//};

Sequence()

{

Boundary();

Phase("LBIA/LMIA mature and destruction later below Thera tephra, Trianda, Rhodes")

{

R_Date ("DEM-94", 3347,46);

R_Date ("DEM-93", 3358,48);

};

Boundary();

//Data from Marketou et al. 2001

};

R_Combine( "Trianda short-lived late LMIA twig, pre-Thera-tephra, Quercus sp.")

{

R_Date( "OxA-10643", 3367, 39);

R_Date( "OxA-11884", 3344, 32);

};

R_Combine("Cattle bones Palaikastro Promontory Thera tsunami?")

{

R_Date("GrA-30336 Cattle bone",3310,35);

R_Date("GrA-30339 Cattle bone",3390,35);

//Bruins et al. 2008; 2009

//Animals could have died before tephra, or because of eruption impact - thus in Phase as shortly before, to contemporary with, eruption.

};

R_Combine("Bone/jaw, tooth Palaikastro drain with stratified Thera tephra")

{

R_Date("GrA-30336 goat/sheep bone/jaw",3325,40);

R_Date("GrA-30339 goat/sheep bone/jaw",3345,40);

R_Date("GrA-29042 tooth",3385,40);

//Bruins et al. 2009

//Animals could have died before tephra, or because of eruption impact - thus in Phase as shortly before, to contemporary with, eruption.

};

After("Miletos Thera eruption TPQ")

{

Date("=Felling Miletos WM");

};

// After("Olive branch last dated Segment, Thera eruption TPQ")

// {

// Date("=Hd-23588-24402 'rings' 60-72");

// };

};

Boundary("Thera Eruption");

};

//Sequence("Akrotiri VDL short-lived samples")

//{

// Tau_Boundary("T1");

//Phase("VDL 25 date set")

//{

// R_Date( "OxA-1552", 3390, 65);

// R_Date( "OxA-1555", 3245, 65);

// R_Date( "OxA-1548", 3335, 60);

// R_Date( "OxA-1549", 3460, 80);

// R_Date( "OxA-1550", 3395, 65);

// R_Date( "OxA-1553", 3340, 65);

// R_Date( "OxA-1554", 3280, 65);

// R_Date( "OxA-1556", 3415, 70);

// R_Date( "K-5352", 3310, 65);

// R_Date( "K-5353", 3430, 90);

// R_Date( "K-3228", 3340, 55);

// R_Date( "K-4255", 3380, 60);

// R_Date( "OxA-11817", 3348, 31);

// R_Date( "OxA-11818", 3367, 33);

// R_Date( "OxA-11820", 3400, 31);

// R_Date( "OxA-11869", 3336, 34);

// R_Date( "OxA-12170", 3336, 28);

// R_Date( "OxA-12171", 3372, 28);

// R_Date( "OxA-12175", 3318, 28);

// R_Date( "OxA-12172", 3321, 32);

// R_Date( "VERA-2756", 3317, 28);

// R_Date( "VERA-2757", 3315, 31);

// R_Date( "VERA-2758", 3339, 28);

// R_Date( "VERA-2757 repeat", 3390, 32);

// R_Date( "VERA-2758 repeat", 3322, 33);

// R_Date ("OxA-25176 insect chitin", 3368, 29);

//};

//Boundary("=Thera Eruption");

//};

};

};

Interval("Interval Mature LMIA");

};

Boundary("Post-Eruption, End LMIA through to Earlier LMIB");

Interval("Interval not represented, Post-Eruption Final/End LMIA to Earlier LMIB");

Boundary("Start LMIB");

Phase("LMIB")

{

Sequence("LMIB Trianda, Rhodes, charcoal TPQ")

{

Boundary();

Phase()

{

R_Date("DEM-90",3258,54);

R_Date("DEM-91",3240,35);

R_Date("DEM-856",3175,41);

R_Date("DEM-829",3171,33);

R_Date("DEM-857",3142,52);

R_Date("DEM-858",3138,50);

};

//Data from Marketou et al. 2001

Boundary();

};

Sequence ("Chania (LMIB Early? to Late)")

{

Tau_Boundary ("T2");

Phase ("Chania, charred seeds")

{

R_Date("OxA-2517", 3380, 80);

R_Date("OxA-2518", 3340, 80);

R_Date("OxA-2646", 3315, 70);

R_Date("OxA-2647", 3150, 70);

R_Date("OxA-10320", 3208, 26);

R_Date("OxA-10321", 3268, 27);

R_Date("OxA-10322", 3338, 26);

R_Date("OxA-10323", 3253, 25);

};

Boundary ("Chania LMIB Destruction, later LMIB");

};

Sequence ("LMIB Late to LMIB Final Destructions")

{

Sequence( "Myrtos-Pyrgos (LMIB Late) Destruction")

{

Tau_Boundary ("T3");

Phase ("Myrtos-Pyrgos, charred seeds")

{

R_Date("OxA-3187", 3230, 70);

R_Date("OxA-3188", 3200, 70);

R_Date("OxA-3189", 3270, 70);

R_Date("OxA-3225", 3160, 80);

R_Date("OxA-10324", 3270, 26);

R_Date("OxA-10325", 3228, 26);

R_Date("OxA-10326", 3227, 25);

R_Date("OxA-10411", 3150, 40);

};

Boundary ("Myrtos-Pyrgos Destruction, LMIB Late to LMIB Final");

};

Sequence( "Mochlos (LMIB Final)")

{

Tau_Boundary ("T4");

Phase ("Mochlos Olive stones")

{

R_Date("Beta-85991", 3240, 50);

R_Date("Beta-85992", 3180, 40);

R_Date("Beta-115890", 3170, 60);

R_Date("Beta-129765", 3220, 40);

R_Date("Beta-151768", 3270, 40);

//Data and LMIB Late-Final Sequence after Manning 2009

};

Boundary ("Mochlos Destruction, LMIB Final to LMII Transition");

};

};

};

Boundary("LMIB Final to LMII");

Phase ("LMII")

{

Sequence("Knossos LMII Destruction short-lived")

{

Tau_Boundary ("T5");

Phase ("Knossos LMII Destruction, charred seeds")

{

R_Date("OxA-2096", 3070, 70);

R_Date("OxA-2097", 3190, 65);

R_Date("OxA-2098", 3220, 65);

R_Date("OxA-11882", 3156, 33);

R_Date("OxA-11943", 3148, 23);

};

Boundary ("Knossos LM II Destruction");

};

R_Date("OxA-3674 Kommos LMII bone", 3090, 80);

};

Boundary("End Sequence");

};

};

**9. The Aegean model in 7. but adding in the Pelekita Cave TAQ (Supplementary Fig. S16)**

Options()

{

Resolution=1;

kIterations=3000;

//IntCal20 default calibration file – otherwise insert call here, e.g.: Curve="IntCal20.14c";

};

Plot("Thera eruption Aegean Sequence")

{

//Data from Manning et al. 2006 unless otherwise noted

//RY Relative Years, equals tree-rings

//The hypothetical +8 14C years model: insert “Delta_R(“Hypothetical Aegean Offset?”,8,0);”

D_Sequence("Trianda WM, Quercus sp.")

{

First( );

R_Combine("RY1pith-RY10 @5.5")

{

R_Date("OxA-10730", 3490, 45);

R_Date("OxA-11948", 3526, 25);

R_Date("VERA-2742", 3476, 28);

};

Gap( 10);

R_Combine("RY11-RY20 @15.5")

{

R_Date("OxA-10729", 3410, 45);

R_Date("OxA-11946", 3474, 24);

R_Date("VERA-2741", 3485, 28);

};

Gap( 10);

R_Combine("RY21-RY30bark @25.5")

{

R_Date("OxA-10728", 3455, 45);

R_Date("OxA-11945", 3473, 24);

R_Date("VERA-2740", 3481, 32);

};

Gap(4.5);

Date("Felling Trianda WM");

};

Page( );

D_Sequence("Miletos WM, Quercus sp.")

{

First( );

R_Combine("RY1000-RY1010")

{

R_Date("OxA-12301", 3439, 30);

R_Date("OxA-12302", 3386, 31);

};

Gap( 10);

R_Combine("RY1010-RY1020")

{

R_Date("OxA-12303", 3467, 31);

R_Date("OxA-12407", 3385, 34);

};

Gap( 10);

R_Combine("RY1020-RY1030")

{

R_Date("OxA-12304", 3404, 31);

R_Date("OxA-12305", 3459, 31);

};

Gap( 10);

R_Combine("RY1030-RY1040")

{

R_Date("OxA-12306", 3416, 31);

R_Date("OxA-12307", 3425, 31);

};

Gap( 10);

R_Combine("RY1040-RY1050")

{

R_Date("OxA-12308", 3361, 31);

R_Date("OxA-12309", 3397, 32);

};

Gap( 10);

R_Combine("RY1050-RY1060")

{

R_Date("OxA-12310", 3345, 32);

R_Date("OxA-12311", 3397, 32);

};

Gap( 10);

R_Combine("RY1060-RY1070")

{

R_Date("OxA-12312", 3388, 30);

R_Date("OxA-12313", 3352, 31);

};

Gap( 7);

Date("Felling Miletos WM");

};

Page( );

Sequence("Friedrich et al. 2006 Olive as Sequence Only")

{

Boundary("Start");

Sequence()

{

R_Date("Hd-23599-24426 'rings' 1-13", 3383, 11);

R_Date("Hd-23587 'rings' 14-37", 3372, 12);

R_Date("Hd-23589 'rings' 38-59", 3349, 12);

R_Date("Hd-23588-24402 'rings' 60-72", 3331, 10);

//Friedrich et al. 2006 - only Sequence, no rings used

};

Boundary("End");

};

Page();

Sequence("MBA-LB2, LCI/LMIA TPQ then LMIA-II short-lived")

{

Boundary( "Start Sequence");

Phase("MBA or LMIA long-lived = TPQ for Late LMIA")

{

Sequence("Pre-Late Cycladic, LC, DEM dates from Akrotiri, Thera")

{

Boundary("Start: Final Neolithic, FN, Early Cycladic, EC, to Early Middle Cycladic, EMC, Charcoal");

R_Date("DEM-1530 FN?/EC?", 4618, 25);

R_Date("DEM-1608 ECII", 3929, 25);

R_Date("DEM-1647 rock chamber with LMC but seems ECII/III",3840, 25);

R_Date("DEM-1312 chamber in rock, pre-LC, likely ECIII/EMC",3664, 25);

Boundary("Transition pre-Late Middle Cycladic, LMC, to LMC/pre-LCI");

Phase ("Akrotiri Charcoal from Later Middle Cycladic or pre LCI Contexts")

{

R_Date("DEM-1528 before LC?", 3462, 25);

R_Date("DEM-1531 LMC", 3441, 25);

R_Date("DEM-1623 LMC", 3499, 25);

//Data from Maniatis 2012

};

Boundary();

};

Sequence()

{

Boundary();

Phase("Akrotiri long-lived wood earlier than LCI context")

{

R_Combine("M54/2/VII/60/de>247")

{

R_Date("OxA-11250", 3550, 45);

R_Date("Hd22037", 3552, 19);

};

};

Boundary();

};

Sequence("Kommos early LMIA")

{

//Data Manning et al. 2006; see comments Manning & Bronk Ramsey 2009

Boundary("Start Kommos Early LMIA charcoal");

Phase( "Kommos early LMIA secure charcoal longer-lived, TPQs")

{

R_Combine("K85A/62D/9:92 Quercus sp.")

{

R_Date("OxA-11251", 3505, 40);

R_Date("VERA-2636", 3445, 25);

};

Phase("K85A/62D/8:83 Quercus sp.")

{

R_Date( "OxA-11253", 3397, 38);

//R_Date("VERA-2638", 3600, 19);

//Same sample but X2 22.484 > 3.8 - given other data regard VERA-2638 as too old outlier and exclude

};

R_Combine("Space 25B, Tr.66B likely Cupressaceae")

{

R_Date("OxA-11883", 3485, 33);

R_Date("OxA-11944", 3435, 25);

R_Date("OxA-3429", 3350, 70);

};

};

Boundary("longer-lived samples to shorter-lived twig (nearer likely age)");

R_Combine("K85A/66B/4:22+23 twig - so latest evidence")

{

R_Date("OxA-11252", 3375, 45);

R_Date("VERA-2637", 3390, 20);

};

Boundary("End early LMIA Kommos data");

};

Sequence()

{

Boundary();

Phase("Earlier LBIA/LMIA at Trianda,Rhodes")

{

R_Date("DEM-89",3517,83);

R_Date("DEM-859",3568,44);

};

Boundary();

//Data from Marketou et al. 2001 charcoal samples

};

After("Late MBA/Early LMIA (Trianda)")

{

Date("=Felling Trianda WM");

};

};

Boundary("Start Mature LMIA");

Phase("Mature LMIA")

{

Sequence()

{

Phase("Pre-VDL LMIA dates from LCI wood charcoal and bone samples")

{

Sequence()

{

Boundary();

Phase("Miletos LMIA bone samples")

{

R_Date("OxA-11954", 3377, 24);

R_Date("OxA-11951", 3423, 23);

};

Boundary();

};

R_Date ("DEM-1458 LMC/Early LC I charcoal",3375, 25);

//Date from Maniatis 2012

Sequence("M4N003 Olea europaea, treat as Sequence, since olive rings an insecure basis for a D_Sequence")

{

Boundary("Start M4N003");

R_Combine("Inner 'Rings' 3-4")

{

R_Date("OxA-10319", 3424, 38);

R_Date("VERA-2747", 3386, 30);

};

R_Combine("Next 'Rings' 3-5")

{

R_Date("OxA-10316", 3342, 38);

R_Date("VERA-2744", 3427, 31);

};

//R_Combine("Next 'Rings' 5-6")

//{

// R_Date("OxA-10318", 3355, 40);

// R_Date("VERA-2746", 3471, 28);

//};

//X2 fails, 5.616 > 3.8, thus exclude this pair of dates on same sample

R_Combine("Next 'Rings' 6-8Bark")

{

R_Date("OxA-10315", 3446, 39);

R_Date("VERA-2743", 3413, 28);

};

R_Combine("Outermost 'Rings' 7-8Bark")

{

R_Date("OxA-10317", 3440, 35);

R_Date("VERA-2745", 3386, 28);

};

};

D_Sequence("65/N001/I2 Tamarix sp.")

{

R_Combine("Ring 1")

{

R_Date("OxA-10314", 3330, 27);

R_Date("VERA-2751", 3325, 28);

};

R_Combine("Ring 2")

{

R_Date("OxA-10313", 3353, 27);

R_Date("VERA-2749", 3335, 33);

};

R_Combine("65/N001/I2 Ring 3 Bark")

{

R_Date("OxA-10312", 3293, 27);

R_Date("VERA-2748", 3319, 28);

};

};

Sequence()

{

Boundary();

Phase ("Akrotiri Charcoal LCI")

{

R_Date ("DEM-1313 LC I", 3396, 25);

R_Date ("DEM-1314 LC I", 3467, 25);

R_Date ("DEM-1345 LC I", 3441, 25);

R_Date ("DEM-1455 LC I", 3508, 30)

{

Outlier();

};

R_Date ("DEM-1456 LC I", 3456, 25);

R_Date ("DEM-1457 LC I", 3436, 25);

R_Date ("DEM-1609 LC I", 3433, 25);

R_Date ("DEM-1610 LC I", 3420, 25);

R_Date ("DEM-1646 LC I", 3508, 25)

{

Outlier();

};

R_Date ("DEM-1615 LC I Advanced VDL?", 3389,25);

//Published as VDL? Have assigned to LCI as 14C age is outlier, too old, for VDL whereas fits as LCI

};

Boundary();

//Data from Maniatis 2012

//Two outliers are too old and thus plausibly really later MBA or earlier LCI TPQ wood-charcoal

};

Sequence()

{

Boundary();

Phase("Mature LBIA/LMIA charcoal from Trianda, Rhodes, clearly TPQ for VDL")

{

R_Date ("DEM-828", 3407,25);

R_Date ("DEM-830", 3449,21);

R_Date ("DEM-831", 3466,23);

};

Boundary();

//Data from Marketou et al. 2001. Dates on charcoal from mature LBIA/LMIA but clearly TPQ for VDL

};

};

Boundary ("Transition to Immediate VDL TPQs and VDL");

Phase ("Specific TPQs to VDL or VDL")

{

Sequence()

{

Boundary();

Phase()

{

Sequence()

{

Boundary();

Phase("LCI Advanced or Advanced? or VDL charcoal, Akrotiri, Thera")

{

R_Date ("DEM-1311 LC I VDL", 3307, 25);

R_Date ("DEM-1529 LC I Advanced? VDL", 3281,25);

R_Date ("DEM-1607 LC I Advanced VDL", 3228,30)

{

Outlier();

};

R_Date ("DEM-1624 LC I Advanced VDL", 3360,25);

};

Boundary();

//Data from Maniatis 2012

//One outlier that is too recent

};

Sequence()

{

Boundary();

Phase("LBIA/LMIA mature and destruction later below Thera tephra, Trianda, Rhodes")

{

R_Date ("DEM-94", 3347,46);

R_Date ("DEM-93", 3358,48);

};

Boundary();

//Data from Marketou et al. 2001

};

R_Combine( "Trianda short-lived late LMIA twig, pre-Thera-tephra, Quercus sp.")

{

R_Date( "OxA-10643", 3367, 39);

R_Date( "OxA-11884", 3344, 32);

};

R_Combine("Cattle bones Palaikastro Promontory Thera tsunami?")

{

R_Date("GrA-30336 Cattle bone",3310,35);

R_Date("GrA-30339 Cattle bone",3390,35);

//Bruins et al. 2008; 2009

//Animals could have died before tephra, or because of eruption impact - thus in Phase as shortly before, to contemporary with, eruption.

};

R_Combine("Bone/jaw, tooth Palaikastro drain with stratified Thera tephra")

{

R_Date("GrA-30336 goat/sheep bone/jaw",3325,40);

R_Date("GrA-30339 goat/sheep bone/jaw",3345,40);

R_Date("GrA-29042 tooth",3385,40);

//Bruins et al. 2009

//Animals could have died before tephra, or because of eruption impact - thus in Phase as shortly before, to contemporary with, eruption.

};

After("Miletos Thera eruption TPQ")

{

Date("=Felling Miletos WM");

};

After("Olive branch last dated Segment, Thera eruption TPQ")

{

Date("=Hd-23588-24402 'rings' 60-72");

};

};

Boundary("Thera Eruption");

};

Sequence("Akrotiri VDL short-lived samples")

{

Tau_Boundary("T1");

Phase("VDL 25 date set")

{

R_Date( "OxA-1552", 3390, 65);

R_Date( "OxA-1555", 3245, 65);

R_Date( "OxA-1548", 3335, 60);

R_Date( "OxA-1549", 3460, 80);

R_Date( "OxA-1550", 3395, 65);

R_Date( "OxA-1553", 3340, 65);

R_Date( "OxA-1554", 3280, 65);

R_Date( "OxA-1556", 3415, 70);

R_Date( "K-5352", 3310, 65);

R_Date( "K-5353", 3430, 90);

R_Date( "K-3228", 3340, 55);

R_Date( "K-4255", 3380, 60);

R_Date( "OxA-11817", 3348, 31);

R_Date( "OxA-11818", 3367, 33);

R_Date( "OxA-11820", 3400, 31);

R_Date( "OxA-11869", 3336, 34);

R_Date( "OxA-12170", 3336, 28);

R_Date( "OxA-12171", 3372, 28);

R_Date( "OxA-12175", 3318, 28);

R_Date( "OxA-12172", 3321, 32);

R_Date( "VERA-2756", 3317, 28);

R_Date( "VERA-2757", 3315, 31);

R_Date( "VERA-2758", 3339, 28);

R_Date( "VERA-2757 repeat", 3390, 32);

R_Date( "VERA-2758 repeat", 3322, 33);

R_Date ("OxA-25176 insect chitin", 3368, 29);

};

Boundary("=Thera Eruption");

};

};

Before("Post-Eruption TAQ Pelekita Cave, small charcoal sample")

{

R_Combine("GrA-48438a+b")

{

R_Date("GrA-48438a charcoal",3385,40);

R_Date("GrA-48438b charcoal",3345,40);

};

//Bruins et al. 2019

};

};

Interval("Interval Mature LMIA");

};

Boundary("Post-Eruption, End LMIA through to Earlier LMIB");

Interval("Interval not represented, Post-Eruption Final LMIB to Earlier LMIB");

Boundary("Start LMIB");

Phase("LMIB")

{

Sequence("LMIB Trianda, Rhodes, charcoal TPQ")

{

Boundary();

Phase()

{

R_Date("DEM-90",3258,54);

R_Date("DEM-91",3240,35);

R_Date("DEM-856",3175,41);

R_Date("DEM-829",3171,33);

R_Date("DEM-857",3142,52);

R_Date("DEM-858",3138,50);

};

//Data from Marketou et al. 2001

Boundary();

};

Sequence ("Chania (LMIB Early? to Late)")

{

Tau_Boundary ("T2");

Phase ("Chania, charred seeds")

{

R_Date("OxA-2517", 3380, 80);

R_Date("OxA-2518", 3340, 80);

R_Date("OxA-2646", 3315, 70);

R_Date("OxA-2647", 3150, 70);

R_Date("OxA-10320", 3208, 26);

R_Date("OxA-10321", 3268, 27);

R_Date("OxA-10322", 3338, 26);

R_Date("OxA-10323", 3253, 25);

};

Boundary ("Chania LMIB Destruction, later LMIB");

};

Sequence ("LMIB Late to LMIB Final Destructions")

{

Sequence( "Myrtos-Pyrgos (LMIB Late) Destruction")

{

Tau_Boundary ("T3");

Phase ("Myrtos-Pyrgos, charred seeds")

{

R_Date("OxA-3187", 3230, 70);

R_Date("OxA-3188", 3200, 70);

R_Date("OxA-3189", 3270, 70);

R_Date("OxA-3225", 3160, 80);

R_Date("OxA-10324", 3270, 26);

R_Date("OxA-10325", 3228, 26);

R_Date("OxA-10326", 3227, 25);

R_Date("OxA-10411", 3150, 40);

};

Boundary ("Myrtos-Pyrgos Destruction, LMIB Late to LMIB Final");

};

Sequence( "Mochlos (LMIB Final)")

{

Tau_Boundary ("T4");

Phase ("Mochlos Olive stones")

{

R_Date("Beta-85991", 3240, 50);

R_Date("Beta-85992", 3180, 40);

R_Date("Beta-115890", 3170, 60);

R_Date("Beta-129765", 3220, 40);

R_Date("Beta-151768", 3270, 40);

//Data and LMIB Late-Final Sequence after Manning 2009

};

Boundary ("Mochlos Destruction, LMIB Final to LMII Transition");

};

};

};

Boundary("LMIB Final to LMII");

Phase ("LMII")

{

Sequence("Knossos LMII Destruction short-lived")

{

Tau_Boundary ("T5");

Phase ("Knossos LMII Destruction, charred seeds")

{

R_Date("OxA-2096", 3070, 70);

R_Date("OxA-2097", 3190, 65);

R_Date("OxA-2098", 3220, 65);

R_Date("OxA-11882", 3156, 33);

R_Date("OxA-11943", 3148, 23);

};

Boundary ("Knossos Destruction");

};

R_Date("OxA-3674 Kommos LMII bone", 3090, 80);

};

Boundary("End Sequence");

};

};

**Supplementary Table S4.** Part A. Selected modelled calendar age ranges at 68.3% and 95.4% hpd from Supplementary Fig. S12. For the model shown in Supplementary Fig. S12 (and Fig. 6a), the A_model_ value is 142.5 and the A_overall_ value is 151.8. Across a set of 11 runs these values varied around 141.8–143.4 and 151.5–151.9 respectively and the specific modelled ages varied by up to a few years, while remaining very similar to those listed below. Part 2. As Part B but from the Fig. 6b model with hypothetical +8 ^14^C years offset. Model in Fig. 6b: A_model_=154.9, A_overall_=151.7.

| **PART A** | **68.3% hpd range(s) BCE** | **95.4% hpd range(s) BCE** |
| --- | --- | --- |
| **Boundary Start Mature LM IA** | 1728–1711 | 1738–1703 |
| **Felling Miletos WM** | 1661–1648 | 1669–1640 |
| **Last dated segment olive branch** | 1621–1612 | 1627–1598 (92.3%)  1591–1589 (0.2%)  1584–1579 (0.8%)  1578–1567 (2.1%) |
| **Boundary Thera Eruption**  **(see Fig. 6a)** | 1617–1602 (64.7%)  1567–1565 (3.5%) | 1619–1596 (72.6%)  1576–1545 (22.9%) |
| ***Interval Mature LM IA (years)*** | *97–137 years (64.5%)*  *159–164 years (3.7%)* | *93–188 years* |
| **Boundary Post-Eruption, End LM IA through to Earlier LM IB** | 1615–1590 (63.3%)  1564–1558 (5.0%) | 1616–1538 |
| ***Interval not represented, post-Eruption Final LM IA to Earlier LM IB (years)*** | *24–102 years* | *0–122 years* |
| **Boundary Chania LM IB Destruction, Later LM IB** | 1500–1452 | 1515–1422 |
| **Boundary Myrtos-Pyrgos Destruction, LM IB Late to LM IB Final** | 1498–1487 (16.1%)  1478–1451 (51.2%) | 1503–1439 |
| **Boundary Mochlos Destruction, LM IB Final to LM II Transition** | 1466–1429 | 1487–1406 |
| **Boundary Knossos Destruction LM II** | 1429–1361 (65.0%) | 1440–1256 |

| **PART B +8 ^14^C years offset** | **68.3% hpd range(s) BCE** | **95.4% hpd range(s) BCE** |
| --- | --- | --- |
| **Boundary Start Mature LM IA** | 1729–1711 | 1736–1703 |
| **Felling Miletos WM** | 1660–1647 | 1670–1630 |
| **Last dated segment olive branch** | 1620–1598 (64.5%)  1591-1589 (2.3%)  1575-1573 (1.6%) | 1622-1562 |
| **Boundary Thera Eruption**  **(see Fig. 6b)** | 1573–1557 (51.2%)  1553–1544 (17.1%) | 1612–1597 (8.2%)  1584–1538 (87.2%) |
| ***Interval Mature LM IA (years)*** | *148–188 years* | *112–208 years* |
| **Boundary Post-Eruption, End LM IA through to Earlier LM IB** | 1569–1536 | 1605–1518 |
| ***Interval not represented, post-Eruption Final LM IA to Earlier LM IB (years)*** | *5–61 years* | *0–95 years* |
| **Boundary Chania LM IB Destruction, Later LM IB** | 1494–1445 | 1500–1417 |
| **Boundary Myrtos-Pyrgos Destruction, LM IB Late to LM IB Final** | 1481–1445 | 1503–1434 |
| **Boundary Mochlos Destruction, LM IB Final to LM II Transition** | 1461–1424 | 1481–1401 |
| **Boundary Knossos Destruction LM II** | 1425–1356 (63.9%)  1328-1318 (4.3%) | 1435–1246 |

**Supplementary References S1-S198**

1. ISG. An inter-laboratory comparison of radiocarbon measurements in tree-rings. *Nature* **198**, 619–623 (1982).
2. Stuiver M. 1982. A high precision calibration of the AD radiocarbon time scale. Radiocarbon **24**, 1–26 (1982).
3. Scott, E.M., Naysmith, P. & Cook, G.T. Why do we need ^14^C inter-comparisons?: the Glasgow-^14^C inter-comparison series, a reflection over 30 years. *Quaternary Geochronology* **43**, 72–82 (2018).
4. Stuiver, M. et al. IntCal98 radiocarbon age calibration, 24,000–0 cal BP. *Radiocarbon* **40**, 1041–1083 (1998).
5. Scott, E.M., Cook, G.T., Naysmith, P. & Staff, R.A. Learning from the wood samples in ICS, TIRI, FIRI, VIRI and SIRI. *Radiocarbon* **61**, 1293–1304 (2019).
6. Scott, E.M., Naysmith, P. & Cook, G.T. Life after SIRI—where next? *Radiocarbon* **61**, 1159–1168 (2019).
7. Taylor, R.E. & Southon, J. Reviewing the mid-first millennium BC ^14^C “warp” using ^14^C/bristlecone pine data. *Nuclear Instruments and Methods in Physics Research B* **294**, 440–443 (2013).
8. Fritts, H.C. *Tree rings and climate* (Academic Press, New York, 1976).
9. Schulze, E.D., Mooney, H.A. & Dunn, E.L. Wintertime Photosynthesis of Bristlecone Pine (*Pinus aristata*) In the White Mountains of California. *Ecology* **48**, 1044–1047 (1967).
10. Harkness, D.D. & Burleigh, R. Possible carbon-14 enrichment in high altitude wood. *Archaeometry* **16**, 121–127 (1974).
11. Cain, W.F. & Suess, H.E. Carbon 14 in tree rings. *Journal of Geophysical Research* **81**, 3688–3694 (1976).
12. Leavitt, S.W. & Long, A. Altitudinal differences in δ^13^C of bristlecone pine tree rings. *Naturwissenschaften* **79**, 178–180 (1992).
13. De Boer, H.J. et al. Tree-ring isotopes suggest atmospheric drying limits temperature-growth responses of treeline bristlecone pine. *Tree Physiology* 39, 983–999 (2019).
14. Hoper, S.T., McCormac, F.G., Hogg, A.G., Higham, T.F.G. & Head, M.J. Evaluation of wood pretreatments on oak and cedar. *Radiocarbon* **40**, 45–50 (1998).
15. Staff, R.A., Reynard, L., Brock, F. & Bronk Ramsey, C. Wood pretreatment protocols and measurement of tree-rings standards at the Oxford Radiocarbon Accelerator Unit (ORAU). *Radiocarbon* **56**, 709–715 (2014).
16. Dee, M.W. et al. Radiocarbon dating at Groningen: new and updated chemical pretreatment procedures. *Radiocarbon* **62**, 63–74 (2020).
17. Stuiver, M. & Braziunas, T.F. Modelling atmospheric ^14^C influences and ^14^C ages of Marine samples to 10,000 BC. *Radiocarbon* **35**, 137–189 (1993).
18. Knox, F.B. & McFadden, B.G. Radiocarbon/tree-ring calibration, solar activity, and upwelling of ocean water. *Radiocarbon* **46**, 987–995 (2004).
19. Reimer, P.J. & Reimer, R.W. A marine reservoir correction database and on-line interface. *Radiocarbon* **43**, 461–463 (2001).
20. Hong W. et al. Calibration curve from AD 1250 to 1650 by measurements of tree-rings grown on the Korean Peninsula. *Nuclear Instruments and Methods in Physics Research B* **294**, 435–439 (2013).
21. Nakamura, T., Masuda, K., Miyake, F., Nagaya, K. & Yoshimitsu, T. Radiocarbon ages of annual rings from Japanese wood: evident age offset based on IntCal09. *Radiocarbon* **55**, 763–770 (2013).
22. Sheppard, P.R., Comrie, A.C., Packin, G.D., Angersbach, K. & Hughes, M.K. The climate of the US Southwest. *Climate Research* **21**, 219–238 (2002).
23. Roach, L.D., Charles, C.D, Field, D.B. & Guilderson, T.P. Foraminiferal radiocarbon record of northeast Pacific decadal subsurface variability. *Journal of Geophysical Research* **118**, 4317–4333 (2013).
24. Rafter, P.A. et al. Eastern tropical North Pacific coral radiocarbon reveals North Pacific Gyre Oscillation (NPGO) variability. *Quaternary Science Reviews* **160**, 108–115 (2017).
25. Di Lorenzo E. et al. **Synthesis of Pacific Ocean climate and ecosystem dynamics.** Progress of Oceanography **26**, 68–81 (2013).
26. Bond, G. et al. Persistent solar influence on North Atlantic climate during the Holocene. *Science* **294**, 2130–2136 (2001).
27. Isono, D. et al. The 1500-year climate oscillation in the mid latitude North Pacific during the Holocene. *Geology* **37**, 591–594 (2009).
28. Helama, S., Jones, P.D. & Briffa, K.R. Dark Ages Cold Period: a literature review and directions for future research. *The Holocene* **27**, 1600–1606 (2017).
29. Berkelhammer, M. & Stott, L. Recent and dramatic changes in Pacific storm trajectories recorded in δ^18^O from bristlecone pine ring cellulose. *Geochemistry, Geophysics, Geosystems* **9**, Q04008, doi:10.1029/2007GC001803 (2008).
30. Bale, R.J. et al. Temporal stability in bristlecone pine tree-ring stable isotope chronologies over the last two centuries. *The Holocene* **20**, 3–6 (2010).
31. Berkelhammer, M. & Stott, L. Correction to “Recent and dramatic changes in Pacific storm trajectories as recorded in the δ^18^O of bristlecone pine tree ring cellulose. *Geochemistry, Geophysics, Geosystems* **12**, Q09002, doi:10.1029/2011GC003765 (2011).
32. Bale, R.J. et al. An annually resolved bristlecone pine carbon isotope chronology for the last millennium. *Quaternary Research* **76**, 22–29 (2011).
33. Kuniholm, P.I., Kromer, B., Manning, S.W., Newton, M. Latini, C.E. & Bruce, M.J. Anatolian tree rings and the absolute chronology of the eastern Mediterranean, 2220-718 BC. *Nature* **381**: 780–783 (1996).
34. Newton, M.W. & Kuniholm, P.I. 2004. A Dendrochronological Framework for the Assyrian Colony Period in Asia Minor. TÜBA*-*AR **7**, 165–176 (2004).
35. Baillie, M.G.L. Suck-in and smear. Two related chronological problems for the 90s. *Journal of Theoretical Archaeology* **2**, 12–16 (1991).
36. Johnston, E.N., Sparks, R.S.J., Phillips, J.C. & Carey, S. Revised estimates for the volume of the Late Bronze Age Minoan eruption, Santorini, Greece. *Journal of the Geological Society, London* **171**, 583–590 (2014).
37. Fouqué, F.A. *Santorin et ses éruptions* (G. Masson, Paris, 1879).
38. Driessen, J. & Macdonald, C.F. *The troubled island: Minoan Crete before and after the Santorini eruption* (Aegaeum 17, Université de Liège and University of Texas at Austin, Liège, 1997).
39. Druitt, T.H. et al. *Santorini Volcano* (Geological Society Memoir 19, Geological Society, London, 1999).
40. Manning, S.W. *A Test of Time: the volcano of Thera and the chronology and history of the Aegean and east Mediterranean in the mid-second millennium BC* (Oxbow Books, Oxford, 1999).
41. Friedrich, W.L. *Santorini: volcano, natural history, mythology* (Aarhus University Press, Aarhus, 2009).
42. Warburton, D.A. (ed.) *Time’s Up! Dating the Minoan eruption of Santorini* (Monographs of the Danish Institute at Athens 10, The Danish Institute at Athens, Athens, 2009).
43. Meller, H., Bertemes, F., Bork, H.-R. & Risch, R. (eds.) *1600 – Kultureller Umbruch im Schatten des Thera-Ausbruches?/1600 – Cultural change in the shadow of the Thera-Eruption?* (Landesmuseum für Vorgeschichte, Halle, 2013).
44. Doumas, C. *Thera: Pompeii of the ancient Aegean* (Thames & Hudson, London*,* 1983).
45. Forsyth, P.Y.  *Thera in the Bronze Age* (Peter Lang, New York, 1998).
46. Doumas, C. *The wall-paintings of Thera* (The Thera Foundation-Petros M. Nomikos, Athens, 1992).
47. Palyvou, C. *Akrotiri Thera: An Architecture of Affluence 3,500 Years Old* (INSTAP Academic Press, Philadelphia, 2005).
48. Oppenheimer, C. *Eruptions that shook the world* (Cambridge University Press, Cambridge, 2011).
49. Doumas, C.G., Palyvou, C., Devetzi, A. & Bouliotis, C. *Akrotiri, Thera 17^th^ century BC: a cosmopolitan town 3500 years ago* (Society for the Promotion of Studies on Prehistoric Thera, Athens, 2015).
50. Figuier, L. *La terre et les mers* (Librairie Hachette, Paris, 4^th^ ed., 1872).
51. Wilson, I. *The Exodus Enigma* (Weidenfeld and Nicholson, London, 1985).
52. Bruins, H.J. & van der Plicht, J. The Exodus enigma. *Nature* **382**, 213–214 (1996).
53. Rehak, P. & Younger, J.G. Review of Aegean prehistory VII: Neopalatial, Final Palatial, and Postpalatial Crete. *American Journal of Archaeology* **102**, 91–173 (1998).
54. Marinatos, S. The volcanic destruction of Minoan Crete. *Antiquity* **13**, 425–439 (1939).
55. Page, D.L. *The Santorini volcano and the desolation of Minoan Crete* (Society for the Promotion of Hellenic Studies, London, 1970).
56. Driessen, J. & MacDonald, C.F. The eruption of the Santorini volcano and its effects on Minoan Crete. In W.J. McGuire, D.R. Griffiths, P.L. Hancock & I.S. Stewart (eds.), *The Archaeology of Geological Catastrophes*. p. 81–93 (Geological Society Special Publications 171, Geological Society of London, London, 2000).
57. Niemeier, W.-D. Die Auswirkungen der Thera-Eruption im ägäischen Raum. In H. Meller, F. Bertemes, H.-R. Bork & R. Risch (eds.), *1600 – Kultureller Umbruch im Schatten des Thera-Ausbruches?/1600 – Cultural change in the shadow of the Thera-Eruption?* p. 177–190 (Landesmuseum für Vorgeschichte, Halle, 2013).
58. Driessen, J. The Santorini eruption. An archaeological investigation of its distal impacts on Minoan Crete. *Quaternary International* **499**, 195–204 (2019).
59. Hankey, V. & Warren, P. The absolute chronology of the Aegean Late Bronze Age. *Bulletin of the Institute of Classical Studies* **21**, 142–152 (1974).
60. Cadogan, G. Dating the Aegean Bronze Age without radiocarbon. *Archaeometry* **20**, 209–214 (1978).
61. LaMarche, V.C. Jr. & Hirschboeck, K.K. Frost rings in trees as records of major volcanic eruptions. *Nature* **307**, 121–126 (1984).
62. Hammer, C.U., Clausen, H.B., Friedrich, W.L. & Tauber, H. The Minoan eruption of Santorini in Greece dated to 1645 BC? *Nature* **328**, 517–519 (1987).
63. Baillie, M.G.L. & Munro, M.A.R. Irish tree rings, Santorini and volcanic dust veils. *Nature* **332**, 344–346 (1988).
64. Hughes, M.K. Ice layer dating of the eruption of Santorini. *Nature* **335**, 211–212 (1988).
65. Manning, S.W. Dating of the Santorini eruption. *Nature* **332**, 401 (1988).
66. Manning, S.W. The Bronze Age eruption of Thera: absolute dating, Aegean chronology and Mediterranean cultural interrelations. *Journal of Mediterranean Archaeology* **1**, 17–82 (1988).
67. Manning, S.W. The Thera eruption: the Third Congress and the problem of the date. *Archaeometry* **32**, 91–100 (1990).
68. Bronk Ramsey, C., Manning, S.W. & Galimberti, M. Dating the volcanic eruption at Thera. *Radiocarbon* **46**, 325–344 (2004).
69. Bietak, M. Recent discussions about the chronology of the Middle and the Late Bronze Age in the eastern Mediterranean: Part I. *Bibliotheca Orientalis* **72**, 317–335 (2015).
70. Cadogan, G. Unsteady date of a big bang. *Nature* **328**, 473 (1987).
71. Hardy, D.A. & Renfrew, A.C. (eds.) *Thera and the Aegean world III. Volume three: chronology* (The Thera Foundation, London, 1990).
72. Antiquity. 2014. Debate feature: Bronze Age catastrophe and modern controversy: dating the Santorini eruption. *Antiquity* 88, 267–291 (2014).
73. Hammer, C.U., Kurat, G., Hoppe, P., Grum, W. & Clausen, H.B. Thera eruption date 1645 BC confirmed by new ice core data? In M. Bietak (ed.), *The synchronization of civilisations in the eastern Mediterranean in the second millennium B.C. II.* p. 87–94 (Österreichischen Akademie der Wissenschaften, Wien, 2003).
74. Pearce, N., Westgate, J., Preece, S., Eastwood, W. & Perkins, W. Identification of Aniakchak (Alaska) tephra in Greenland ice core challenges the 1645 BC date for Minoan eruption of Santorini. *Geochemistry, Geophysics, Geosystems* 5(3) Q03005, doi: 10.1029/2003GC000672.
75. Sarantakou, E. & Terkenli, T.S. Non-institutionalized forms of tourism accommodation and overtourism impacts on the landscape: the case of Santorini, Greece. *Tourism Planning & Development* **16**, 411–433 (2019).
76. Reck, H. *Santorin: Der Werdegang eines Inselvulkans und sein Ausbruch 1925–1928*. (Reinser, Berlin, 1936).
77. Bond, A. & Sparks, R.S.J. The Minoan eruption of Santorini, Greece. *Journal of the Geological Society, London* **132**, 1–16 (1976).
78. Heiken, G. & McCoy, F.W. Caldera development during the Minoan eruption, Thira, Cyclades, Greece. *Journal of Geophysical Research* **89**, B10, 8441–8462 (1984).
79. Druitt, T.H., Mellors, R.A., Pyle, D.M. & Sparks, R.S.J. Explosive volcanism on Santorini, Greece. *Geological Magazine* **126**, 95–126 (1985).
80. Sigurdsson, H. *et al*. Marine investigations of Greece’s Santorini volcanic field. *EOS Transactions, American Geophysical Union* **87**, 337–348 (2006).
81. Druitt, T.H., McCoy, F.W. & Vougioukalakis, G.E. The Late Bronze Age eruption of Santorini volcano and its impact on the ancient Mediterranean world. *Elements* **15**, 185–190 (2019).
82. Watkins, N.D. et al. Volume and extent of the Minoan tephra from the Santorini volcano: new evidence from deep sea cores. *Nature* **271**, 122–126 (1978).
83. Sullivan, D.G. 1990. Minoan Tephra in Lake Sediments in Western Turkey: Dating the Eruption and Assessing the Atmospheric Dispersal of the Ash. In D.A. Hardy & A.C. Renfrew (eds.), *Thera and the Aegean World III. Vol. 3: Chronology*. p. 114–119 (The Thera Foundation, London, 1990).
84. McCoy, F.W. & Heiken, G. The Late Bronze Age explosive eruption of Thera (Santorini), Greece: Regional and local effects. In F.W. McCoy & G. Heiken (eds.), *Volcanic Hazards and Disasters in Human Antiquity*. p. 43–70 (Geological Society of America Special Paper 345, Geological society of America, Boulder, 2000).
85. Zanchetta, G. et al. Tephrostratigraphy, chronology and climatic events of the Mediterranean basin during the Holocene: an overview. *The Holocene* **21**, 33–52 (2011).
86. Sulpizio, R., Alçiçek, M.C., Zanchetta, G. & Solari, L. Recognition of the Minoan tephra in the Acigöl Basin, western Turkey: implications for inter-archive correlations and fine ash dispersal. *Journal of Quaternary Science* 28: 329–335 (2013).
87. Johnston, E.N., Phillips, J.C., Bonadonna, C. & Watson, I.M. Reconstructing the tephra dispersal pattern from the Bronze Age eruption of Santorini using an advection-diffusion model. *Bulletin of Volcanology* **74**, 1485–1507 (2012).
88. Satow, C. et al. A new contribution to the Late Quaternary tephrostratigraphy of the Mediterranean: Aegean Sea core LC21. Quaternary Science Reviews **117**, 96–112 (2015).
89. Blong, R.J., *Volcanic hazards: a sourcebook on the effects of eruptions* (Academic Press, Sydney, 1984).
90. Doumas, C. & Papazoglou, L. Santorini tephra from Rhodes. *Nature* 287, 322–324 (1980).
91. Momigliano, N. Iasos and the Aegean islands before the Santorini eruption. In R. Laffineur & E. Greco (eds.), *Emporia: Aegeans in the Central and Eastern Mediterranean*. p. 217–227 (Aegaeum 25, Université de Liège and University of Texas at Austin, Liège, 2005).
92. Marketou, T. Santorini tephra from Rhodes and Kos: some chronological remarks based on stratigraphy. In D.A. Hardy & A.C. Renfrew (eds.), *Thera and the Aegean world III. Volume three: chronology*. p. 100–113 (The Thera Foundation, London).
93. Marketou, T. 1998. Excavations at Trianda (Ialysos) on Rhodes: New Evidence for the Late Bronze Age I Period. *Atti dell’Accademia nazionale dei Lincei: Rendiconti* **9**, 39–82 (1998).
94. Siart, C. et al. Karst depressions as geoarchaeological archives: the palaeoenvironmental reconstruction of Zominthos (Central Crete), based on geophysical prospection, sedimentological investigations and GIS. *Quaternary International* **216**, 75–92 (2010).
95. Bruins, H.J. et al. Tephra in caves: distal deposits of the Minoan Santorini eruption and the Campanian super-eruption. *Quaternary International* **499**, 135–147 (2019).
96. Vitaliano, C.J. & Vitaliano, D.B. Volcanic Tephra on Crete. *American Journal of Archaeology* **78**, 19–24 (1974).
97. Soles, J.S., Taylor, S.R. & Vitaliano, C.J. Tephra Samples from Mochlos and their Chronological Implications for Neopalatial Crete. *Archaeometry* **37**, 385–393 (1995).
98. Molloy, B.P.C., McCoy, F.W., Megarry, R., Govantes Edwards, D.J. & Pavlacký, M. Of tephra and tsunamis: a secondary deposit of tephra sealing LM IA activity at Priniatikos Pyrgos. In B.P.C. Molloy & C.N. Duckworth (eds.), *A Cretan landscape through time: Priniatikos Pyrgos and environs*. p. 43–53 (BAR International Series 2634, Archaeopress, Oxford, 2014).
99. Thorarinsson, S. On the damage caused by volcanic eruptions with special reference to tephra and gases. In P.D. Sheets & D.K. Grayson (eds.), *Volcanic activity and human ecology*. p. 125–160 (Academic Press, New York, 1979).
100. Cronin, S.J., Hedley, M.J., Neall, V.E. & Smith, R.G. Agronomic impact of ash fallout from the 1995 and 1996 Ruapehu Volcano eruptions, New Zealand. *Environmental Geology* 34, 21–30 (1998).
101. Cronin, S.J., Neall, V.E., Lecointre, J.A., Hedley, M.J. & Loganathan, P. Environmental hazards of fluoride in volcanic ash: a case study from Ruapehu volcano, New Zealand. *Journal of Volcanology and Geothermal Research* **121**, 271–291 (2003).
102. Riede, F. Doing palaeo-social volcanology: Developing a framework for systematically investigating the impacts of past volcanic eruptions on human societies using archaeological datasets. *Quaternary International* **499**, 266–277 (2019).
103. Minoura, K. et al. Discovery of Minoan Tsunami Deposits. *Geology* **28**, 59–62 (2000).
104. McCoy, F.W. & Heiken, G. Tsunami generated by the Late Bronze Age eruption of Thera (Santorini), Greece. *Pure and Applied Geophysics* **157**, 1227–1256 (2000).
105. Goodman-Tchernov, B.N., Dey, H.W., Reinhardt, E.G., McCoy, F. & Mart, Y. Tsunami waves generated by the Santorini eruption reached Eastern Mediterranean shores. *Geology* **37**, 943–946 (2009).
106. Novikova, T., Papadopoulos, G.A. & McCoy, F.W. Modelling of tsunami generated by the giant Late Bronze Age eruption of Thera, South Aegean Sea, Greece. *Geophysics Journal International* **186**, 665–680 (2011).
107. Periáñez, R. & Abril, J.M. Modelling tsunamis in the Eastern Mediterranean Sea. Application to the Minoan Santorini tsunami sequence as a potential scenario for the biblical Exodus. *Journal of Marine Systems* **139**, 91–102 (2014).
108. Nomikou, P. et al. Post-eruptive flooding of Santorini caldera and implications for tsunami generation. *Nature Communications* 7, 13332, doi.org/10.1038/ncomms13332 (2016)
109. Bruins, H.J. et al. Geoarchaeological tsunami deposits at Palaikastro (Crete) and the Late Minoan IA eruption of Santorini. *Journal of Archaeological Science* **35**, 191–212 (2008).
110. Bruins, H.J., van der Plicht, J. & MacGillivray, J.A. The Minoan Santorini eruption and tsunami deposits in Palaikastro (Crete): dating by geology, archaeology, ^14^C, and Egyptian chronology. *Radiocarbon* **51**, 397–411 (2009).
111. Werner, V. et al. Mid-Holocene tectonic geomorphology of northern Crete deduced from a coastal sedimentary archive near Rethymnon and a Late Bronze Age Santorini tsunamite candidate. *Geomorphology* **326**, 167–189 (2019).
112. Dominey-Howes, D. Documentary and geological records of tsunamis in the Aegean Sea region of Greece and their potential value to risk assessment and disaster management. *Natural Hazards* **25**, 195–224 (2002).
113. Bottema, S. & Sarpaki, A. Environmental change in Crete: a 9000-year record of Holocene vegetation history and the effect of the Santorini eruption. *The Holocene* **13**, 733–749 (2003).
114. Eastwood, W.L., Tibby, J., Roberts, N., Birks, H.J.B. & Lamb, H.F. The environmental impact of the Minoan eruption of Santorini (Thera): statistical analysis of palaeoecological data from Gölhisar, southwest Turkey. *The Holocene* **12**, 431–444 (2002).
115. Manning, S.W. & Sewell, D.A. Volcanoes and history: a significant relationship? The case of Thera. In R. Torrence & J. Grattan (eds.), *Natural disasters and cultural change*. p. 264–291 (Routledge, London, 2002).
116. Sigurdsson, Carey, S. & Devine, J.D. Assessment of mass, dynamics and environmental effects of the Minoan eruption of Santorini volcano. In D.A. Hardy, J. Keller, V.P. Galanopoulos, N.C. Flemming & T.H. Druitt (eds.), *Thera and the Aegean world III. Volume two: earth sciences*. p. 100–112 (The Thera Foundation, London, 1990).
117. Pyle, D.M. New volume estimates for the Minoan eruption. In D.A. Hardy, J. Keller, V.P. Galanopoulos, N.C. Flemming & T.H. Druitt (eds.), *Thera and the Aegean world III. Volume two: earth sciences*. p. 113–121 (The Thera Foundation, London, 1990).
118. Robock, A. Volcanic eruptions and climate. *Reviews of Geophysics* **38**, 191–219 (2000).
119. Pyle, D.M. The application of tree-ring and ice-core studies to the dating of the Minoan eruption. In D.A. Hardy & A.C. Renfrew (eds.), *Thera and the Aegean world III. Volume three: chronology*. p. 167–173 (The Thera Foundation, London, 1990).
120. Scaillet, B., Clemente, B., Evans, B.W. & Pichavant, M. Redox control of sulfur degassing in silicic magmas. *Journal of Geophysical Research* **103**, 23937–23949 (1998).
121. Pyle, D.M. The global impact of the Minoan eruption of Santorini, Greece. *Environmental Geology* **30**, 59–61 (1997).
122. Michaud, V., Clocchiatti, R. & Sbrana, S. The Minoan and post-Minoan eruptions, Santorini (Greece), in the light of melt inclusions: chlorine and sulphur behaviour. *Journal of Volcanology and Geothermal Research* **99**, 195–214 (2000).
123. Cadoux, A., Scaillet, B., Bekki, S., Oppenheimer, C. & Druitt, T.H. Stratospheric Ozone destruction by the Bronze-Age Minoan eruption (Santorini Volcano, Greece). *Scientific Reports* **5**, 12243 (2015).
124. Panagiotakopulu, E., Higham, T., Sarpaki, A., Buckland, P. & Doumas, C. Ancient pests: the season of the Santorini Minoan volcanic eruption and a date from insect chitin. *Naturwissenschaften* **100**, 683–689 (2013).
125. Avnaim-Katav, S. et al. A multi-proxy shallow marine record for Mid-to-Late Holocene climate variability, Thera eruptions and cultural change in the Eastern Mediterranean. *Quaternary Science Reviews* **204**, 133–148 (2019).
126. Manning, J.G. et al. Volcanic suppression of Nile summer flooding triggers revolt and constrains interstate conflict in ancient Egypt. *Nature Communications* **8**, 900, doi: 10.1038/s41467-017-00957-y.
127. Quack, J.F. Gibt es in Ägypten schriftliche Quellen zum Thera-Ausbruch? In H. Meller, F. Bertemes, H.-R. Bork & R. Risch (eds.), *1600 – Kultureller Umbruch im Schatten des Thera-Ausbruches?/1600 – Cultural change in the shadow of the Thera-Eruption?* p. 221–233 (Landesmuseum für Vorgeschichte, Halle, 2013).
128. Karátson, D. et al. Towards reconstruction of the lost Late Bronze Age intra-caldera island of Santorini, Greece. *Scientific Reports* **8**, 7026 (2018).
129. Knappett, C., Rivers, R. & Evans, T. The Theran eruption and Minoan palatial collapse: new interpretations gained from modelling the maritime network. *Antiquity* **85**, 1008–1023 (2011).
130. Wiener, M.H. The isles of Crete? The Minoan thalassocracy revisited. In D.A. Hardy & A.C. Renfrew (eds.), *Thera and the Aegean world III. Volume one: archaeology*. p. 128–161 (The Thera Foundation, London, 1990).
131. Sarpaki, A. Akrotiri, Thera: glimpses of the countryside as seen through the archaeological and bioarchaeological data. Whispers of a dialogue. In D. Garcia, R. Orgeolet, M. Pomadere & J. Zurbach (eds.), *Country in the city: agricultural functions of protohistoric urban settlements (Aegean and Western Mediterranean)*. p. 77–92 (Oxbow Books, Oxford, 2019).
132. Evans, K.J. & McCoy, F.W. Precursory eruptive activity and implied cultural responses to the Late Bronze Age (LBA) eruption of Thera (Santorini, Greece). *Journal of Volcanology and Geothermal Research* **397**, 106868, doi.org/10.1016/j.jvolgeores.2020.106868 (2020).
133. Michailidou, A. The final settlement at Akrotiri on Thera: the buildings, the people, and the eruption. In H. Meller, F. Bertemes, H.-R. Bork & R. Risch (eds.), *1600 – Kultureller Umbruch im Schatten des Thera-Ausbruches?/1600 – Cultural change in the shadow of the Thera-Eruption?* p. 137–148 (Landesmuseum für Vorgeschichte, Halle, 2013).
134. Warren, P. & Hankey, V. *Aegean Bronze Age chronology* (Bristol Classical Press, Bristol, 1989).
135. Höflmayer, F. *Die Synchronisierung der minoischen Alt- und Neupalastzeit mit der ägyptischen Chronologie* (Österreichischen Akademie der Wissenschaften, Wien, 2012).
136. Luce, J.V. & Bolton, K. Thera and the devastation of Minoan Crete: a new interpretation of the evidence. *American Journal of Archaeology* **80**, 9–18 (1976).
137. Brogan, T.M. & Hallager, E. (eds.) *LM IB pottery: relative chronology and regional differences* (Monographs of the Danish Institute at Athens 11, The Danish Institute at Athens, Athens, 2011).
138. Torrence, R. Social responses to volcanic eruptions: a review of key concepts. *Quaternary International* **499**, 258–265 (2019).
139. Furumark, A. The settlement at Ialysos and Aegean history c. 1550–1450 BC. *Opuscula Archaeologica* **6**, 150–271 (1950).
140. Kemp. B.J. & Merrillees, R.S. *Minoan pottery in second millennium Egypt* (Philipp von Zabern, Mainz, 1980).
141. Gautschy, R. A reassessment of the absolute chronology of the Egyptian New Kingdom and its ‘brotherly’ countries. *Ägypten und Levant* **24**, 141–158 (2014).
142. Hornung, E., Krauss, R. & Warburton, D.A., eds. *Ancient Egyptian chronology* (Brill, Leiden, 2006).
143. Krauss, R. Egyptian chronology: Ramesses II through Shoshenq III, with analysis of the lunar dates of Tuthmoses III. *Ägypten und Levant* **25**, 335–382 (2015).
144. Popham, M.R. Late Minoan chronology. *American Journal of Archaeology* **74**, 226–228 (1970).
145. Warren, P.M. Absolute dating of the Bronze Age eruption of Thera (Santorini). *Nature* **308**, 494–493 (1984).
146. Betancourt, P.P. & Weinstein, G.A. Carbon-14 and the beginning of the Late Bronze Age in the Aegean. *American Journal of Archaeology* **80**, 329–348 (1976).
147. Bietak, M. Science versus archaeology: problems and consequences of high Aegean chronology. In M. Bietak (ed.), *The synchronization of civilisations in the eastern Mediterranean in the second millennium B.C. II.* p. 23–33 (Österreichischen Akademie der Wissenschaften, Wien, 2003).
148. Klontza-Jaklová, V. *What’s wrong?: hard science and humanities – tackling the question of the absolute chronology of the Santorini eruption* (Filozofická faculta, Masarykova univerzita, Brno, 2016).
149. Betancourt, P.P. Dating the Aegean Late Bronze Age with radiocarbon. *Archaeometry* **29**, 45–49 (1987).
150. Muhlenbruch, The absolute dating of the volcanic eruption of Santorini/Thera (periferia south Aegean/GR)—An alternative perspective. Praehistorische Zeitschrift **92**, 107–192 (2017).
151. Vinther, B.M. et al. A synchronized dating of three Greenland ice cores throughout the Holocene, Journal of Geophysical Research **111**, D13102, doi:10.1029/2005JD006921 (2006).
152. Badertscher, S. et al. Speleothems as sensitive recorders of volcanic eruptions – the Bronze Age Minoan eruption recorded in a stalagmite from Turkey. *Earth and Planetary Science Letters* **392**, 58–66 (2014).
153. Pearson, C.L. et al. Dendrochemical analysis of a tree-ring growth anomaly associated with the Late Bronze Age eruption of Thera. *Journal of Archaeological Science* **36**, 1206–1214 (2009).
154. Coulter, S.E. et al. Holocene tephras highlight complexity of volcanic signals in Greenland ice cores. *Journal of Geophysical Research* 117: D21303 (2012).
155. Taylor, R.E. *Radiocarbon dating: an archaeological perspective* (Academic Press, Orlando, 1987).
156. Warren, P.M. The Date of the Late Bronze Age eruption of Santorini. In D.A. Wardburton (ed.), *Time’s Up! Dating the Minoan eruption of Santorini*. p. 181–186 (Monographs of the Danish Institute at Athens 10, The Danish Institute at Athens, Athens, 2009).
157. Wiener, M.H. The state of the debate about the date of the Theran eruption. In D.A. Warburton (ed.), *Time’s Up! Dating the Minoan eruption of Santorini*. p. 197–206 (Monographs of the Danish Institute at Athens 10, The Danish Institute at Athens, Athens, 2009).
158. Wiener, M.H. Problems in the measurement, calibration, analysis, and communication of radiocarbon dates (with special reference to the prehistory of the Aegean world). *Radiocarbon* **54**, 423–434 (2012).
159. Wiener, M.H. Dating the Theran Eruption: Archaeological Science versus Nonsense Science. In T.E. Levy, T. Schneider & W.H.C. Propp (eds.), *Israel's Exodus in Transdisclipinary Perspective. Quantitative Methods in the Humanities and Social Sciences*. p. 131–143 (Springer, New York, 2015).
160. Bruins, H.J. & J. van der Plicht. The Minoan Santorini eruption and its ^14^C position in archaeological strata: preliminary comparison between Ashkelon and Tell el-Dab‘a. *Radiocarbon* **59**, 1295–1307 (2017).
161. Manning, S.W. Events, episodes and history: chronology and the resolution of historical processes. In L. Nevett & J. Whitley (eds.), *An Age of Experiment: Classical Archaeology Transformed (1976–2014)*. p. 119–137 (McDonald Institute for Archaeological Research, Cambridge, 2018).
162. Ehrlich, Y., Regev, L. & Boaretto, E. Radiocarbon analysis of modern olive wood raises doubts concerning a crucial piece of evidence in dating the Santorini eruption. *Scientific Reports* **8**, 11841 (2018).
163. Manning, S.W. Mediterranean radiocarbon offsets and calendar dates for prehistory. *Science Advances* **6**, eaaz1096 (2020).
164. Marketou, T., Facorellis, Y. & Maniatis, Y. New Late Bronze Age chronology from the Ialysos region, Rhodes. *Mediterranean Archaeology and Archaeometry* **1**, 19-29 (2001).
165. Manning, S.W. Beyond the Santorini eruption: some notes on dating the Late Minoan IB period on Crete, and implications for Cretan-Egyptians relations in the 15^th^ century BC (and especially LMII). In D.A. Warburton (ed.), *Time’s Up! Dating the Minoan eruption of Santorini*. p. 207–226 (Monographs of the Danish Institute at Athens 10, The Danish Institute at Athens, Athens, 2009).
166. Manning, S.W. & Bronk Ramsey, C. The dating of the earlier Late Minoan IA period: a brief note. In D.A Warburton (ed.), *Time’s Up! Dating the Minoan eruption of Santorini*. p. 227–245 (Monographs of the Danish Institute at Athens 10, The Danish Institute at Athens, Athens, 2009).
167. Soles, J.S. Radiocarbon results. In J.S. Soles, et al., *Mochlos IC. Period III. Neopalatial settlement on the coast: the Artisan’s Quarter and the Farmhouse at Chalinomouri. The small finds*. p, 145–149 (INSTAP Academic Press, Philadelphia, 2004).
168. Maniatis, Y. Radiocarbon dating of the Late Cycladic building and destruction phases at Akrotiri, Thera: new evidence. *The European Physics Journal Plus* **127**, 9, doi 10.1140/epjp/i2012-12009-y (2012).
169. Bayliss, A. Rolling out revolution: using radiocarbon dating in archaeology. *Radiocarbon* **51**, 123–147 (2009).
170. Halstead, P. & Jones, G. Agrarian ecology in the Greek islands: time stress, scale and risk. *Journal of Hellenic Studies* **109**, 41–55 (1989).
171. Halstead, P. *Two Oxen Ahead: Pre-Mechanized Farming in the Mediterranean* (John Wiley & Sons, 2014).
172. Rackham, O. & Moody, J. *The making of the Cretan landscape* (Manchester University Press, Manchester, 1997).
173. Asouti, E. Wood charcoal from Santorini (Thera): new evidence for climate, vegetation and timber imports in the Aegean Bronze Age. *Antiquity* **77**, 471–484 (2003).
174. Housley, R.A., Hedges, R.E.M., Law, I.A. & Bronk Ramsey, C. Radiocarbon dating by AMS of the destruction of Akrotiri. In D.A. Hardy & A.C. Renfrew (eds.), *Thera and the Aegean world III. Volume three: chronology*. p. 207–215 (The Thera Foundation, London, 1990).
175. Seierstad, I.K. et al. Consistently dated records from the Greenland GRIP, GISP2 and NGRIP ice cores for the past 104 ka reveal regional millennial-scale δ^18^O gradients with possible Heinrich event imprint. *Quaternary Science Reviews* **106**, 29–46 (2014).
176. Grudd, H., Briffa, K.R., Gunnarson, B.E. & Linderholm, H.W. Swedish tree rings provide new evidence in support of a major, widespread environmental disruption in 1628 BC. *Geophysical Research Letters* **27**, 2957–2960 (2000).
177. Helama, S. et al. Frost rings in 1627 BC and AD 536 in subfossil pinewood from Finnish Lapland. *Quaternary Science Reviews* **204**, 208–215 (2019).
178. Plunkett, G. et al. Trace element analysis of late Holocene tephras from Greenland ice cores. *Quaternary Newsletter* **143**, 10–21 (2017).
179. Wiener, M. Chronology going forward (with a query about 1525/4 B.C.). In E. Czerny, I. Hein, H. Hunger, D. Melman & A. Schwab (eds.), *Timelines: studies in honor of Manfred Bietak, Volume III*. p. 317–328 (Peeters, Leuven, 2006).
180. Vandersleyen, C. *Les guerres d’Amosis fondateur de la xviii^e^ dynasty* (Fondation Égyptologique Reine Élisabeth, Brussels, 1971).
181. Ryholt, K.S.B. *The political situation in Egypt during the Second Intermediate Period c. 1800-1550 B.C.* (Carsten Niebuhr Institute of Near Eastern Studies, University of Copenhagen; Museum Tusculanum Press, Copenhagen, 1997).
182. Oren, E.D. (ed.) *The Hyksos: new historical and archaeological perspectives* (University Museum, University of Pennsylvania, 1997).
183. Shaw, I. *The Oxford history of ancient Egypt* (Oxford University Press, Oxford, 2000).
184. Marée, M. (ed.) *The Second Intermediate Period (Thirteenth-Seventheeth Dynasties*). *Current research, future prospects* (Peeters, Leuven, 2000).
185. Bietak, M. *Avaris: the capital of the Hyksos. Recent excavations at Tell el-Dab‘a* (British Museum, London, 1996).
186. Broodbank, C. *The making of the middle sea. A history of the Mediterranean from the beginning to the emergence of the classical world* (Thames & Hudson, London, 2013).
187. Evans, A. *The Palace of Minos at Knossos* (vols. I-IV, Macmillan, London, 1921–1935).
188. Popham, M.R. Late Minoan chronology. *American Journal of Archaeology* **74**, 226 – 228 (1970).
189. Hägg, R. & Marinatos, N. (eds.) *The Minoan Thalassocracy: myth and reality. Proceedings of the third international symposium at the Swedish Institute in Athens, 31 May-5 June, 1982* (Svenska Institut i Athen/Paul Åströms Förlag, Stockholm/Göteborg, 1984).
190. Wiener, M.H. Realities of Power: The Minoan Thalassocracy in Historical Perspective. In R.B. Koehl (ed.), *Amilla: the quest for excellence. Studies presented to Guenter Kopcke in celebration of his 75th birthday*. p. 149–173 (INSTAP Academic Press, Philadelphia, 2013).
191. Cunningham, T. & Driessen, J. Site by site: combining survey and excavations data to chart patterns of socio-political change in Bronze Age Crete. In J.F. Cherry & S.E. Alcock (eds.), *Side by side survey: comparative regional studies in the Mediterranean world.* p. 101–113 (Oxford, 2004).
192. Macdonald, C.F., Hallager, E. & Niemeier, W.-D. (eds.) *The Minoans in the central, eastern and northern Aegean – new evidence* (Monographs of the Danish Institute at Athens 8, The Danish Institute at Athens, Athens, 2009)
193. Housley, R.A., Manning, S.W., Cadogan, G., Jones, R.E. & Hedges, R.E.M. Radiocarbon, calibration, and the chronology of the Late Minoan IB phase. *Journal of Archaeological Science* **26**, 159–171 (1999).
194. Wachsmann, S. *Aegeans in the Theban tombs* (Peeters, Leuven, 1987).
195. Rehak, P. Aegean natives in the Theban tomb paintings: the Keftiu revisited. In E.H. Cline & D. Harris-Cline (eds.), *The Aegean and the Orient in the Second Millennium*. p. 39–51 (Aegaeum 18, Université de Liège and the Program in Aegean Scripts and Prehistory, University of Texas at Austin, Liège & Austin).
196. Cunningham, T. 2007. Havoc: The Destruction of Power and the Power of Destruction in Minoan Crete. In J. Bretschneider, J. Driessen & K. van Lerberghe (eds.), *Power and Architecture: Monumental Public Architecture in the Bronze Age Near East and Aegean*. p. 23–43 (Orientalia Lovaniensia Analecta 156, Peeters Publishers, Leuven, 2007).
197. Manning, S.W. & Kromer, B. Considerations of the scale of radiocarbon offsets in the east Mediterranean, and considering a case for the latest (most recent) likely date for the Santorini eruption. *Radiocarbon* **54**, 449–474 (2012).
198. Rutter, J.B. Late Minoan IB at Kommos: a sequence of at least three distinct stages. In T.M. Brogan & E. Hallager (eds.), *LM IB pottery: relative chronology and regional differences*. p. 307–343 (Monographs of the Danish Institute at Athens 11, The Danish Institute at Athens, Athens, 2011).
